# Predictors of response to acetylcholinesterase inhibitors in dementia: A systematic review

**DOI:** 10.3389/fnins.2022.998224

**Published:** 2022-09-20

**Authors:** Federico Emanuele Pozzi, Elisa Conti, Ildebrando Appollonio, Carlo Ferrarese, Lucio Tremolizzo

**Affiliations:** ^1^Neurology Department, San Gerardo Hospital, Monza, Italy; ^2^School of Medicine and Surgery, University of Milano-Bicocca, Milan, Italy; ^3^Milan Center for Neuroscience (NeuroMi), University of Milano-Bicocca, Milan, Italy

**Keywords:** dementia, acetylcholinesterase inhibitors, systematic review, predictor, Alzheimer's disease

## Abstract

**Background:**

The mainstay of therapy for many neurodegenerative dementias still relies on acetylcholinesterase inhibitors (AChEI); however, there is debate on various aspects of such treatment. A huge body of literature exists on possible predictors of response, but a comprehensive review is lacking. Therefore, our aim is to perform a systematic review of the predictors of response to AChEI in neurodegenerative dementias, providing a categorization and interpretation of the results.

**Methods:**

We conducted a systematic review of the literature up to December 31^st^, 2021, searching five different databases and registers, including studies on rivastigmine, donepezil, and galantamine, with clearly defined criteria for the diagnosis of dementia and the response to AChEI therapy. Records were identified through the string: predict^*^ AND respon^*^ *AND (acetylcholinesterase inhibitors OR donepezil OR rivastigmine OR galantamine)*. The results were presented narratively.

**Results:**

We identified 1,994 records in five different databases; after exclusion of duplicates, title and abstract screening, and full-text retrieval, 122 studies were finally included.

**Discussion:**

The studies show high heterogeneity in duration, response definition, drug dosage, and diagnostic criteria. Response to AChEI seems associated with correlates of cholinergic deficit (hallucinations, fluctuating cognition, substantia innominate atrophy) and preserved cholinergic neurons (faster alpha on REM sleep EEG, increased anterior frontal and parietal lobe perfusion after donepezil); white matter hyperintensities in the cholinergic pathways have shown inconsistent results. The K-variant of butyrylcholinesterase may correlate with better response in late stages of disease, while the role of polymorphisms in other genes involved in the cholinergic system is controversial. Factors related to drug availability may influence response; in particular, low serum albumin (for donepezil), CYP2D6 variants associated with reduced enzymatic activity and higher drug doses are the most consistent predictors, while AChEI concentration influence on clinical outcomes is debatable. Other predictors of response include faster disease progression, lower serum cholesterol, preserved medial temporal lobes, apathy, absence of concomitant diseases, and absence of antipsychotics. Short-term response may predict subsequent cognitive response, while higher education might correlate with short-term good response (months), and long-term poor response (years). Age, gender, baseline cognitive and functional levels, and APOE relationship with treatment outcome is controversial.

## Introduction

More than 55 million people suffer from dementia worldwide, with the number expected to rise up to 78 million within the current decade and to 139 million by 2050. Alzheimer's disease (AD) represents the most frequent diagnosis, in about 60–70% of cases (World Health Organization, 2021). Despite the thriving amount of research about possible disease-modifying drugs, and taken into account the controversy about amyloid-targeted monoclonal antibodies [approved by the Food and Drug Administration (Dunn et al., 2021), but not by the European Medicines Agency at the time of writing (Mahase, 2021)], there is currently no cure for neurodegenerative dementias, which reflects the complexity of the mechanisms and systems involved in their pathogenesis. Among these, one of the most extensively studied is the cholinergic system, with second-generation acetylcholinesterase inhibitors (AChEI), namely rivastigmine, galantamine, and donepezil, still representing the mainstay of therapy in AD, dementia with Lewy bodies (DLB), and Parkinson's disease dementia (PDD), and, although less widely accepted, even vascular dementia (VaD). The effect of these drugs in rarer dementias is less clear (Li et al., 2015). Although widely used, especially in mild and moderate phases of diseases, not all patients benefit from AChEI therapy, and a significant proportion of them experience adverse effects which lead to drug switching or interruption. Clinical response is seen in 30–60% of patients across different studies, and an enormous body of literature exists on possible predictors of such response. These predictors can be broadly summarized as correlates of cholinergic deficit together with relative preservation of the cholinergic pathways, upon which AChEI could act, and factors influencing drug metabolism and availability. It is noteworthy that AChEI seem not to be disease-specific, but rather mechanism-specific, as all neurodegenerative diseases with an established cholinergic involvement could theoretically benefit from their use. Yet, AChEI are licensed and reimbursed by National Health Systems of most countries only in mild-to-moderate AD.

The available evidence shows that these drugs seem to have only a symptomatic effect, and only recently an interplay between them and the putative neuropathogenic mechanisms of AD, namely amyloid aggregation, tau phosphorylation, and neuroinflammation, has been investigated. Indeed, some authors report that these drugs may exhibit a disease-modifying effect, however modest, by reducing the rate of atrophy in the hippocampus, cortex, and basal forebrain, and possibly delaying the progression of mild cognitive impairment (MCI) to dementia (Hampel et al., 2019).

The duration of AChEI's symptomatic or proposed disease-modifying effect is poorly understood, with only a few studies dealing with the long-term efficacy of AChEI, which have been traditionally thought to act relevantly only for 6 months. However, a mechanism for such long-lasting effects should necessarily not be due to an action on the cortical cholinergic projections, which are almost entirely lost in late stages of the disease together with a down-regulation of cholinergic receptors, but maybe it operates through an effect on subcortical cholinergic pathways in the basal ganglia and the thalamus (Hampel et al., 2018). It is noteworthy that the most recent NICE guidelines on AChEI proposed to continue treatment only when it is considered to be having a worthwhile effect on cognitive, global, functional, or behavioral symptoms, with the choice of timing of patients' review left to the clinician's judgment (NICE, 2011).

### The cholinergic pathways

A few neurodegenerative dementias, at least in their initial phases, involve the neurodegeneration of the cholinergic system in the brain. This would result in typical cognitive symptoms, mostly in the attentional and memory domain. Such involvement has been extensively studied in AD, for which the cholinergic hypothesis was first introduced in 1982 in a review by Bartus, following seminal works published in the 60's and the 70's, with the observation of cortical depletion of choline acetyl-transferase in AD brain samples, the positive effect on short-term memory of physostigmine in aged primates, and the opposite effect of anti-cholinergic agents (Bartus et al., 1982). Subsequent papers demonstrated that the nucleus basalis of Meynert (NB) is the source of cholinergic innervation of the cortex, and that neurofibrillary neurodegeneration in the posterior NB is present at very early stages of AD (and even precedes the onset of symptoms) and correlates with its severity. This led to the demonstration of the positive symptomatic effect on memory of the first-generation AChEI, such as tacrine and metrifonate, followed by second-generation AChEI, with a better safety profile (Hampel et al., 2019).

It is important to note that the cholinergic hypothesis does not constitute an etiological explanation of the disease, but rather a common target of multiple heterogeneous pathophysiological factors, which have not been completely elucidated to date. These include amyloid pathology, tau phosphorylation, neuroinflammation, vascular change, calcium influx, and nerve growth factor (NGF) dysregulation. For instance, the NB and medial septum neurons fully depend on NGF for their trophism, and therefore impairment of its retrograde transport from the cortex to the basal forebrain or its conversion from pro-NGF could induce cholinergic atrophy. All these mechanisms exhibit an important and still poorly understood crosstalk. Notably, it is still debated whether APOE-ε4, the major risk factor for late-onset AD, affects the NB function, and how (Hampel et al., 2018).

**Figure 1:**
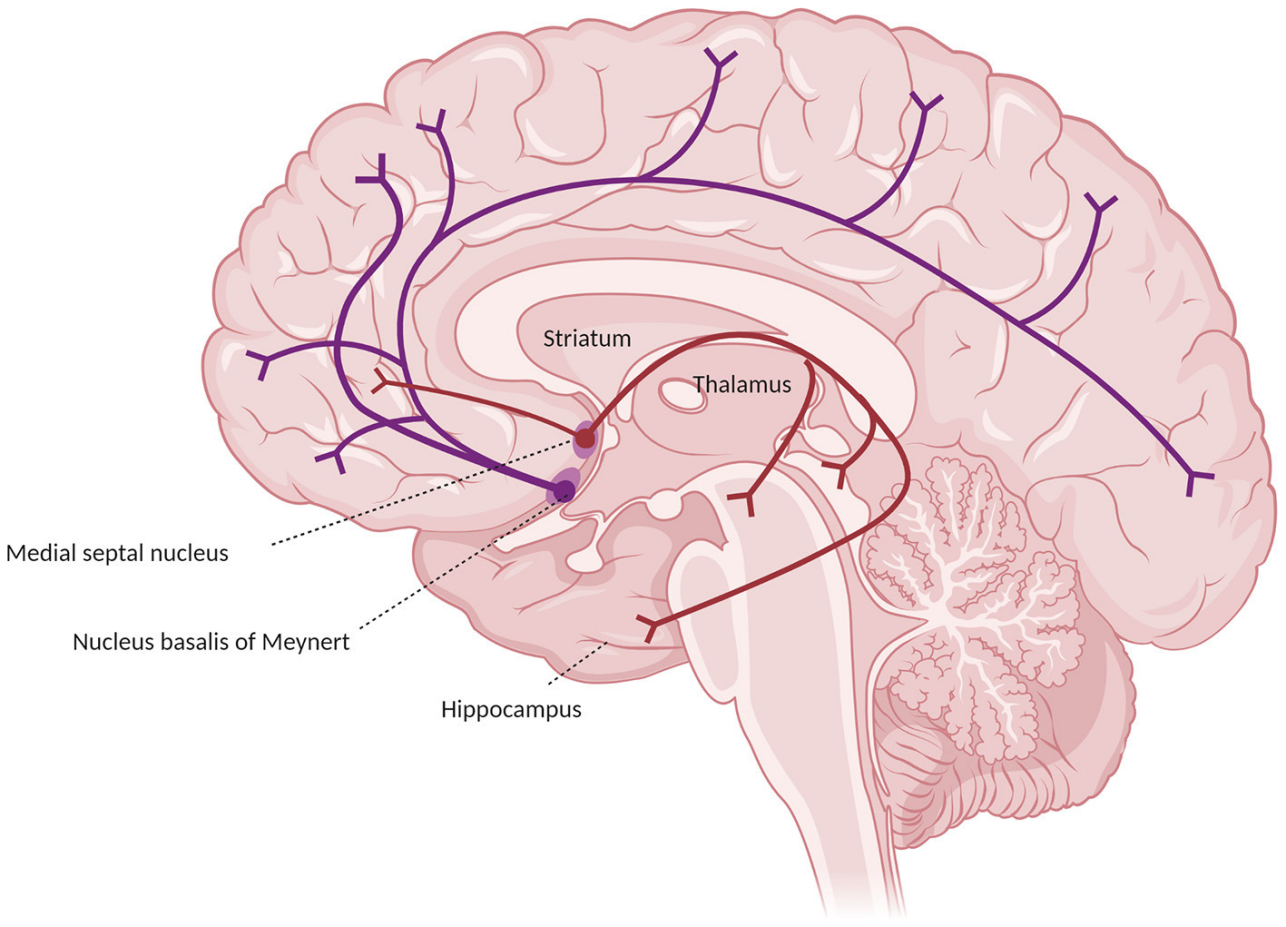
Major cholinergic pathways in the brain, starting from the basal prosencephalon, showing the projections from the nucleus basalis of Meynert (in blue) and from the medial septal nuclei (in red).

In humans and primates the central actor in the cholinergic system impairment in AD is the basal forebrain, which receives cortical input from limbic and paralimbic areas and projects to several brain regions. Such areas include the hippocampus (which receives axons from Ch1 medial septum neurons through the fornix and from Ch2 vertical limb nucleus of the diagonal band neurons), the olfactory bulb (from Ch3 horizontal nucleus of the diagonal band nucleus through the olfactory tract), and the whole cortex and amygdala (from Ch4 neurons of the NB). Together, these connections provide the extra-thalamic component of the ascending reticular activating system (Selden et al., 1998; Hampel et al., 2019). The Ch4 neurons include also the substantia innominata and the magnocellular preoptic nucleus (Ballinger et al., 2017). In the classical description, the projections from the NB reach the amygdala through the stria terminalis and the ventral amygdalofugal pathway, and the cortex in two major bundles, the medial and the lateral cholinergic pathways. The former originates from the anterior Ch4 neurons, running posteriorly around the corpus callosum within the cingulum to reach the retrosplenial white matter, reaching in its course the orbitofrontal, subcallosal, cingulate, pericingulate, and retrosplenial cortices. The lateral cholinergic pathway can be divided into a capsular division, coursing through the medial aspect of the external capsule to reach the dorsal frontoparietal and inferior temporal cortices, and a perisylvian division, traveling through the claustrum, and reaching the frontoparietal opercular cortex, the superior temporal cortex, and the insula. The two pathways merge anteriorly in the orbitofrontal cortex and posteriorly in the occipital lobe (a schematic representation of the cholinergic pathways is provided in Figure 1) (Selden et al., 1998). Recently, the once-thought diffuse cortical projections from the NB have been revealed to be organized into cortical target-specific groups of neurons, which receive topographically distributed inputs. It seems that Ch1–Ch3 neurons and anterior-medial portions of the Ch4 neurons of the NB (which constitute the anterior basal forebrain) project predominantly to the hippocampus, ventromedial prefrontal, and retrosplenial/posterior cingulate cortices. Conversely, more posterior-lateral cell clusters of the basal forebrain (including the rest of the NB, constituting the posterior basal forebrain) preferably project to the thalamus (even though it receives most of its cholinergic inputs from the midbrain), the dorsal anterior cingulate cortex, and the insula. The two projections considerably overlap in the medial prefrontal, posterior lateral orbitofrontal cortex, temporal pole, hippocampus, and amygdala (Fritz et al., 2018). These limbic and paralimbic areas are also the only cortical areas that provide inputs to the basal forebrain. The functional connections of the anterior basal forebrain resemble a described medial default mode network subsystem (also called “episodic memory network”), while the connections of the posterior basal forebrain with the insula and the dorsal anterior cingulate cortex are similar to the ventral attention network. This may indicate a separate role of such projections in memory and attention, respectively (Fritz et al., 2018).

Neurofibrillary degeneration of the posterior Ch4 neurons of the NB is present even in cognitively unimpaired elderly, progressing through the MCI and AD stages (Hampel et al., 2019). Notably, hallmarks of AD pathology, such as neurofibrillary tangles, seem to be found early in the course of AD, even at Braak's stage 0 (Rey et al., 2018).

Muscarinic and nicotinic receptors might act differently during the course of disease. The latter, especially the α-7 nicotinic receptors (α7-nAChR), are lost in cortical areas involved in cognition in AD patients. Although some drugs acting on cholinergic receptors have shown encouraging data, and even possible disease-modifying effects through the reduction of amyloid burden, they seem to be far from entering the therapeutic panorama (Hampel et al., 2019).

It should be noted that NB degeneration is also present in PDD and DLB, in which it is associated with Lewy bodies and synucleinopathy, rather than neurofibrillary tangles (Hampel et al., 2018).

### The cholinergic synapse

**Figure 2:**
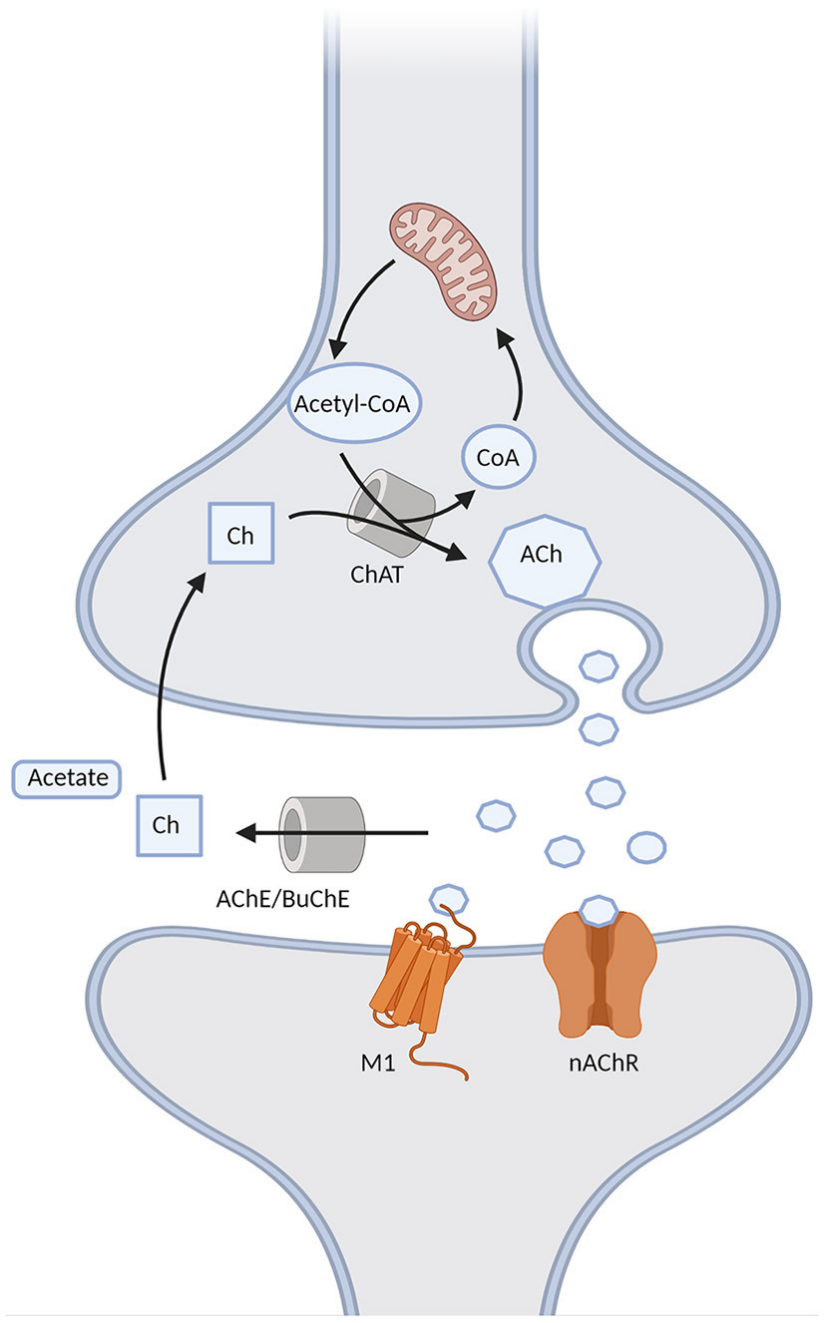
Schematic representation of the cholinergic synapse. Acetylcholine (ACh) is formed through the choline acetyltransferase (ChAT)-mediated binding of choline (Ch) with the acetyl group of acetyl-CoA provided by the mitochondria. It is then released into the synapse, where it binds to either muscarinic M1 or nicotinic (nAChR) receptors, and it is then degraded into Ch and acetate by acetylcholinesterase (AChE) or butyrrylcholinesterase (BuChE). Choline can be later recycled into the pre-synaptic terminal to form ACh.

Cholinergic synapses are ubiquitous in the brain. Acetylcholine (ACh) is synthesized from choline and acetyl coenzyme A, a byproduct of glycolysis, by choline acetyltransferase (ChAT), and then released into the synapse, where it binds to cholinergic muscarinic (metabotropic) and nicotinic (ionotropic) post-synaptic receptors. Choline acetyltransferase is subsequently removed from the synaptic cleft by acetylcholinesterase (AChE) and, to a lesser degree in the brain, by butyrylcholinesterase (BuChE) (Vaknine and Soreq, 2020). However, during the progression of AD there is a 67% reduction in AChE [although the isoform G1 tends to increase, together with a reduction of the isoform G4 (Siek et al., 1990)] and a 165% increase in BuChE activity in the temporal lobe and hippocampus (Marucci et al., 2020). Apart from AChE and BuChE, other mechanisms are involved in the termination of cholinergic transmission, such as the diffusion of ACh from the synaptic cleft and a negative feedback mechanism mediated by presynaptic M2 and M4 receptors. The latter limit ACh release following a rapid increase in ACh levels, but may be in turn overcome by muscarinic antagonists, the concomitant use of which may represent a future potential strategy in AD (Mohr et al., 2015). Moreover, ACh induces nAChR desensitization, which is especially rapid for α7-nAChR. Interestingly, the desensitization of α7-nAChR may be overcome by choline, a selective full agonist of such receptors. Therefore, the generation of choline by ACh hydrolysis may extend cholinergic signaling beyond the synaptic region after diffusion due to its long clearance, which may be relevant for AChEI therapy in AD (Borroni and Barrantes, 2021). The main elements of the cholinergic synapse are represented in Figure 2.

The cholinergic lesion in AD is mostly presynaptic, resulting from the degeneration of NB cholinergic projections. Thus, incrementing the quantity of ACh acting on the post-synaptic receptors currently represents the main strategy to compensate for this loss. However, following presynaptic deafferentation, there is also down-regulation of post-synaptic nicotinic receptors (Sumirtanurdin et al., 2019), while M1 receptors do not undergo a reduction, but rather a dysfunction (Hampel et al., 2018). It seems that the activity of muscarinic M1 post-synaptic receptors is independent of the integrity of the cholinergic terminals (Giacobini et al., 2022), which supports the notion that M1 receptors might be a suitable target for new cholinergic approaches. Presynaptic muscarinic receptors M2 are decreased as well [notably, M2 receptors are downregulated also in the context of persistently high ACh levels (Mohr et al., 2015)].

α7-nAChR (encoded by CHRNA7) are involved in long-term potentiation in the hippocampus, which is crucial for learning and memory. They may also have a neuroprotective effect against tau hyperphosphorylation and amyloid-beta toxicity through several downstream mechanisms, including anti-apoptotic proteins and enhancement of anti-inflammatory pathways through NF-kB downregulation (Hampel et al., 2018). Such anti-inflammatory effects are thought to be exerted through bidirectional vagus nerve messages exchanged by the body and the brain (Vaknine and Soreq, 2020). However, the crosstalk between the peripheral and central cholinergic systems is still largely unclear.

**Figure 3:**
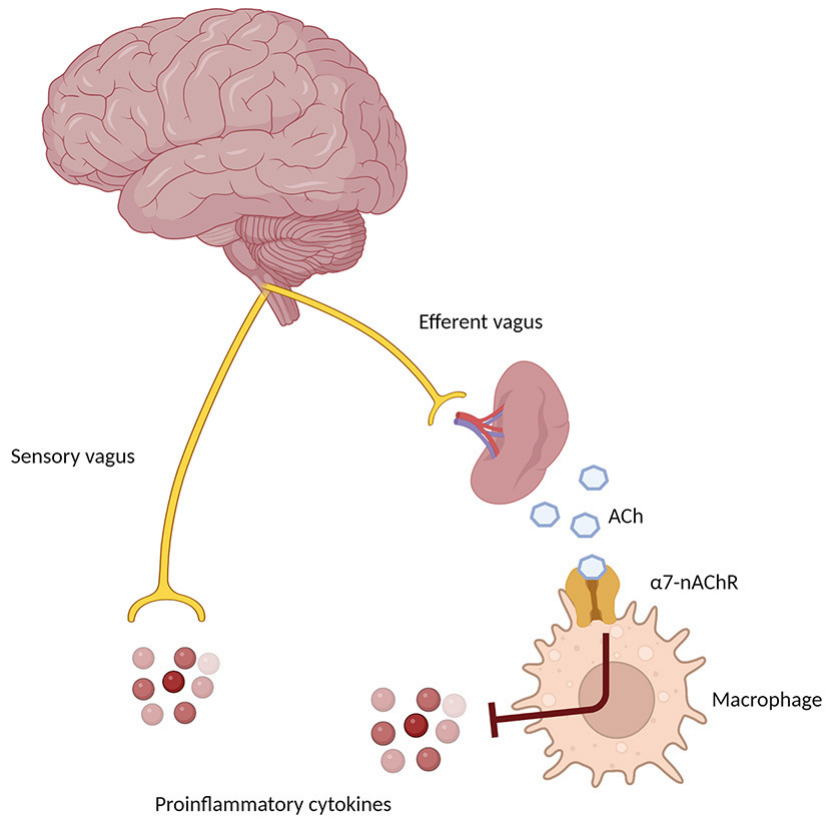
Schematic representation of the Cholinergic Anti-Inflammatory Pathway (CAIP), showing the afferent arm through the sensory vagus nerve, sensing proinflammatory signals in the periphery, and the efferent anti-inflammatory arm, which through the α7 nicotinic receptor on peripheral macrophages and immune cells determines a down-regulation of inflammatory pathways.

Nevertheless, the peripheral immune system is clearly involved in the pathogenesis and progression of AD (Angiulli et al., 2021). In particular, an immune-brain axis exists that, upon the release of soluble inflammatory mediators by activated microglial cells, contributes to lowering the extent of inflammatory processes. On the other hand, pro-inflammatory cytokines released by peripheral cells reach CNS by penetrating the blood-brain barrier and contribute to increasing neuroinflammation (Fiala et al., 1998; Krstic et al., 2012). The cholinergic modulation of inflammation via the vagus nerve is known as the Cholinergic Anti-Inflammatory Pathway (CAIP, Figure 3). Inflammatory molecules in the periphery are detected by the afferent arc of the vagus nerve and reach the nucleus tractus solitarius, which leads to the downregulation of inflammatory processes through the involvement of α7-nAChR on peripheral macrophages. The efferent vagus nerve transmits signals to the spleen that eventually result in the synthesis and release of ACh, whose binding to α7-nAChR on peripheral macrophage leads to the inhibition of cytokine production (Tracey, 2009). To further complicate this matter, Gault et al. discovered that the gene encoding the α7-nAChR monomer is partially duplicated between exons 5–10. Moreover, they identified four new exons derived from the FAM7 sequence (Gault et al., 1998). The fused, partially duplicated α7-nAChR sequence and new FAM sequence create a new α7nAChR gene, now called CHRFAM7A. It encodes for a 45 kD protein mainly expressed in human leukocytes. This duplicated α7-nAChR lacks signal peptide and ACh binding domain. Moreover, the CHRFAM7A gene product differs from classical α7-nAChR, since it is not able to bind α-bungarotoxin and evoke currents after ACh or nicotine treatment (Villiger et al., 2002). Due to its properties, CHRFAM7A is a dominant negative inhibitor of α7-nAChR, preventing its trafficking to the cellular membrane. The gene encoding for duplicated α7-nAChR is exclusively human and it is present in one or two copies in 95% of the population. Benfante et al. demonstrated that donepezil differently modulates CHRFAM7A and CHRNA7 responsiveness to LPS, suggesting that this receptor may have a role in the inflammatory response of innate immunity (Maroli et al., 2019). Due to this phenomenon, it is conceivable that the CHRFAM7A should be considered a potential factor modulating treatment response to AChEI, although more data are necessary before concluding on this issue.

Another emergent interesting field is related to RNA, with a recent study showing different profiles of AChE mRNA expression in AD patients compared to healthy controls (Barbash et al., 2017). However, further research is needed to establish the existence of mRNA-associated predictors.

All these different actors in the cholinergic synapse may be altered by polymorphisms in relevant genes, with consequent influences on AChEI effectiveness.

### Neuropharmacological aspects of AChEI

All AChEI prevent the hydrolysis of ACh into choline and acetate mediated by either AChE or BuChE, thus increasing the presence of ACh in the synaptic cleft. As tacrine is no longer in use due to its hepatotoxicity, we are left with only three AChEI, with negligible differences in their efficacy profiles. Donepezil is a reversible highly selective inhibitor of AChE, available in 5 or 10 mg/day tablets, although the 23 mg/day formulation has been approved by the FDA for moderate-to-severe AD. It is metabolized mostly by CYP2D6, with a less studied contribution from CYP3A4. Its roles, not completely elucidated to date, also include non-cholinergic effects, namely an effect on α-1 adrenergic receptors, improvement of neuronal plasticity, anti-inflammatory effects, improvement of cerebral blood flow, decrease of amyloid precursor protein and excitotoxic damage, modulation of oxidative stress, influence on AChE isoform expression, and interaction with the nicotinic receptors (Marucci et al., 2020). One of these interesting actions is the modulation of the endogenous immune response in AD, possibly increasing naturally occurring auto-antibodies against Aβ through a shift toward a Th2 phenotype via direct interaction with α7-nAChR (Conti et al., 2016). Its putative neuroprotective effect would thus rely on the effect on Aβ-induced toxicity via the α7-nAChR and the PI3K-Akt pathway, and the suppression of IL1-b and COX-2 expression in the brain and the spleen (Marucci et al., 2020).

Rivastigmine is a pseudo-irreversible inhibitor of both AChE and BuChE. It is available both in patches (up to 9.5 or 13.3 mg/day) and capsules (up to 6 mg twice a day). Its preference for the G1 isoform of AChE, still preserved in severe AD stages (Siek et al., 1990), may explain the sometimes reported effectiveness of rivastigmine even in late disease. It is not metabolized in the liver through the CYP450 system, but rather via cholinesterase-mediated hydrolysis, and it undergoes renal excretion (Marucci et al., 2020).

Galantamine, a natural derivative isolated from the bulbs and the flowers of *Galanthus woronowii*, exerts its effects by selectively and reversibly inhibiting AChE, with little peripheral effects, and by positively allosterically modulating the nicotinic cholinergic receptors (Giacobini et al., 2022). This additional feature may contribute to the clinical effectiveness of galantamine because of the known relation of the severity of AD with the loss of such receptors (Marucci et al., 2020). It also seems to have a positive effect through muscarinic M1 receptors in the hippocampus, where it enhances the proliferation of progenitor cells in the subgranular zone, and through the nicotinic receptors, influencing the survival of the newly divided cells in the granular layer. Through the inhibition of the NF-kB pathway via the α7-nAChR, galantamine has been shown to exert an anti-inflammatory effect as well (Marucci et al., 2020). Its brain availability through oral administration is quite low, and other routes have been studied, including intranasal formulations. However, galantamine is currently available only in tablets (up to 12 mg twice a day, or 24 mg/day in the extended-release formulation). Similar to donepezil, it is metabolized by CYP2D6 and CYP3A4 in the liver (Marucci et al., 2020). The pharmacological properties of the different AChE are summarized in Table 1.

**Table 1:** Pharmacological properties of available acetylcholinerase inhibitors.

	**Reversibility**	**Selectivity**	**Formulations**	**Dosage**	**Metabolism**	**Excretion**
Donepezil	Reversibile	AChE	Tablets	5–10 mg 23 mg in some countries	CYP2D6, CYP3A4	Hepatic
Rivastigmine	Pseudo-irreversible	AChE, BuChE	Patches	4.6–9.5 mg 13.3 mg in some countrie	ChE-mediated hydrolysis	Renal
			Capsules	6 mg ×2 or 12 mg		
Galantamine	Reversible	AChE	Tablets	12–24 mg	CYP2D6, CYP3A4	Hepatic

### Definition of response

There is extreme heterogeneity in studies investigating possible predictors of response to AChEI. Notably, a striking variability exists in the definition of response, the inclusion and exclusion criteria, and the duration of follow-up. Before analyzing the different predictors identified in the literature, it is worth acknowledging these factors and discussing their implications.

Firstly, the definition of response involves an inevitable discussion about realistic expectations with AChEI therapy, which may be mostly symptomatic. An effect has been described in cognition, function, and global impression, but the majority of papers define the response only in the cognitive domain, generally using MMSE and/or ADAS-Cog. However, according to updated NICE guidelines, the MMSE alone is not sufficient to effectively assess cognitive changes, since it has been introduced as a screening tool for AD, and it suffers from significant floor and ceiling effects, limiting its usefulness in the longitudinal evaluation of treatments. The same issues are shared by the ADAS-Cog scale (Palmqvist et al., 2010). MMSE could also show limited sensitivity in people with higher levels of education, and may not be culturally independent. This may limit its use in low-income countries, which are also extremely under-represented in clinical trials (Palmqvist et al., 2010; NICE, 2011).

According to these guidelines, patients undergoing treatment with AChEI should be reviewed regularly with a cognitive, global, functional, and behavioral assessment by an appropriate specialist team, seeking caregivers' views on the patient's condition as well. Moreover, experts agreed that even all those measures may not capture the entirety of responders, as relevant benefits would also include maintaining mood, being able to cope and interact with others, and functional activities that might not be scored on currently used scales, such as being able to pick up the phone or switch on the television, as well as maintaining aspects of personal identity (NICE, 2011).

As said, therapeutic response can be measured in different ways, with some papers performing proper responder analyses dichotomizing the outcomes, and some others treating evaluating scales as continuous variables, seeking associations between certain predictors and changes in outcome measures. The most typical definition of response used in responder analysis is “improvement” (or, sometimes, “no deterioration”). Such improvement should encompass cognition (measured with ADAS-Cog and/or MMSE) and at least one other measure of global response (mostly measured with Clinician's Interview-Based Impression of Change with Global Input—CIBIC+, CDR-SB, or Global Deterioration Scale—GDS), function (measured with ADL and/or IADL, with the limitations described above) and behavior (almost exclusively measured with NPI). However, as already discussed, some papers only evaluate cognitive or global improvement. Another popular definition of response is “less than expected decline,” which usually refers to a slower decrease in cognition compared to placebo or natural history, as evaluated with ADAS-Cog or MMSE. Finally, some papers defined response as persistence of long-term clinical benefits after an initial short-term response, defined with any of the previously described criteria (Burns et al., 2008).

It could be argued that the improvement criteria do not necessarily imply a clinically significant response, especially in light of the known modest effect of AChEI. The latter has been defined as an improvement of a least 4 points on the ADAS-Cog (roughly corresponding to a 2 points improvement on MMSE) by an expert panel meeting convened by the FDA in 1989, while the EMA adds to this definition the absence of worsening on CIBIC+ and ADL (Raschetti et al., 2005). Different studies used different cut-offs to separate responders from non-responders, even with the same scales, thus limiting the comparability of their results. An interesting field of research involves the identification of biomarkers of response to AChEI, which might include plasma amyloid-beta levels after treatment, and SPECT, MRI, and PET imaging results. This could possibly decrease the level of subjectivity in the current response assessment models (Miettinen et al., 2015).

As a side note, other ways to define response have been occasionally utilized, such as time to institutionalization, which is relevant especially in high-income countries (Wattmo et al., 2018), increased life expectancy (Zhu et al., 2013) [indeed, AChEI seem to be associated with reduced risk of myocardial infarction and overall mortality (Giacobini et al., 2022)] and health-related quality of life. In this regard, NICE guidelines acknowledge the importance of utility values for health-related quality of life reported by patients themselves (in early stages of the disease) and by caregivers (in more advanced stages) as proxy responses (NICE, 2011). However, a Cochrane meta-analysis performed in 2018 failed to find any difference between donepezil and placebo in terms of quality of life, even though the conclusion was based on only two studies (Birks and Harvey, 2018).

Other important questions include how to consider adverse events, which could lead to AChEI switching or discontinuation, and drop-outs in clinical trials, which can be as high as 65% and increasing with follow-up duration. Both issues could lead to obvious biases, resulting in an overestimation of treatment response if they are not accounted for. Accordingly, some studies regarded drop-outs as non-responders (Patterson et al., 2011; Miranda et al., 2015).

However, given the progressive nature of neurodegenerative dementias and the high burden placed on caregivers, it could be useful to identify patient-relevant outcomes to define meaningful response criteria, and involve families and caretakers in the evaluation of response; some projects are already in place to address such challenges (Gallacher et al., 2019). Indeed, a slight improvement in cognition might not be relevant at all for elderly patients, while functional and identity preservation, along with the less considered aspects listed above, may be comparably more important for subjects suffering from dementia. This is in line with evidence showing the cruciality of involvement of patients and caregivers in clinical guidelines (Armstrong et al., 2018).

The second important issue is the heterogeneity of inclusion and exclusion criteria across studies. Most of the works available in the literature rely on clinical criteria for patient selection, such as the criteria for probable or possible AD of the National Institute of Neurological and Communicative Diseases and Stroke and Alzheimer's Disease and Related Disorders Association (NINCDS-ADRDA), or the Diagnostic and Statistical Manual of Mental Disorders, 4th version (DSM-IV). However, clinical criteria may be outdated, at least in AD, as recent years are witnessing a shift toward the use of pathology-derived biomarkers such as cerebrospinal fluid amyloid and phospho-tau or amyloid-PET, which yield comparably higher sensitivity and specificity (Gaugler et al., 2013). Yet, only a few studies on predictors of response to AChE used such biomarkers, with obvious consequences on the reliability of their results. Moreover, while randomized controlled trials (RCT) notoriously tend to deal with highly selective populations, which may not be representative of real-life patients, even real-world studies tend to exclude patients with mixed forms of dementia or concurrent medication use; however, these are the majority of patients encountered in clinical practice. The physician is thus left with a huge body of evidence, which in the end might not be useful to support clinical choices for the individual patient.

The populations included in the clinical studies may have different genetic backgrounds, and observations made for Caucasian patients may not be generalizable to Asian ones, with contrasting results in the literature. This stresses even more the need for clinical trials and studies on under-represented populations, such as racial minorities in high-income countries and people in low-income countries. To date, there are no studies investigating possible predictors of response to AChEI for African, Middle-Eastern, Indian, and most of Latin American patients, even though the majority of patients affected by neurodegenerative dementias in the next decades is expected to be found in these populations.

The third issue regards the appropriate timing for the assessment of response to AChEI, with most studies focusing on the initial period of 3–9 months, and only a few works exploring outcomes and predictors of response past the first year of therapy. Acetylcholinesterase inhibitors (AChEI) are expected to exert their maximum efficacy in the first half-year of treatment, but there is still considerable debate on the matter. The frequency of assessment is another factor to be considered, as cognitive tests tend to suffer from repetition habituation, which might lead to overestimation of response when they are performed too close in time to each other. A period of 6 months for re-assessment is generally considered optimal in the vast majority of studies.

Another factor that hinders the generalizability of results is the heterogeneity of treatment used, the different dosages (for instance, most trials with donepezil conducted in Japan used the 5 mg tablets) and the different dose titration schemes. However, as already observed, the resulting differences in clinical efficacy might be negligible. Finally, most of the available evidence on predictors has not been subject to cross-validation, thus limiting the validity of the results (Ohnishi et al., 2014).

### Aims of the work

A few reviews and meta-analyses are available for specific categories of predictors in specific diseases, but a comprehensive review on the whole topic is lacking. Our aim in this paper is to perform a systematic review of the literature about predictors of response to second-generation AChEI in dementia, in order to provide physicians and researchers with updated data to base clinical decisions and research efforts.

As already stated, AChEI seems to function when there is cholinergic depletion, but not when there is extensive neuronal loss within the cholinergic system (Tei et al., 2008). Accordingly, different categories of predictors could be theoretically identified based on their putative mechanism, and the discussion will be divided based on their categorization.

## Methods

This systematic review has been conducted in accordance with the principles of the Preferred Reporting Items for Systematic Reviews and Meta-Analyses (PRISMA) 2020 statement. The full checklist is available in the Supplementary material (Page et al., 2021).

Inclusion criteria were as follows:

- Studies on rivastigmine, donepezil, or galantamine;
- Clearly stated response criteria to AChEI therapy;
- Established diagnostic criteria for dementia;
- Enough data to assess risk of bias;
- Studies on human subjects, including cohort studies (prospective or retrospective), RCTs, cross-over studies, cross-sectional trials;
- Articles written in one of the following languages: English, Italian, German, French, Romanian, Russian, Dutch, Spanish, Portuguese.

Exclusion criteria were as follows:

- Studies on tacrine, physostigmine, metrifonate, memantine (unless a separate analysis was conducted to isolate the effect of AChEI);
- Review articles or meta-analyses, case reports, case series, editorials.

Review and meta-analysis reference lists were checked to identify additional eligible studies and to elucidate theoretical aspects in the discussion.

We conducted electronic searches for eligible studies within each of the following databases, up to December 31st, 2021: Cochrane Central Register of Controlled Trials (CENTRAL), MEDLINE (accessed via PubMed), Embase, Scopus, and Web of Science. In addition, we checked the reference list of all screened study reports to identify further eligible studies.

Records were identified in each database with the following search strings: predict^*^ AND respon^*^ *AND (acetylcholinesterase inhibitors OR donepezil OR rivastigmine OR galantamine)*. No additional filters were used in order to maximize sensitivity.

Citations identified from the literature searches were imported to Mendeley and duplicates were automatically removed by the software. Then, two reviewers (FEP and LT) independently screened the titles and abstracts of all the records. In case of disagreements about eligibility, a consensus was reached through discussion. Full-texts of all potentially eligible studies, reviews, and meta-analyses were retrieved, and their reference lists checked. Data extraction from eligible studies was performed by one reviewer (FEP), and all proceedings were checked by a second reviewer (LT). We extracted data about authors, year, country, study design, number of participants, treatment (type of AChEI and dose), diagnostic criteria, response definition, findings of the study, identified predictors, and limitations.

The risk of bias was assessed with the Cochrane risk of bias (RoB) tool 2.0 for RCTs (Sterne et al., 2019). For observational studies and *post-hoc* analysis of RCTs, no available tools were judged appropriate to assess risk of bias in studies investigating possible predictors of response. Accordingly, studies were critically appraised based on the key criteria for observational studies identified by the Grading of Recommendations Assessment, Development, and Evaluation (GRADE) working group, adapted for the purposes of the current review. These include failure to develop and apply appropriate eligibility criteria, flawed measurement of either predictors or response, failure to adequately control confounding factors, and incomplete follow-up (a drop-out rate of <20% was judged acceptable) (Guyatt et al., 2011). The risk of bias was considered high if two or more of these items were present, intermediate if only one was present, and low if no item was present. Two reviewers (FEP and LT) independently critically appraised the eligible studies; in case of disagreements, a consensus was reached through discussion.

Since the identified studies were extremely heterogeneous, and given the availability of meta-analyses on specific predictors, we decided to present the results of the current review narratively.

A protocol for this systematic review was developed before the research began. However, this review was not registered.

## Results

**Figure 4:**
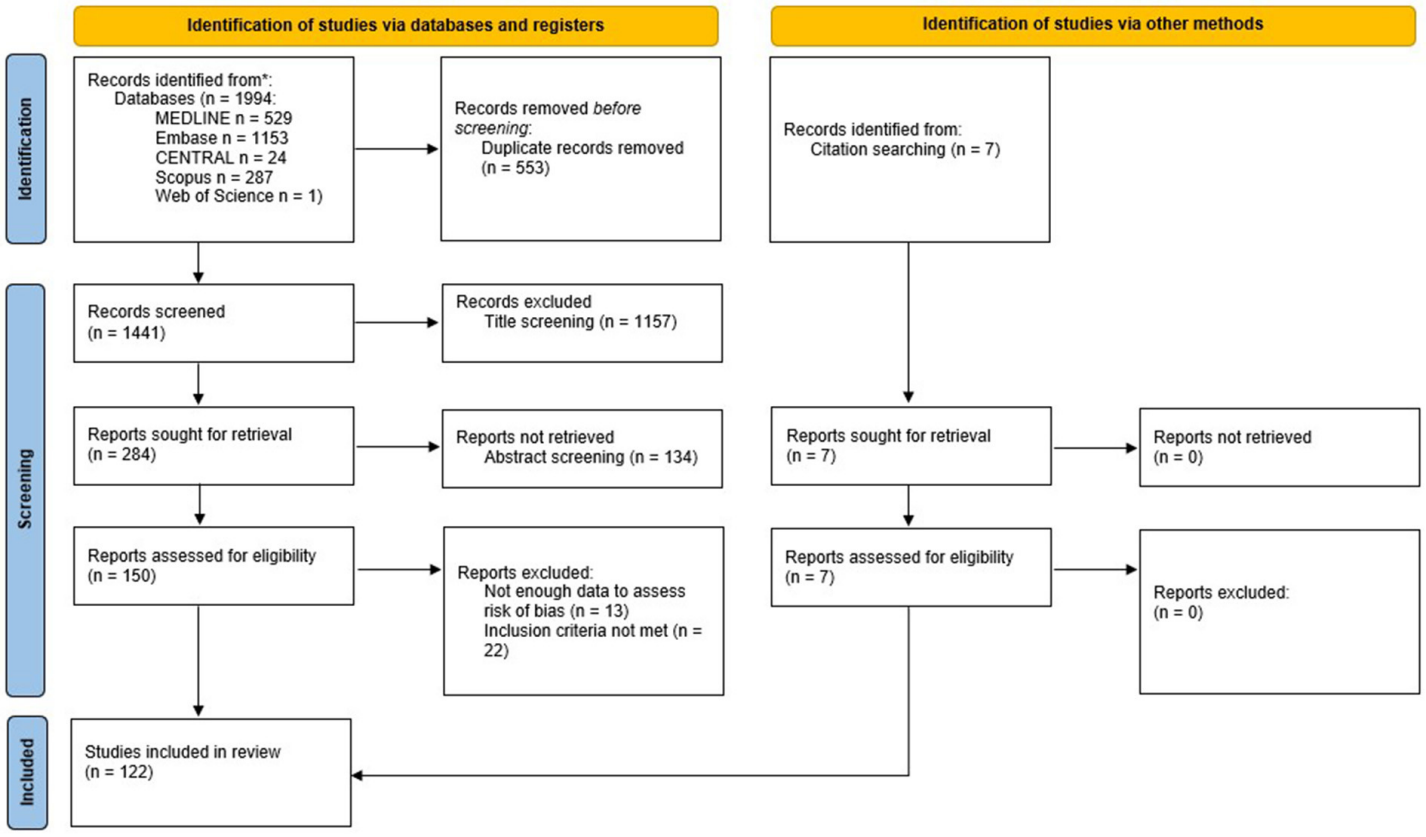
PRISMA flowchart for the systematic review. From Page et al. (2021). For more information, visit: http://www.prisma-statement.org/.

We identified 1,994 records through the initial search. After duplicate removal, 1,441 titled were screened, and 1,157 were excluded. Of the 284 reports sought for retrieval, 134 were not retrieved, because either they represented the same study, the full text was unavailable, or after abstract screening they were deemed not relevant. One hundred and fifty reports were retrieved and assessed for eligibility. Of these, 13 reports were excluded because they did not provide enough data to assess risk of bias, and 22 reports did not meet the other inclusion criteria, or met one of the exclusion criteria. We added other seven reports checking the reference lists of the studies retrieved. Finally, 122 studies were included in this review (the flow diagram is shown in Figure 4).

Where not explicitly described in the text, the design of the cited study should be intended as observational. A list of the relevant data of each study and their risk of bias is presented in the tables within the appropriate sections of the text. Some studies have not been described in the main text, since their less rigorous design or conclusion were thought to be of reduced value to the reader. However, they may be found in section 4 of the Supplementary material, under the respective sub-section as in the following discussion.

## Discussion

### Factors related to the cholinergic system

#### Indicators of cholinergic deficit

Correlates of more advanced cholinergic deficit include clinical signs (such as odor identification impairment), neuropsychiatric syndromes linked to cholinergic deficit (including attention impairment, anxiety, confusion with hallucinations), peripheral signs, imaging predictors, and possible neurophysiological indicators of reduced cholinergic binding. The included studies are summarized in Table 2.

**Table 2:** Indicators of cholinergic deficit.

**Reference**	**Sample**	**Treatment**	**Diagnosis criteria**	**Response criteria**	**Follow-up**	**Predictor**	**Risk of bias**
Devanand et al. (2017)	37 MCI	Donepezil (galantamine or rivastigmine if not tolerated)	Clinical (aMCI with subjective memory complaints, score >1.5 SD below norms on either WMS-III Logical Memory subtest immediate or delayed recall, or the Free and Cued Selective Reminding Test immediate or delayed recall, no functional impairment consistent with dementia, MMSE 23+, CDR 0.5)	ADAS-Cog and Selective Reminding Test change	52 weeks	Decrease in UPSIT score (odor identification test scores) with atropine challenge	Low
Motter et al. (2021)	21 AD	Donepezil	Clinical (probable AD, NINCDS-ADRDA, and NIA)	Change in ADAS-Cog and SRT	52 weeks	Decline in UPSIT after 8 weeks on D is a predictor of poor response; change in UPSIT after atropine challenge is not a predictor	Low
Pelton et al. (2016)	18 depressed	Donepezil	Clinical (subjective cognitive complaints, cognitive impairment ≤ 10 years by history, MMSE >20, major depressive disorder or dysthymic disorder by DSM-IV, HRDS-24 ≤ 14)—no dementia	Change in SRT-tot	12 weeks	Low UPSIT scores in depressed patients with cognitive impairment	Some concerns—moderate
Connelly et al. (2019)	24 AD	Donepezil, galantamine, rivastigmine	Clinical (probable AD, NINCDS-ADRDA, and ICD-10)	Change in MMSE, NOSGER subscales, DSST, and global assessment of change including carer/family views	6 months	Baseline decay to 50% of peak value in recovery from vasodilation associated with acetylcholine iontophoresis ≤ 242 s	High
Sayer et al. (2004)	36 AD	Donepezil	Clinical (probable AD, NINCDS-ADRDA)	At least two of gain of 2+ points on MMSE, improvement of the combined score IALD and social behavior subscales of NOSGER (or maintenance of maximum score), positive global change as defined as a tripartite agreement amongst doctor, subject, and carer (global impression)	6 months	Lower AChE salivary activity is a predictor of poor response	High
Pakrasi et al. (2003)	150 demented	Donepezil, galantamine, rivastigmine	Clinical (dementia, ICD-10 and AD, NINCDS-ADRDA—doesn't state if possible included; DLB, McKeith)	Clinical response: improvement in global clinical condition. Cognitive response: 2+ points improvement on MMSE	3–4 months	Hallucinations	High
McKeith et al. (2004)	120 AD	Rivastigmine	Clinical (probable DLB, McKeith)	Power of attention (sum of CDR items: simple reaction time, choice reaction time, and digit vigilance speed) and quality of memory (sum of numeric working memory, spatial working memory, immediate word recognition, delayed word recognition, and picture recognition sensitivity indices)	20 weeks	Hallucinations are a predictor of response on attentional function	Moderate
Onofrj et al. (2003)	23 demented	Donepezil	Clinical (dementia, DSM-IV, and ICD-10)	Homogeneous improvement in MMSE, ADAS-Cog, and NPI	6 months	EEG P3 latency (for response at 1 month) and CRT variability (for response at 6 months)	High
Ouchi et al. (2015)	15 AD	Donepezil	Clinical (probable AD, NINCDS-ADRDA)	3+ points increase in MMSE	3 months	Higher scores on modified vigilance test from MoCA, lower score on ASA (auditory selective attention) test	High
Meng et al. (2018)	59 early MCI, 48 late MCI, 34 AD, 34 HC	Not clearly stated	ADNI database: MCI (subjective memory concern, abnormal memory function score on the Logical Memory II subscale, MMSE 24–30, CDR ≥ 0.5 with memory box score 0.5+, general cognition and functional performance sufficiently preserved, stability of Permitted Medications for at least 4 weeks prior to screening) and AD (subjective memory concern, abnormal memory function score on the Logical Memory II subscale, MMSE 20–26, CDR = 0.5 or 1.0, NINCDS-ADRDA probable AD)—Appendix E1	Change in ADAS-Cog	6 months	Reduced resting state nucleus basalis of Meynert functional connectivity with left lateral occipital cortex (lateral cholinergic pathway) and left middle frontal gyrus (medial cholinergic pathway) in MCI patients	High
Hanyu et al. (2002)	72 AD	Donepezil (only 5 mg)	Clinical (probable AD, NINCDS-ADRDA)	4+ points improvement on MMSE	14–18 weeks	More severe atrophy of substantia innominata (no cut-off provided)	Moderate
Tanaka et al. (2003)	82 AD	Donepezil (only 5 mg)	Clinical (probable AD, NINCDS-ADRDA)	4+ points improvement on MMSE	12 months	More severe atrophy of substantia innominata (a cut-off of 1.81 distinguishes continuously responding and trasiently responding patients at 3–12 months from non-responders)	Moderate
Kanetaka et al. (2008)	91 AD	Donepezil (only 5 mg)	Clinical (probable AD, NINCDS-ADRDA)	4+ points increase in MMSE	Up to 18 weeks	More severe atrophy of substantia innominata and less prominent hypoperfusion in the frontal lobe; MRI × SPECT index <1.54	Moderate
Teipel et al. (2016)	215 suspected prodromal AD	Donepezil	Clinical (progressive hippocampal amnestic syndrome: free recall ≤ 17 or total recall <40 on FCSRT)	Change in ADAS-Cog-MCI, CDR, TMT-A/B, CVLT	12 + 6 months OLE	Basal forebrain volume is not a predictor	Low
Brown et al. (2003)	20 AD	Donepezil	Clinical (DSM-IV)	Improvement of 2+ points in MMSE or 4+ in ADAS-Cog and improvement on 3+ items of the Informant Questionnaire for change (ADL)	3 months	Greater mAChR deficit ((R,R)[123I]I-QNB SPECT)	Moderate
Jessen et al. (2006)	17 AD	Donepezil	Clinical (probable AD, NINCDS-ADRDA)	Change in ADAS-Cog	12 weeks	Low parietal NAA/Cr ratio at baseline	Moderate
Di Lazzaro et al. (2005)	20 AD and 12 HC	Rivastigmine	Clinical (probable AD, NINCDS-ADRDA)	Stabilization or decrease of GDS	1 year	Abnormal baseline SAI (short afferent inhibition of MEPs) in conjunction with a large increase in SAI after a single dose of rivastigmine	Moderate
Tsai et al. (2015)	17 AD	Donepezil (only 5 mg)	Clinical (probable AD, NINCDS-ADRDA and DSM-IV)	No deterioration in MMSE	12 months	Slope (by least square method) of average multiscale entropy of EEG signal for scales 6–20 over −0.024	Moderate

Three studies evaluated the performances on the University of Pennsylvania Smell Identification Test (UPSIT) a measure of odor identification impairment which is thought to reflect cholinergic impairment due to the early neurofibrillary pathology in the olfactory bulb in AD. The study by Devanand showed that baseline UPSIT was not associated with response at 1 year in MCI patients, whereas a decrease in UPSIT after atropine challenge, theoretically expected in patients with more advanced cholinergic impairment, correlated with greater improvement on the Selective Reminding Test—total recall (a 1-point decrease in UPSIT corresponded to a 0.58 points improvement in SRT-tot), CIBIC-plus and executive function, but not on ADAS-Cog. In patients with a UPSIT score above 14 (which would exclude subjects with congenital anosmia), the authors found that an improvement in UPSIT from baseline to week 8 was associated with a trend of improvement on ADAS-Cog at 26 and 52 weeks (Devanand et al., 2017). Complementarily, the study by Motter found that a decline in UPSIT after 8 weeks on donepezil, which would probably imply a progression in the cholinergic deficit despite treatment, was a predictor of poor cognitive and response at 1 year in AD patients. However, change in UPSIT after atropine challenge was not associated with AChEI benefit, contrarily to the previously cited study. The authors hypothesized that this lack of association was due to the fact that atropine, administered through nasal spray, might have not reached the olfactory bulb, but the small sample may have also contributed to this negative finding (Motter et al., 2021). Although it did not include patients with neurodegenerative dementias, a small RCT by Pelton suggested that a lower UPSIT score could predict cognitive response to donepezil at 3 months in depressed patients with cognitive impairment, reinforcing the hypothesis that AChEI therapy would be generally beneficial in patients with hallmarks of cholinergic deficit, irrespective of the diagnosis (Pelton et al., 2016). It is difficult to draw conclusions from these studies, given the heterogeneity of the populations included and the inconsistent results. Further and larger studies should clarify whether UPSIT could be an efficient tool to predict response to AChEI.

A study by Connelly evaluated the peripheral vasodilatory response to acetylcholine iontophoresis as a possible predictor of AChEI response at 6 months. A cholinergic deficit would imply higher levels of endogenous cholinesterase, and a faster recovery to 50% of peak values of blood flow in response to acetylcholine was found to be predictive of subsequent response to AChEI. A cut-off of 242 s differentiated between responders and non-responders with 90% sensitivity and 77% specificity, with a negative predictive value of 90%. Therefore, the time to recover to 50% of peak values of blood flow after acetylcholine iontophoresis would represent a valuable tool to detect patients for which AChEI therapy would be not beneficial (Connelly et al., 2019). Another interesting aspect of the study is that AChEI responders showed an increase in recovery time at 6 months compared to baseline (possibly indicating an actual inhibition of cholinesterase activity), while the opposite was true for non-responders, probably representing an increased activity of cholinesterase despite AChEI therapy.

A study by Sayer found that probable AD patients who did not respond to donepezil at 6 months exhibited lower salivary AChE activity, supposedly in parallel with a reduced brain cholinergic activity, compared to both AD responders and healthy age-matched controls (Sayer et al., 2004). Although the finding seems interesting, the study has several crucial limitations. The timing of the salivary AChE sampling was not clearly stated, but it was probably coincident with response assessment (which would make salivary AChE a marker of response, not a predictor). Moreover, donepezil 5 mg was withdrawn after 3 months if the patient's MMSE deteriorated and raised to 10 mg if the patient's MMSE did not improve. Finally, the response definition had important elements of subjectivity.

A retrospective study by Pakrasi found hallucinations to be predictive of cognitive response at 3 months, together with a diagnosis of DLB or PDD. Hallucinations in DLB have been considered to be a hallmark of the typical cholinergic deficit of the disease [even though they might also be related to a cortical dopaminergic dysfunction, which would explain the known sensitivity to neuroleptics (McKeith et al., 2004)], since they are associated with both decreased ChAT activity and reduced nicotinic receptors. Moreover, hallucinations would arise from the impaired alertness resulting from choline deficiency (Pakrasi et al., 2003). A *post-hoc* analysis of an RCT performed by McKeith showed that rivastigmine had a significantly better effect on attentional tests, but not on memory tests, in the hallucinators group compared to placebo at 12 and 20 weeks. This effect was not present in the non-hallucinators group (McKeith et al., 2004).

A small cross-over study by Onofrj showed that fluctuating cognition in demented patients, irrespective of diagnosis (even though fluctuating cognition might be a hallmark of DLB), was associated with cognitive and psychiatric response to donepezil at 6 months, compared to non-fluctuating cognition (Onofrj et al., 2003). In the same study, prolonged P3 latency, a well-known marker of cholinergic deficit (Meador et al., 1987), and increased variability in the Choice Reaction Time test (a marker of attention impairment, again related to cholinergic deficit) were the best predictors for response at 1 and 6 months, respectively (Onofrj et al., 2003). Taken together, these findings show that neuropsychological and neurophysiological markers of cholinergic deficit could identify a subset of demented patients who respond better to donepezil.

A small study by Ouchi showed that AD patients responding to donepezil at 3 months had higher scores on a modified vigilance test from MoCA and lower scores on an auditory selective attention test (which required patients to respond to a target voice heard among a 60–70 dB noise). Therefore, the authors suggested that responders had higher “simple” auditory attention, but impaired “complex” auditory attention. Moreover, a subset of the responders underwent PET scans with a donepezil tracer, showing higher cholinesterase activity. Thus, the performance exhibited by responders in the two tests could be the expression of higher cholinergic deficit (Ouchi et al., 2015).

A study by Meng on subjects from the ADNI database found that reduced resting state NB connectivity with the left occipital cortex (lateral cholinergic pathway) and the left middle frontal gyrus (withing the medial cholinergic pathway) was predictive of cognitive response at 6 months in MCI patients. It was not possible to establish whether AChEI had the same effect in AD patients, since AD subjects in the ADNI database were already on treatment when they were enrolled (Meng et al., 2018).

Two studies by Hanyu and Tanaka found that more severe atrophy of the substantia innominata (measured on coronal T2-weighted images at the plane through the anterior commissure), theoretically implying a more severe cholinergic depletion, was a predictor of cognitive response to donepezil in AD patients at 3 months and 1 year (even though the magnitude of correlation was greater at 3 months). A cut-off value of 1.81 mm of substantia innominata thickness distinguished responders from non-responders with 71% sensitivity and 69% specificity in the latter study (Hanyu et al., 2002; Tanaka et al., 2003). Kanetaka et al. found that the combination of more severe atrophy of substantia innominata and less prominent frontal hypoperfusion into an MRI × SPECT index (multiplying the thickness of the substantia innominate to the z-score of perfusion in the frontal lobe) was even more accurate in individuating responders. A cut-off of 1.54 achieved 71% sensitivity and 70% specificity for cognitive response to donepezil at 3 months (Kanetaka et al., 2008). An analysis of a RCT by Teipel found that basal forebrain volume was not predictive of cognitive or global response to donepezil in a suspected prodromal AD cohort (Teipel et al., 2016).

A small study by Brown found that lower (R,R)[123I]I-quinuclidinyl benzilate (R,R[123I]I-QNB) SPET binding in the bilateral insula (especially on the left) was predictive of response to donepezil at 3 months, and negatively correlated with ADAS-Cog change (i.e., the lower the binding, the greater the improvement). Since (R,R)[123I]I-QNB is a tracer of M1/M4 muscarinic receptors, the authors suggested that these findings would imply a more marked cholinergic deficit in responders. They also speculated that the insular cortex involvement might be due to its role as a limbic integration area, with an important network of AChE-rich fibers (Brown et al., 2003).

A small study by Jessen found that a lower baseline NAA/Cr ratio in the parietal lobe (but not in the medial temporal lobe) was predictive of cognitive response to donepezil at 3 months. The authors report that NAA receives its acetyl group from acetyl-CoA in neuronal mitochondria, and thus a cholinergic deficit in the cortex, resulting in a displacement of acetyl-CoA from the mitochondria to the cytoplasm, would lead to a reduction of NAA synthesis (Jessen et al., 2006). However, the reasons behind the lack of association of NAA/Cr ratio in the medial temporal lobe and cognitive response to donepezil are not clear. Since NAA is considered a neuronal marker (and thus a decreased NAA/Cr ratio suggests neuronal loss), a possible explanation could be found in the loss of neurons that are needed to produce such a response.

A study by Di Lazzaro investigated the utility of short latency afferent inhibition (SAI) of the motor evoked potentials following an afferent stimulus at the level of the median nerve at the wrist. Short latency afferent inhibition was obtained by averaging the percentage of inhibition of the conditioned motor evoked potentials (compared to the responses induced by transcranial magnetic stimulation alone) across seven different interstimulus intervals, and it has been shown to be at least partially dependent on cholinergic transmission within the brain. Thus, a decreased SAI would reflect a greater cholinergic deficit. Coherently, SAI was larger in controls than in AD patients. The authors found that an increase in SAI > 8% of test size (two standard deviations above the mean of control subjects, which reflected the test intrinsic variability) after a single dose of rivastigmine was predictive of global and cognitive response at 1 year, together with abnormal baseline SAI (Di Lazzaro et al., 2005). This would imply that only patients with a cholinergic deficit restored by rivastigmine were subsequently able to respond to the drug.

A study by Tsai used multiscale entropy (MSE) in the analysis of EEG recordings to predict cognitive response to donepezil in AD patients at 1 year. Briefly, MSE analysis consists of coarse-graining the digitalized signals into different time scales, quantifying the degree of irregularity in each time series using sample entropy. Higher fluctuations in data correspond to higher entropy, with a more negative slope of MSE and higher complexity of the signal. Operatively, the original series are segregated into blocks, with each block containing *n* data points. The mean signal value over each block forms the coarse-grained time series at scale *n*, so for instance at scale 2 the signal is the series obtained averaging two consecutive data points (Sapien Labs, 2018). The authors found that the baseline slope of average MSE over all leads for time scales 6–20 over −0.0124 had a 85.7% sensitivity and 60% specificity for cognitive response. They report that the slope of the mean value of MSE over scales 1–5 could be associated with ACh transfer time from the presynapse to postsynapse (which takes approximately 15 ms). Thus, this slope could be sensitive to either the amount of released ACh or the synaptic concentration of ACh, while the one over scales 6–20 might be associated with the binding time of acetylcholine (scale 6 corresponds to approximately 20 ms in the time scales, which is the time it takes to break the acetylcholine molecules from the binding receptor sites). Therefore, the finding of a less negative slope of MSE over time series 6–20 would reflect a loss of signal complexity due to lower acetylcholine molecular binding ability or binding count (Tsai et al., 2015). In the end, this would represent another correlate of greater cholinergic deficit.

#### Indicators of preserved cholinergic system

Most studies utilized functional or structural neuroimaging to estimate the preservation of the cholinergic pathways in relation to response to AChEI, while a few studies used EEG. In general, the majority of the included studies support the notion that the preservation of the cholinergic fibers (in the context of reduced acetylcholine, i.e., a true “neurotransmitter” impairment, with “anatomical” preservation) is related to a better cognitive response to AChEI. The results of the included studies are summarized in Table 3.

**Table 3:** Indicators of preserved cholinergic system.

**Reference**	**Population**	**Treatment**	**Diagnosis**	**Response criteria**	**Follow-up**	**Predictor**	**Risk of bias**
Yoshida et al. (2007)	29 AD patients	Donepezil 5 mg	Clinical (probable AD, NINCDS-ADRDA, and DSM-IV and ICD-10)	3+ points improvement in ADAS-Cog (not clear at which time-point they made the analyses); response at 1 year seems to have been defined as a non-deterioration of ADAS-Cog	1 year	Increase in blood flow in anterior frontal lobe and parietal lobe after 1 month (for response at 1–3 months); reduction of blood flow in all regions after 1 month is a predictor of poor response	Moderate
Fukui and Taguchi (2005)	55 AD patients (50 completed the studies)	Donepezil 5 mg	Clinical (probable AD, ICD-10, and NINCDS-ADRDA)	Improvement of 2+ points on the Clock-drawing test (Marucci et al., 2020 for scoring) for true responders, ±2 points on the CDT for unchanged	12 months	White matter hyperintensities are not a predictor	Moderate
Modrego et al. (2009)	54 AD patients (50 included, 43 stayed on G)	Galantamine (but switching to donepezil allowed)	Clinical (probable AD, NINCDS-ADRDA)	Changes in ADAS-Cog, NPI, and DAD, stroke, death	6 months	Microinfarctions and leukoaraiosis are not predictors	Low
Cheng et al. (2015)	353 AD patients	Donepezil, Rivastigmine, Galantamine	Clinical (probable AD, NINCDS-ADRDA 1984)	Longer TMSE decline-free survival (Survival analysis, using TMSE decline of 3+ points as endpoint)	Median 46.6 months	White matter hyperintensities are not a predictor	Low
Hongo et al. (2008)	41 AD patients	Donepezil 5 mg	Clinical (probable AD, NINCDS-ADRDA)	Change in ADAS-Cog and MMSE	24 weeks	Lower rCBF values in right orbito-frontal cortex; subcortical hyperintensities in the cholinergic pathways are not a predictor	Low
Ho et al. (2016)	87 AD patients	Rivastigmine	Not clearly stated (presumably probable AD, NINCDS-ADRDA)	No deterioration on MMSE or CDR-SB	1 year	Severe periventricular and deep white matter changes	Moderate
Wu et al. (2018)	196 AD patients	Donepezil	Clinical (probable AD, NINCDS-ADRDA)	No deterioration in CASI	1 year	White matter changes in frontal areas and left basal ganglia are predictors of poor response	Low
Kasuya et al. (2012)	80 AD patients	Donepezil 5 mg	Clinical (probable AD, NINCDS-ADRDA)	CGI score of 2 and 3+ points increase in MMSE	6 months	Higher distribution volume (ratio of [11C]-donepezil in cerebral tissue to plasma—so higher acetylcholinesterase levels in the brain, or donepezil-binding sites)	High
Babiloni et al. (2006)	58 AD patients	Donepezil	Clinical (probable AD, NINCDS-ADRDA, and DSM-IV)	No deterioration on MMSE	11.3 ± 2.6 months	Weaker magnitude of diffuse cortical sources of delta (F-P-O-T), and posterior alpha 1 (O-T) and alpha 2 (O) frequencies on LORETA (Low-resolution brain electromagnetic tomography)	Moderate
Dos Santos Moraes et al. (2006)	35 AD patients	Donepezil 5 mg	Clinical (probable AD, ADRDA)	Cognitive improvement rate in ADAS-Cog ([baseline – 6 months]/baseline)	6 months	Baseline overall, frontal, and parietal alpha EEG power	Low

A study by Yoshida found that cerebral blood flow change after donepezil treatment at 1 month was able to differentiate cognitive responders and non-responders at 1 year. In particular, compared to pre-treatment values, blood flow was significantly decreased in all regions in non-responders, while in responders blood flow increased in the anterior frontal lobe and parietal lobe. Blood flow in the putamen was significantly higher in responders at 1 and 3 months compared to non-responders, and at 3 months, blood flow in the thalamus was significantly higher in responders. Since in responders the regions where blood flow was maintained were found to be in the main cholinergic pathways, it could be suggested that this subgroup may consist of a subpopulation with preserved cholinergic neurons. The increased cerebral blood flow in the basal ganglia and the thalamus may result from both an increase in the activity of the cholinergic neurons in the lateral tegmental field (which project into the former subcortical structures) and an indirect effect of the increased blood flow in the frontal lobe (Yoshida et al., 2007).

Most studies did not find an association between white matter lesions and response. A paper by Fukui reported that white matter hyperintensities and basal ganglia lesions (measured on T2-weighted and FLAIR images with the Fazekas scale) were not predictive of response to donepezil at 3 months, while periventricular hyperintensities were associated with an improvement in the Clock Drawing test, even though the finding was dependent on age and high blood pressure. Thus, the actual conclusion would be that white matter hyperintensities are not a predictor of response, neither in the cholinergic pathways, nor outside them (Fukui and Taguchi, 2005). A study by Modrego found that microinfarctions and leukoaraiosis, detected on T2 or FLAIR images using the Wahlund scale, were not predictive of clinical response to galantamine (Modrego et al., 2009), and a study by Cheng found that white matter hyperintensities (measured with a modification of the Fazekas scale on CT or FLAIR images) did not predict cognitive response to all AChEI (Cheng et al., 2015). A study by Hongo found that subcortical hyperintensities in the cholinergic pathways on axial T2-weighted images were not associated with cognitive response to donepezil (Hongo et al., 2008).

A study by Ho found that severe deep and periventricular white matter lesions were predictive of cognitive (but not global) response to rivastigmine at 1 year in Taiwanese AD patients, which the authors believe to be related to a more pronounced cholinergic deficit. White matter lesions were assessed on MRI images using a modified version of the Fazekas scale, but the authors do not provide further details on the sequences used (Ho et al., 2016). Conversely, a retrospective study by Wu found that white matter changes in the frontal areas and the basal ganglia, evaluated on CT scans with the Age-Related White Matter Changes, were predictive of poor response to donepezil at 1 year. The author related this finding to the disruption of medial and lateral cholinergic pathways which originate from the basal forebrain, as well as to a reduction of the subcortical AChE due to the disruption of AchE-rich fibers (Wu et al., 2018). More studies are needed to clarify these contradictory findings, which may partially arise from the different scoring methods used.

A study by Kasuya showed that a higher distribution volume of [11C]-donepezil on brain PET scans at baseline, which is thought to reflect a higher AChE level or activity, is predictive of response to donepezil at 6 months. The authors speculate that this could be a marker of preserved cholinergic neurons, since post-synaptic AChE may be downregulated as a compensatory mechanism after cholinergic neurons loss (Kasuya et al., 2012).

A study by Babiloni used low-resolution brain electromagnetic tomography to distinguish cognitive responders to donepezil from non-responders at 1 year. This is an EEG reference-free analysis technique, consisting of voxel current density values which are able to predict EEG spectral power density at scalp electrodes. The authors found that non-responders had a stronger magnitude of frontal, parietal, occipital, and temporal delta, occipital and temporal alpha 1, and occipital alpha 2 sources compared to responders. Moreover, the stronger the frontal and temporal delta sources were, the lower was the cognitive efficacy of donepezil. The authors suggested that these findings would reflect an underlying disruption of cholinergic inputs from the NB to the hippocampus, the cortex, and the thalamocortical projections, which normally would lower delta and sustain alpha in awake conditions, enabling optimal cortical information processing. In non-responders, an impairment of the NB would alter the reciprocal inhibition of delta and alpha, thus explaining the stronger delta source. The higher alpha source might be explained by the fact that an impaired cholinergic projections activity would cause disinhibition of corticofugal slow oscillations, which in turn would trigger thalamic-generated rhythms (at 1–4 Hz) and spindle rhythms (7–14 Hz). The latter overlap in frequency with alpha rhythms, thus increasing the magnitude of alpha sources. The inverse correlation between fronto-temporal delta and change in MMSE would imply that donepezil treatment might fail in patients with a more pronounced loss of cholinergic neurons in frontal and temporal cortical areas (Babiloni et al., 2006).

A RCT by dos Santos Moraes found that baseline REM sleep EEG overall, frontal, and centroparietal absolute alpha power was associated with cognitive response to donepezil at 6 months. The authors speculate that faster REM sleep EEG frequencies in centroparietal areas would reflect cholinergic system preservation, since ACh is greatly involved in cortical desynchronization during this stage of sleep. Consistently, donepezil was found to reduce slow frequencies of REM sleep EEG (decreasing frontal theta band absolute power), which are dependent on a decreased cholinergic input (Dos Santos Moraes et al., 2006).

#### BuChE

The missense polymorphism rs1803274, the so-called BuChE-K variant, leads to a reduced BuChE activity [although maybe not in the brain (Patterson et al., 2011)], and it is among the most extensively studied SNPs in relation to AChEI response, with rather conflicting results. However, the effect of several other SNPs has been studied. BuChE-K seems to be less efficient in stopping accumulation of Aβ into fibrils *in vitro*, which may increase the risk of AD (Patterson et al., 2011). Thus, the effect of BuChE-K could be detrimental in the earlier phases of diseases, when it might cause a higher amyloid-induced degeneration, but protective in late stages, when it may preserve brain ACh by means of reduced degradation. If that hypothesis will be confirmed by further studies, it could be intriguing to investigate whether AChEI treatment (at least with drugs that are active on BuChE) could be efficiently delayed according to BuChE genotype.

A study by Scacchi failed to find any influence at all of BuChE-K variant or rs1355534 SNP on cognitive response to donepezil or rivastigmine in Italian late-onset AD patients (Scacchi et al., 2009). A study by Chianella extended this negative finding to all AChEI (Chianella et al., 2011).

A retrospective analysis of a RCT by Sokolow found that amnesic MCI patients—thus in the earlier phases of disease—who were BuChE-K variant carriers had worse responses to donepezil as measured by MMSE and CDR-SB at 3 years, especially if they were concomitantly carrying APOE-ε4. The authors thought that this was due to a deleterious overload of ACh resulting from reduced BuChE activity (which might be furtherly reduced in the brain of APOE-ε4 carriers) and donepezil therapy (Sokolow et al., 2017).

Conversely, another analysis of the same RCT by De Beaumont found that women with amnesic MCI carrying the BuChE-K variant exhibited better cognitive responses (measured by ADAS-Cog) to donepezil at 3 years, whereas men did not. It is interesting to notice that patients carrying both BuChE-K variant and APOE-ε4 had an earlier onset of cognitive impairment. The authors speculated that the lack of estrogen in post-menopausal women, which negatively affects basal forebrain function, could lead to a more extensive cholinergic deficit in the already more compromised sample of BuChE-K carriers (De Beaumont et al., 2016). A study by Patterson found that BuChE-K variant was predictive of cognitive response at 2 years only in patients with baseline MMSE under 16, thus supporting the idea that BuChE-K might be beneficial in late stages of disease (Patterson et al., 2011).

#### CHAT

A study by Harold found that AD patients carrying rs733722 SNP of CHAT TT alleles had better cognitive response to AChEI (Harold et al., 2006). The rs733722 SNP lies in the promotor region of CHAT; however, a recent paper did not find any differential effect of TT alleles compared to G allele in terms of ChAT activity or protein concentration (Rocha-dias et al., 2020). Conversely, AD patients carrying the G allele on rs1880676, while exhibiting lower ChAT activity and concentration, did not show differences in terms of cognitive response to AChEI compared to non-carriers (Harold et al., 2006; Rocha-dias et al., 2020). Thus, an explanation for these findings does not seem exclusively related to the cholinergic deficit.

A study by Yoon found rs2177370 C allele and rs3793790 A allele (both located in the same intron) to be associated with cognitive response at 26 weeks in South Korean AD patients. Moreover, haplotype CT of rs11101187–rs2177370 of CHAT was found to be associated with cognitive response, while haplotype CC was a predictor of poor response. The authors suggest that the latter haplotype might be associated with reduced ACh synthesis, but this hypothesis needs confirmation (Yoon et al., 2015).

#### CHRNA7

A study by Braga found that the T allele of rs6494223 SNP of CHRNA7 was predictive of cognitive response at 6 months (but the association was lost at 2 years). However, the effect was significant only in patients with baseline MMSE over 20. The authors report that the presence of the T allele is related to a higher cholinergic deficit. They also report that the same SNP is related to a reduced probability of AD progression (Braga et al., 2015).

A retrospective study by Weng found that the G allele of rs8024987 of CHRNA7 was predictive of cognitive response at 6 months in women (especially in those taking galantamine). More specifically, women who were G/G homozygotes on rs8024987 and rs885071, in particular those on galantamine, were more likely to be responders (Weng et al., 2013).

A study by Clarelli did not replicate the findings by Braga and Weng in an Italian cohort at 1 year (not even after pooling data together with the study by Braga). Moreover, their study did not support the involvement of any SNP of CHRNA7 in response to AChEI (Clarelli et al., 2016). Thus, more studies are needed to elucidate the role of CHRNA7 in cognitive response to AChEI and its hypothesized role in neuroprotection.

#### Acetylcholinesterase gene

A study by Scacchi found that the A/A genotype of ACHE rs2571598 SNP was associated with cognitive response to rivastigmine (but not to donepezil) in Italian late-onset AD patients. This genotype has been found to result in lower AChE activity and higher levels of acetylcholine in other works, which may explain its association with response. However, the reason why this positive effect on cognition was present only in patients taking rivastigmine still needs to be elucidated (Scacchi et al., 2009).

A study by Yoon failed to find any haplotype associated with response in ACHE, but they did not analyze the SNP individuated by Scacchi (Yoon et al., 2015).

#### Paraoxonase-1

A study by Pola found that Italian AD patients carrying the R allele on residue 192 of PON-1 had a better cognitive response to donepezil and rivastigmine, while the QQ genotype was a predictor of poor response. The R allele is associated with a higher activity of PON-1, which catalyzes the hydrolysis of exogenous AChEI, such as different organophosphates. The authors argued that an enhanced activity of PON-1 could act synergically with pharmacological AChEI to determine cognitive responses, but this is based on their inaccurate claim that PON-1 is an endogenous AChEI (Pola et al., 2005).

Conversely, a study by Klimkowicz-Mrowiec found that neither the SNP on residue 192 nor the SNPs on residues 55 and 161 were associated with response to donepezil and rivastigmine in a Polish AD cohort (Klimkowicz-Mrowiec et al., 2011). Further studies are needed to elucidate whether PON-1 could be useful in predicting response to AChEI or not. The results of the studies on the genetics of the cholinergic system are summarized in Supplementary Table S1.

### Factors related to the drug

#### Factors involved in drug metabolization

CYP2D6 genotype has been extensively studied, but a few considerations need to be made before analyzing the findings of the literature. Firstly, CYP2D6 is only relevant for donepezil and galantamine therapy. Secondly, certain factors could modulate the role of this cytochrome, such as concomitant use of memantine [which inhibits CYP2D6 and raises donepezil concentrations (Yaowaluk et al., 2019)] and APOE [with ε4 genotype seemingly able to convert CYP2D6 extensive metabolizers into full poor metabolizers (Liu et al., 2014)]. Lastly, it is important to notice that results may be affected by ethnicity and the population studied (Miranda et al., 2017). For instance, 37.9% of the Chinese population carries the CYP2D6^*^1 variant, which enhances CYP2D6 activity, and 51.3% carries the CYP2D6^*^10 variant on rs1065852 SNP, which reduces CYP2D6 activity (Lu et al., 2016; Ma et al., 2019), whereas CYP2D6^*^3, CYP2D6^*^4, and CYP2D6^*^5, which account for 98% of poor metabolizers in Caucasians, only occur in negligible percentages in Chinese patients (Ma et al., 2019). In two different studies, Lu et al. found that response to donepezil was better in patients with CYP2D6^*^10/10 alleles on rs1065852 SNP (especially if APOE-ε3 non-carriers), and worse in patients with CYP2D6^*^1/10 alleles (Lu et al., 2015, 2016). The same finding was confirmed in a study by Ma, which used patients on rivastigmine (the metabolism of which does not pass through CYP2D6) as control group. In the same study, patients carrying the CYP2D6^*^10 allele had significantly fewer adverse effects to donepezil and galantamine (Ma et al., 2019). A study by Zhong found that patients with either CYP2D6^*^1/10 or CYP2D6^*^10/10 genotypes had better cognitive responses and higher plasmatic concentration of donepezil at 6 months compared to subjects with CYP2D6^*^1/1; however, there was no significant difference in donepezil concentration between responders and non-responders (Zhong et al., 2013). The studies on CYP2D6 are summarized in Supplementary Table S2.

A study by Magliulo showed that heterozygous CYP2D6^*^1 Italian AD patients had better cognitive responses to donepezil compared to homozygous CYP2D6^*^1 (Magliulo et al., 2011).

A study by Seripa found that variants of CYP2D6 associated with decreased or absent enzymatic activity (namely, CYP2D6^*^41, CYP2D6^*^9, CYP2D6^*^29, CYP2D6^*^3, CYP2D6^*^4, CYP2D6^*^7, CYP2D6^*^8, CYP2D6^*^14, CYP2D6^*^6, CYP2D6^*^17, CYP2D6^*^12, and CYP2D6^*^10, as well as gene deletion—CYP2D6^*^5) were associated with response to donepezil at 6 months in Italian AD patients (Seripa et al., 2011).

Conversely, two studies by Miranda found that CYP2D6 status was not predictive of cognitive response to donepezil at 1 year in Brazilian AD and mixed dementia patients (Miranda et al., 2015, 2017). This was confirmed by Chianella in Italian AD patients (Chianella et al., 2011).

A study by Pilotto found that G allele on rs1080895 SNP (CYP2D6^*^2A) was associated with cognitive non-response to donepezil at 6 months in Italian AD patients. The risk of being a non-responder increased with the number of G alleles, reaching an OR of 15.8 in G/G homozygotes (Pilotto et al., 2009). Albani et al. did not replicate this association, but grouping their data with those with the study by Pilotto the authors found that G-allele carriers had a higher probability of being cognitive non-responders to donepezil at 6 months (especially if they also carried APOE-ε4 allele) (Albani et al., 2012). Since this SNP does not seem to influence CYP2D6 enzymatic activity, it is possible that it could be in linkage disequilibrium with a yet to be identified locus with an actual functional meaning.

Conversely, a retrospective study by Chou found that Taiwanese G/G homozygotes had an increased probability of globally responding to donepezil at 2 years. However, as for the rs1065852 SNP, the genotype frequency of CYP2D6 rs1080985 in their cohort was different from the one reported in Caucasians and Chinese populations (Chou et al., 2021). Interestingly, they found that G/G homozygotes had the highest concentration-to-dose ratio of donepezil, even though that result did not reach statistical significance. Different ethnicity-related factors may contribute to these contradictory findings in different populations. Notably, a study by Liu found no influence of rs1080985 SNP on response to donepezil at 6 months in Chinese AD patients (Liu et al., 2014).

CYP3A4 is another cytochrome involved in donepezil and galantamine metabolism. In the study by Ma, CYP3A4^*^1G non-carriers were shown to have poorer cognitive response at 1 year, especially in CYP2D6^*^10 non-carriers (Ma et al., 2019). A study by Magliulo failed to find any influence of CYP3A4 or CYP3A5 on cognitive response in Italian AD patients (Magliulo et al., 2011).

#### Serum albumin

Albumin binds to AChEI, reducing their availability. Rozzini et al. evaluated the influences of the serum concentration of albumin on cognitive response to AChEI, finding significant changes in ADAS-Cog in favor of the low (≤ 4 g/dl) vs. high (>4.5 g/dl) albumin level group at 3 and 15 months in AD patients treated with donepezil. No differences were observed between low and medium albumin level groups. In AD patients treated with rivastigmine serum albumin was not a predictor of response at any time point (Rozzini et al., 2008). This could however be expected, knowing that donepezil exhibits a quite high protein binding of 75%, while rivastigmine and galantamine albumin binding is only 40% and 20% (Jann et al., 2002).

#### AChEI plasma concentration

A study by Ortner showed that rivastigmine serum concentrations were significantly and positively associated with cognitive response on the word list delayed recall subtest of the Consortium to Establish a Registry for Alzheimer's Disease Neuropsychological Assessment Battery (CERAD), although the regression model explained only 26.9% of the test variability. There was a lack of concentration-response association for donepezil for any of the CERAD subtests, although donepezil blood levels were influenced by CYP2D6 gene dose and metabolic profile (Ortner et al., 2020). In a small study by Yang, a higher concentration of donepezil was correlated only to an improvement in the long-term memory domain of the Cognitive Abilities Screening Instrument (Yang et al., 2013).

A small study by Lin failed to find any correlation between galantamine concentration and cognitive or global response at 6 months in Taiwanese AD patients treated with galantamine 8 mg (Lin et al., 2019). A study by Miranda found that donepezil concentration was not predictive of cognitive response to donepezil (Miranda et al., 2017).

Taken together, these studies provide at most scarce evidence of a limited association between higher drug concentrations and response (treated as a dichotomous variable). Only one study investigated the relationship between AChEI concentrations and the magnitude of response (treated as a continuous variable), suggesting that a proportional relationship might exist (Ortner et al., 2020); further studies are needed to confirm this finding.

#### Drug dose

Three studies by Wattmo found mean higher doses of AChEI to be related to cognitive and functional response at 6 months and 3 years in a large sample of Swedish AD patients (Wattmo et al., 2011, 2012; Wattmo and Wallin, 2017). A *post-hoc* analysis of a RCT by Farlow found that patients with severe AD on rivastigmine patch 13.3 mg/day exhibited better response on the ADAS-Clinical Global Impression of Change (ADAS-CGIC) at 6 months compared to patients on rivastigmine patch 4.6 mg/day (Farlow et al., 2015). Another more recent *post-hoc* analysis of the OPTIMA study, performed by Molinuevo, found that patients with moderate AD on rivastigmine patch 13.3 mg/day were more likely to exhibit cognitive and functional response at 12 months compared to subjects on rivastigmine patch 9.5 mg/day. However, patients were included in the RCT phase only if they showed cognitive deterioration during the dose titration phase, which restricts the suggested superiority of high-dose rivastigmine only in patients not responding to conventional doses (Molinuevo et al., 2015).

Notably, a study by Raschetti found that a high dosage of AChEI was associated with the risk of developing adverse drug reactions (Raschetti et al., 2005), which could probably be limited through the combination with peripheral blockers (Hampel et al., 2018). To summarize, higher drug doses seem to be related to better responses, although such doses are not licensed in every country, and caution is nevertheless advised due to the risk of adverse events.

#### Drug type

A large study by Raschetti found that rivastigmine and galantamine were associated with a higher risk of developing adverse drug reactions (Raschetti et al., 2005), while an analysis of a RCT failed to show a preferential association between specific drugs and response (Touchon et al., 2006), supporting the notion that the efficacy of the different AChEI is comparable.

All the other studies on factors involved in drug availability are summarized in Table 4.

**Table 4:** Other factors involved in drug availability.

**Reference**	**Population**	**Treatment**	**Diagnosis**	**Response criteria**	**Follow-up**	**Predictor**	**Risk of bias**
Ma et al. (2019)	174 AD patients (with patients treated with R as a control group)	Donepezil, galantamine, rivastigmine	Clinical (probable and possible AD, NINCDS-ADRDA)	No deterioration in ADAS-Cog and MMSE	1 year	CYP3A4*1G carriers have poorer responses (especially if CYP2D6 non-carriers)	High
Magliulo et al. (2011)	54 AD patients	Donepezil	Clinical (probable AD, NINCDS-ADRDA)	Not clearly stated (change in CIBIC-plus or in MMSE)	Up to 40 months (median 9 months)	CYP3A4 and CYP3A5 are not a predictor	High
Rozzini et al. (2008)	110 AD patients (98 completed the study)	Donepezil	Clinical (probable AD, NINCDS-ADRDA)	Change in ADAS-Cog (not clearly stated)	15 months	Albumin <4 g/dl (for donepezil)	High
Ortner et al. (2020)	48 AD patients on D, 28 AD patients on R	Donepezil, rivastigmine	Clinical (AD, NIAA)	Change in CERAD-NAB subtests and total score	Not stated, mean 5.7 ± 2.53 months for D and 5.59 ± 2.09 months for R	Higher rivastigmine concentration; donepezil concentrations are not a predictor	Low
Chen et al. (2017)	63 AD patients	Rivastigmine	Clinical (NINCDS-ADRDA, not stated if probable or possible)	Improvement in either MMSE or CDR	6 months	Rivastigmine concentration	Moderate
Yang et al. (2013)	37 AD patients	Donepezil 5 mg	Clinical (NINCDS-ADRDA, not stated if possible included)	No deterioration in total and subscales of CASI scores	6 months	Higher donepezil plasma concentration (for long-term memory response)	Moderate
Lu et al. (2015)	77 AD patients	Donepezil	Not stated	No deterioration in MMSE	At least 3 months	Higher concentration of S-Donepezil (in patients with CYP2D6*10/10 on rs1065852 SNP)	High
Lin et al. (2019)	33 AD patients	Galantamine 8 mg	Clinical (NINCDS-ADRDA, not stated if possible included)	No deterioration in MMSE, CASI, and CDR-SB (doesn't state if only one is needed)	6 months	Galantamine plasma concentration is not a predictor	Low
Miranda et al. (2017)	42 AD and AD+CVD patients	Donepezil	Clinical (AD, NIAA; AD+CVD, NINDS-AIREN)	Less than 1 point loss on MMSE	12 months	Donepezil concentration is not a predictor	Low
Wattmo et al. (2018)	1,017 AD patients	Donepezil, galantamine, rivastigmine	Clinical (possible or probable AD, NINCDS-ADRDA, and DSM-IV)	No deterioration in MMSE	3 years	Higher mean dose of ChEI	High
Wattmo et al. (2011)	843 AD patients	Donepezil, galantamine, rivastigmine	Clinical (possible or probable AD, NINCDS-ADRDA, and DSM-IV)	Not clearly stated, changes in MMSE and ADAS-Cog	3 years	Higher mean does of ChEI	High
Wattmo et al. (2012)	784 AD patients	Donepezil, galantamine, rivastigmine	Clinical (possible or probable AD, NINCDS-ADRDA, and DSM-IV)	No deterioration in IADL or PSMS at 6 months	3 years	Higher mean does of ChEI	High
Farlow et al. (2015)	716 severe AD patients (628 included)	Rivastigmine 13.3/4.6 mg	Clinical (probable AD, NINCDS-ADRDA)	Improvement/improvement or no change on ADCS-CGIC	24 weeks	Treatment with 13.3 mg/24 h rivastigmine patch	Some concerns—Low
Molinuevo et al. (2015)	536 AD patients (ITT analysis)	Rivastigmine	Clinical (probable AD, NINCDS-ADRDA)	Improvement of 4+ points on ADAS-Cog, with no decline on ADCS-IADL	48 weeks	Treatment with 13.3 mg/24 h rivastigmine patch	Some concerns—Low
Touchon et al. (2006)	994 AD patients (ITT-LOCF)	Donepezil, rivastigmine	Clinical (probable AD, NINCDS-ADRDA, and DSM-IV; DLB with McKeith or concurrent use of anti-parkinsonian medication)	Change in Severe Impairment Battery, GDS, ADCSL-ADL, MMSE, NPI	104 weeks	No predictors	Some concerns—Low

### Other predictors

Several other predictors with less clear neuropathological correlations have been identified. In this section, we will attempt to summarize and group them according to the diagnostic area in which they have been individuated.

#### Indicators of cognitive reserve

Higher education has been identified as a proxy of cognitive reserve, which has been thought to delay the time to clinically detectable impairment. However, this would correspond to a more advanced disease at the time of the diagnosis (Wattmo et al., 2011, Wattmo and Wallin, 2017). The studies included in this section are summarized in Table 5. A retrospective study by Boccardi found that higher education was a predictor of short-term cognitive response, together with higher ADL score at baseline (Boccardi et al., 2017). A study by Chen suggested the same association, together with lower CDR-SB at baseline (Chen et al., 2017).

**Table 5:** Cognitive reserve.

**Reference**	**Population**	**Treatment**	**Diagnosis**	**Response criteria**	**Follow-up**	**Predictor**	**Risk of bias**
Boccardi et al. (2017)	628 AD patients	Donepezil, galantamine, rivastigmine	Not stated	MMSE stabilization or improvement at 3 months	3 years	Higher education (for response at 3 months)	Moderate
Chen et al. (2017)	63 AD patients	R	Clinical (NINCDS-ADRDA, not stated if probable or possible)	Improvement in either MMSE or CDR	6 months	Higher education	Moderate
Gallucci et al. (2016)	84 AD patients + 6 AD+CVD patients	Donepezil, galantamine, rivastigmine	Clinical (NINCDS-ADRDA and NINDS-AIREN, not stated if possible included)	Less than 2 points deterioration of MMSE per year	Up to 4 years	“White collar” (office workers, teachers, professionals), living with assistance, being married	High
Wattmo and Wallin (2017)	1,017 AD patients	Donepezil, galantamine, rivastigmine	Clinical (possible or probable AD, NINCDS-ADRDA, and DSM-IV)	No deterioration in MMSE	3 years	Lower level of education	High
Wattmo et al. (2011)	843 AD patients	Donepezil, rivastigmine, galantamine	Clinical (possible or probable AD, NINCDS-ADRDA, and DSM-IV)	Not clearly stated, changes in MMSE and ADAS-Cog	3 years	Lower level of education	High
Miranda et al. (2015)	129 AD and AD+CVD patients (97 completed the study)	Donepezil, rivastigmine, galantamine	Clinical (probable AD, NIAA, Ballinger et al., 2017; AD+CVD, NINDS-AIREN)	Improvement of 2+ points on MMSE	12 months	Lower education, lower income	Moderate

A registry analysis using artificial intelligence by Gallucci showed that “white collars,” including teachers, office workers, and professionals, were more likely to show a cognitive response on AChEI compared to blue collars (which included farmers, artisans, workmen, and tradesmen, with an expected lower education). The same study identified “being married” as another predictor of cognitive response, which would be related to the affective component of the cognitive reserve (Gallucci et al., 2016).

Conversely, two studies by Wattmo found that lower education was a predictor of cognitive response at 3 years in Swedish AD patients (Wattmo et al., 2011; Wattmo and Wallin, 2017), and a study by Miranda confirmed this result in Brazilian AD and mixed dementia patients at 1 year (adding lower income as a predictor, which might have been associated with lower education) (Miranda et al., 2015). This finding may not be in contrast with the previous ones if the timing of response assessment is considered, since studies evaluating “short-term” response (i.e., over months) would probably find patients who are still compensating for a more advanced AD pathology with high cognitive resources, while studies evaluating “long-term” response (i.e., over years) could show the typical effect of faster deterioration due to the more severe underlying pathology revealed by the failure of compensating factors. Indeed, basal forebrain metabolism seems to be enhanced in people with higher education and greater cognitive reserve, which could represent a compensatory effect that may be lost in later stages (Giacobini et al., 2022).

#### Demographic factors: Age, gender, race

Age has been extensively studied as a possible predictor. However, a premise should be made. Almost all studies included in the current review base the diagnosis of AD on clinical criteria, without the use of biomarkers. In 2019 Nelson described the previously under-recognized Limbic predominant Age-related TDP-43 Encephalopathy (LATE), a neuropathological entity with the same clinical features of AD, consisting of a predominantly amnestic dementia. However, “pure” LATE is more typical of the “oldest old,” and may exhibit a more gradual decline compared to AD (Nelson et al., 2019). Without biomarkers of AD pathology, some of the subjects enrolled in these studies may have been indeed LATE patients, which would invalidate the conclusions on the role of age as a predictor of response to AChEI. That said, a retrospective study by Boccardi found a differential time effect of age: younger age was a predictor of short-term cognitive response, while older age subjects had a better long-term response. This could be explained by the premise above, since younger subjects (with “true” AD pathology) would respond to AChEI in the typical period of months, while older subjects (possibly including LATE patients) would show a slower decline over years, when the effect of AChEI is expected to be clinically negligible (Boccardi et al., 2017). Similar considerations may be made for primary age-related tauopathy (PART), introduced by Crary et al. (2014).

A study by Wattmo found that older AD patients had a better cognitive response at 3 years compared to younger patients. This differential effect of age was present only in the subgroup of late-onset AD subjects, which may be explained by the previously introduced framework (older clinically-diagnosed late-onset AD patients could probably be LATE or PART patients) (Wattmo and Wallin, 2017). Another study by Wattmo on AD patients from the same cohort found older age to be predictive of cognitive response at 6 months across the whole sample, but this was limited to patients with baseline MMSE <22 (although the same association was noticed across all levels of ADAS-Cog at baseline) (Wattmo et al., 2011). A study by Cheng found younger age to be predictive of more rapid cognitive decline in Taiwanese AD patients on AChEI (Cheng et al., 2015). A *post-hoc* analysis of a RCT by Farlow found increased age to be a predictor of response to rivastigmine at 6 months; moreover, older patients experienced less adverse events (Farlow et al., 2015).

Conversely, Gallucci found that patients younger than 75 years showed a better response to AChEI (Gallucci et al., 2016). A study by Wattmo found younger age to be related to functional response at 6 months and 3 years, which would be expected based on the fact that cognition is progressively less relevant to functional status with increasing age-related comorbidities (Wattmo et al., 2012). Similarly, a study by Ho found that younger age was a predictor of global, but not cognitive, response to rivastigmine at 1 year (Ho et al., 2016).

Two studies failed to find an influence of age on cognitive response at 6 months in AD patients (Chen et al., 2017; Miranda et al., 2017), and so did a retrospective study by Perera at 3 years (Perera et al., 2014). Another study by Raschetti did not find an influence of age on cognitive response at 9 months, but older age (above 79 years old) was associated with more adverse reactions (Raschetti et al., 2005).

Although a sex dimorphism in the expression of cholinergic systems is reported (Weng et al., 2013), with greater tau-immunoreactivity in the NB of women and a hypothesized influence of estrogen (Scacchi et al., 2014; Giacobini et al., 2022), the cumulative evidence does not convincingly link gender and response to AChEI based on the cholinergic deficit framework. Different studies with rather large samples support each of the possible options, namely that either male or female genders are predictors of response, or that gender is not associated with response. However, the studies reporting this last conclusion involve the highest number of subjects (more than 6,000) (Raschetti et al., 2005; Patterson et al., 2011; Perera et al., 2014; Braga et al., 2015; Miranda et al., 2017), while the ones claiming that gender is indeed a predictor cumulatively involve at most 3,000 patients. The heterogeneity of the methodological aspects of these studies does not allow for more detailed comparisons. The studies finding gender differences in response are discussed here below.

The study by Gallucci found that male gender was predictive of cognitive response to AChEI (Gallucci et al., 2016). The *post-hoc* analysis of the OPTIMA RCT by Molinuevo found male gender to be a predictor of cognitive response to rivastigmine at 12 months (Molinuevo et al., 2015). Two studies by Wattmo identified male gender, in particular in late-onset AD, to be a predictor of cognitive response to AChEI at 6 months and 3 years (Wattmo et al., 2011; Wattmo and Wallin, 2017). A study by Clarelli found that male gender predicted cognitive response at 1 year only in mild AD cases (Clarelli et al., 2016).

Another study by Wattmo found that Swedish AD males living with family members had less risk of institutionalization compared to females, even though this difference might be at least partially explained by outdated cultural factors related to a higher involvement of women in caregiver duties, which may be expected to change with time (Wattmo et al., 2018). Another study by the same group found that living with a family member, irrespective of gender, was associated with functional response at 3 years (Wattmo et al., 2012). Conversely, a study by Scacchi found female gender to be associated with cognitive response to rivastigmine and donepezil at 15 months (Scacchi et al., 2014), and so did Modrego in terms of cognitive response to galantamine at 6 months (Modrego et al., 2009).

A retrospective study by Perera found that non-white groups of patients responded better to AChEI therapy at 3 years (Perera et al., 2014).

The studies on the demographic factors included in this section are listed in Table 6.

**Table 6:** Demographic factors.

**Reference**	**Population**	**Treatment**	**Diagnosis**	**Response criteria**	**Follow-up**	**Predictor**	**Risk of bias**
Wattmo et al. (2012)	784 AD patients	Donepezil, galantamine, rivastigmine	Clinical (possible or probable AD, NINCDS-ADRDA, and DSM-IV)	No deterioration in IADL or PSMS at 6 months	3 years	Younger age	High
Cheng et al. (2015)	353 AD patients	Donepezil, galantamine, rivastigmine	Clinical (probable AD, NINCDS-ADRDA 1984)	Longer TMSE decline-free survival (Survival analysis, using TMSE decline of 3+ points as endpoint)	Survival analysis, median 46.6 months	Lower total MTA score and older age	Low
Farlow et al. (2015)	716 severe AD patients (628 included)	Rivastigmine 13.3 or 4.6 mg	Clinical (probable AD, NINCDS-ADRDA)	Improvement/improvement or no change on ADCS-CGIC	24 weeks	Treatment with 13.3 mg/24 h rivastigmine patch and increased age	Some concerns—Low
Touchon et al. (2006)	994 AD patients (moderately-severe), 578 completed the study	Donepezil, rivastigmine	DSM-IV criteria and NINCDS-ADRDA	Severe impairment battery, no clear definition	2 years	Age younger than 75 and BuChE wt (for treatment with rivastigmine)	Some concerns—Moderate
Gallucci et al. (2016)	84 AD patients + 6 AD+CVD patients	Donepezil, galantamine, rivastigmine	Clinical (NINCDS-ADRDA and NINDS-AIREN, not stated if possible included)	Less than 2 points deterioration of MMSE per year	Up to 4 years	Male gender, <75 y.o.	High
Wattmo and Wallin (2017)	1,017 AD patients	Donepezil, galantamine, rivastigmine	Clinical (possible or probable AD, NINCDS-ADRDA, and DSM-IV)	No deterioration in MMSE	3 years	Male gender, older age; early onset and late onset AD are in general not a predictor	High
Ho et al. (2016)	87 AD patients	Rivastigmine	Not clearly stated (presumably probable AD, NINCDS-ADRDA)	No deterioration on MMSE or CDR-SB	1 year	Younger age	Moderate
Miranda et al. (2015)	129 AD and AD+CVD patients (97 completed the study)	Donepezil, galantamine, rivastigmine	Clinical (probable AD, NIAA, Ballinger et al., 2017; AD+CVD, NINDS-AIREN)	Improvement of 2+ points on MMSE	12 months	Age is not a predictor	Moderate
Chen et al. (2017)	63 AD patients	Rivastigmine	Clinical (NINCDS-ADRDA, not stated if probable or possible)	Improvement in either MMSE or CDR	6 months	Age is not a predictor	Moderate
Perera et al. (2014)	2,460 patients	Donepezil, galantamine, rivastigmine	Not stated	Change in MMSE	4 years (1 year prior to AChEI initiation to 3 years after)	Non-white groups; age and gender are not predictors	High
Raschetti et al. (2005)	5,642 AD patients (2,853 completed the study)	Donepezil, galantamine, rivastigmine	Clinical (probable AD, NINCDS-ADRDA)	2+ points improvement on MMSE	9 months	Age is not a predictor, but older age is associated with more adverse reactions; gender is not a predictor	Moderate
Molinuevo et al. (2015)	536 AD patients (ITT analysis)	Rivastigmine	Clinical (probable AD, NINCDS-ADRDA)	Improvement of 4+ points on ADAS-Cog, with no decline on ADCS-IADL	48 weeks	R 13.3 mg/24 h patch and male gender	Some concerns—Low
Wattmo et al. (2011)	843 AD patients	Donepezil, galantamine, rivastigmine	Clinical (possible or probable AD, NINCDS-ADRDA, and DSM-IV)	Not clearly stated, changes in MMSE and ADAS-Cog	3 years	Male gender, older age	High
Clarelli et al. (2016)	169 AD patients (a subset with MMSE >19 was selected to be consistent with Braga et al., 2015)	Donepezil, galantamine, rivastigmine	Clinical (probable AD, NINCDS-ADRDA) (Fritz et al., 2018)	Non-worsening of MMSE (as in Braga et al., 2015) and improvement of 2+ points in MMSE (as in Weng et al., 2013)	1 year	Male gender (only in mild cases)	Low
Braga et al. (2015)	177 AD patients (at 6 months), 147 AD patients (at 2 years)	Donepezil, galantamine, rivastigmine	Clinical (probable AD, NINCDS-ADRDA)	MMSE improvement or no deterioration	2 years	Gender is not a predictor	Low
Patterson et al. (2011)	165 AD patients (81 completed the whole study)	Donepezil, galantamine, rivastigmine	Clinical (probable AD, NINCDS-ADRDA, and DSM-IV)	Improvement in MMSE at 3–9 months (early cognitive response); no decline in MMSE at 15–24 months (late cognitive response)	Up to 24 months	Gender is not a predictor	Moderate
Wattmo et al. (2018)	881 AD patients	Donepezil, galantamine, rivastigmine	Clinical (possible or probable AD, NINCDS-ADRDA, and DSM-IV)	Institutionalization: permanent entry to a licensed skilled-nursing facility with 24-h care (not clearly defined as a response); improvement in MMSE, CIBIC (1–3), IADL or PSMS at 6 months (but used as predictors)	3 years	Being a male living with a family member is a predictor of delayed institutionalization	High
Scacchi et al. (2014)	184 AD patients	Donepezil, rivastigmine	Clinical (probable AD, NINCDS-ADRDA, and DSM-IV)	Change in MMSE compared to untreated patients	15 months	Female gender, and carriers of P and X allele on ESR1 rs2234693 and rs9340799 SNPs (only for donepezil)	Low
Modrego et al. (2009)	54 AD patients (50 included, 43 stayed on G)	Galantamine (but switching to donepezil allowed)	Clinical (probable AD, NINCDS-ADRDA)	Changes in ADAS-Cog, NPI and DAD, stroke, death	6 months	Women, lower IMT (especially in men)	Low

#### Rate of progression

A study by Wallin found faster pre-treatment progression to be a predictor of response to AChEI at 6 months in AD patients (Wallin et al., 2009). The same finding was confirmed in an observational study by Sobow, which showed that fast progressors (subjects who had more than 3 points/year loss on MMSE before treatment) were more likely to exhibit a cognitive response to rivastigmine at 6 months (Sobow et al., 2007). The relevant studies are summarized in Supplementary Table S3.

#### Short-term response

Several studies evaluated whether short-term response (usually at 3 months) could be a predictor of subsequent long-term response. A retrospective study by Boccardi found that short-term cognitive responders did not progress differently compared to short-term non-responders (Boccardi et al., 2017).

A study by Mossello found that cognitive response at 3 months predicted better outcomes on MMSE and ADL at 9 months (Mossello et al., 2004), and so did a study by Raschetti (with a rather compelling OR of 20.6) (Raschetti et al., 2005). A study by Miranda found that being a very good responder (improvement of 3 or more points on MMSE) at 3 and 6 months predicted cognitive response at 1 year (Miranda et al., 2015). A retrospective study by Horikoshi (which however did not provide a separate analysis for patients on memantine) concluded that cognitive response at 6 months, defined as a change in MMSE lower than the lower extreme of the confidence interval of the “natural” change in MMSE in untreated AD patients, was a predictor of subsequent cognitive response at 12 and 24 months (Horikoshi et al., 2020).

An interesting study by Wattmo found that improvement or no deterioration in a combination of MMSE, CIBIC, IADL, and Physical Self-Maintenance Scale (PSMS) at 6 months was a predictor of delayed institutionalization in Swedish AD patients, with a positive relationship between the subject's number of scales with response to AChEI and time to nursing home placement (Wattmo et al., 2018). A study by the same group found functional response on ADL at 6 months to be predictive of subsequent functional response at 3 years (Wattmo et al., 2012).

Another *post-hoc* analysis of a RCT by Ohnishi found that improvement on the word recognition subtest of ADAS-Cog by at least 1.5 points at 4 weeks of treatment with galantamine was predictive of cognitive response at 4 weeks, with a sensitivity of 75% and a specificity of 64%. However, improvement on ideational, orientation and word recognition subtests altogether had a higher specificity of 75% (Ohnishi et al., 2014).

A study by Palmqvist found that a 16 s or more improvement in the color form of A Quick Test (AQT) at 8 weeks was a predictor of subsequent response to AChEI at 6 months (defined with the same criteria) in Swedish AD patients (Palmqvist et al., 2010).

Taken together, these papers provide quite consistent evidence of a correlation between short and long-term response. These studies are listed in Table 7.

**Table 7:** Short-term response.

**Reference**	**Population**	**Treatment**	**Diagnosis**	**Response criteria**	**Follow-up**	**Predictor**	**Risk of bias**
Boccardi et al. (2017)	628 AD patients	Donepezil, galantamine, rivastigmine	Not stated	MMSE stabilization or improvement at 3 months	3 years	Short-term response is not a predictor	Moderate
Droogsma et al. (2015)	335 AD patients	Donepezil, galantamine, rivastigmine (memantine)	Clinical (NINCS-ADRDA, not stated if probable or possible)	Change in MMSE (no deterioration in MMSe as initial response)	Up to 10 years	Initial response after 6 months is not a predictor of subsequent long-term response	High
Mossello et al. (2004)	212 AD patients	Donepezil, galantamine, rivastigmine	Clinical (probable AD, NINCDS-ADRDA)	No deterioration in MMSE at 3 months; not clearly stated, but change in MMSE, ADL, and IADL at 9 months	9 months	No deterioration on MMSE after 3 months of therapy	High
Raschetti et al. (2005)	5642 AD patients (2,853 completed the study)	Donepezil, galantamine, rivastigmine	Clinical (probable AD, NINCDS-ADRDA)	2+ points improvement on MMSE	9 months	Absence of concomitant diseases and response at 3 months	Moderate
Miranda et al. (2015)	129 AD and AD+CVD patients (97 completed the study)	Donepezil, galantamine, rivastigmine	Clinical (probable AD, NIAA, Ballinger et al., 2017; AD+CVD, NINDS-AIREN)	Improvement of 2+ points on MMSE	12 months	Being a very good responder at 3 and 6 months	Moderate
Calabria et al. (2009)	427 AD patients (226 patients completed the study)	Donepezil, rivastigmine	Clinical (probable AD, NINCDS-ADRDA)	Based on the distribution of MMSE change between baseline and follow-up: above 75th percentile very good responders, 50–70th good responders, 25–50th bad responders, under 25th very bad responders	21 months	Greater improvement at 3 months	High
Rota et al. (2007)	203 AD patients	Donepezil, galantamine, rivastigmine	Clinical (probable AD, NINCDS-ADRDA)	Change in MMSE and BADL-IADL	30 months	Higher MMSE (only for MMSE >18), BADL and IADL improvement at 3 months, all for response at 9 months	Moderate
Horikoshi et al. (2020)	110 AD patients	Donepezil, galantamine, rivastigmine, memantine	Clinical (ICD-10)	Change in MMSE the same or lower of lower confidence interval of mean change in MMSE (Rey et al., 2018) without treatment at 6 months; mean change in MMSE from baseline to 12 and 24 months	24 months	Same or lower change in MMSE than lower confidence interval of mean change in MMSE without treatment at 6 months	Moderate
Wattmo et al. (2018)	881 AD patients	Donepezil, galantamine, rivastigmine	Clinical (possible or probable AD, NINCDS-ADRDA, and DSM-IV)	Institutionalization: permanent entry to a licensed skilled-nursing facility with 24-h care (not clearly defined as a response); improvement in MMSE, CIBIC (1–3), IADL, or PSMS at 6 months (but used as predictors)	3 years	Improvement or no deterioration in a combination of MMSE, CIBIC, IADL and PSMS at 6 months are a predictor of delayed institutionalization	High
Farlow et al. (2015)	716 severe AD patients (628 included)	Rivastigmine 13.3 or 4.6 mg	Clinical (probable AD, NINCDS-ADRDA)	Improvement/improvement or no change on ADCS-CGIC	24 weeks	Minimal worsening, no change or improvement on ADCS-CGIC at week 8 and 16	Some concerns—Low
Ohnishi et al. (2014)	303 AD patients	Galantamine	Clinical (probable AD, NINCDS-ADRDA)	4+ points improvement on ADAS-Cog Japanese version	24 weeks	Improvement on word recognition by at least −1.5 points on ADAS-Cog after 4 weeks of treatment	Some concerns—Low
Palmqvist et al. (2010)	75 AD patients	Donepezil, galantamine, rivastigmine	Clinical (probable or possible AD, NINCDS-ADRDA, see Borroni and Barrantes, 2021)	16 s impovement on A Quick Test—color form at 8 weeks or 3+ points improvement on MMSE at 8 weeks; change in AQT at 6 months	32 ± 19 months	16 s + improvement on AQT (A Quick Test—color form) after 8 weeks	Moderate

#### Measures of cognitive or functional impairment at baseline

There are controversial results regarding cognitive and functional impairment at baseline, which may be interlaced with the concept of cognitive reserve and with pre-treatment progression. On one hand, better preserved cognitive and functional status at baseline may be found in patients with slow progression due to a variety of factors, which could be maintained during treatment (or even not be influenced by AChEI at all, see Age section). On the other hand, the same profile may be found in individuals with higher cognitive reserve, which would typically show a faster progression at a certain subsequent point due to a more severe underlying pathology “masked” by the resources of the patients. These two possibilities may explain the contrasting findings. The studies analyzed in this section are summarized in Table 8.

**Table 8:** Measures of cognitive or functional impairment at baseline.

**Reference**	**Population**	**Treatment**	**Diagnosis**	**Response criteria**	**Follow-up**	**Predictor**	**Risk of bias**
Boccardi et al. (2017)	628 AD patients	Donepezil, galantamine, rivastigmine	Not stated	MMSE stabilization or improvement at 3 months	3 years	Lower MMSE, higher ADL (for response at 3 months)	Moderate
Mossello et al. (2004)	212 AD patients	Donepezil, galantamine, rivastigmine	Clinical (probable AD, NINCDS-ADRDA)	No deterioration in MMSE at 3 months; not clearly stated, but change in MMSE, ADL, and IADL at 9 months	9 months	Lower MMSE at baseline	High
Pakrasi et al. (2003)	150 demented patients (137 for cognitive response analysis)	Donepezil, galantamine, rivastigmine	Clinical (dementia, ICD-10; then AD, NINCDS-ADRDA—doesn' state if possible included; DLB, McKeith)	Clinical response: improvement in global clinical condition. Cognitive response: 2+ points improvement on MMSE	3–4 months	Lower MMSE	High
Horikoshi et al. (2020)	110 AD patients	Donepezil, galantamine, rivastigmine, memantine	Clinical (ICD-10)	Change in MMSE the same or lower of lower confidence interval of mean change in MMSE (Rey et al., 2018) without treatment at 6 months; mean change in MMSE from baseline to 12 and 24 months	24 months	Lower MMSE	Moderate
Wallin et al. (2009)	191 AD patients (161 with CSF biomarkers)	Donepezil, galantamine, rivastigmine	Clinical (probable or possible AD, NINCDS-ADRDA, and DSM-IV)	2+ points improvement in MMSE (other models: 2+ points improvement in MMSE and CIBIC 1–3); 2+ points improvement in ADAS-Cog; 4+ points improvement in ADAS-Cog	6 months	Lower MMSE	Moderate
Weng et al. (2013)	204 AD patients	Donepezil, galantamine, rivastigmine	Clinical (probable AD, NINCDS-ADRDA)	2+ points improvement in MMSE	6 months	Lower MMSE	Low
Clarelli et al. (2016)	169 AD patients (a subset with MMSE >19 was selected to be consistent with Braga et al., 2015)	Donepezil, galantamine, rivastigmine	Clinical (probable AD, NINCDS-ADRDA) (Fritz et al., 2018)	Non-worsening of MMSE (as in Braga et al., 2015) and improvement of 2+ points in MMSE (as in Weng et al., 2013)	1 year	Lower MMSE	Low
Calabria et al. (2009)	427 AD patients (226 patients completed the study)	Donepezil, rivastigmine	Clinical (probable AD, NINCDS-ADRDA)	Based on the distribution of MMSE change between baseline and follow-up: above 75th percentile very good responders, 50–70th good responders, 25–50th bad responders, under 25th very bad responders	21 months	Lower MMSE	High
Frankfort et al. (2007)	179 AD patients	Rivastigmine	Clinical (probable or possible AD, NINCDS-ADRDA)	Change in MMSE and CAMCOG	6 months	MMSE <20	Low
Chen et al. (2017)	63 AD patients	Rivastigmine	Clinical (NINCDS-ADRDA, not stated if probable or possible)	Improvement in either MMSE or CDR	6 months	Lower baseline CDR, lower baseline MMSE	Moderate
Perera et al. (2014)	2,460 patients	Donepezil, galantamine, rivastigmine	Not stated	Change in MMSE	4 years (1 year prior to AChEI initiation to 3 years after)	Lower MMSE	High
Yamagata et al. (2010)	23 AD patients (non-responders to D 5 after approximately 15 months of therapy)	Donepezil	Clinical (probable AD, NINCDS-ADRDA)	No deterioration in MMSE	12 weeks	IGF-I ≤ 99 ng/ml and MMSE ≤ 18 in patients non-responders to donepezil 5 mg (and increased to 10 mg)	Moderate
Lu et al. (2016)	85 AD patients	Donepezil	Not stated	No deterioration in MMSE	At least 3 months (but it's not clear when response is assessed)	Lower MMSE is a predictor of poor response	High
Droogsma et al. (2015)	335 AD patients	Donepezil, galantamine, rivastigmine, memantine	Clinical (NINCS-ADRDA, not stated if probable or possible)	Change in MMSE (no deterioration in MMSe as initial response)	Up to 10 years	Lower MMSE is a predictor of poor response	High
Tei et al. (2008)	50 AD patients and 56 HC	Donepezil 5 mg	Clinical (probable AD, NINCDS-ADRDA)	4+ points improvement on MMSE	16 weeks	IGF-I ≥110 ng/ml and MMSE ≥15	Moderate
Rota et al. (2007)	203 AD patients	Donepezil, galantamine, rivastigmine	Clinical (probable AD, NINCDS-ADRDA)	Change in MMSE and BADL-IADL	30 months	MMSE at baseline and 3 months (only in MMSE >18) for response at 9 months	Moderate
Sobow et al. (2007)	54 AD patients	Rivastigmine	Clinical (AD, NINCDS-ADRDA, not stated if possible included; exclusion of patients who also fulfilled criteria for other dementia syndromes)	3+ points improvement on ADAS-Cog (stability if <2 points variation)	6 months	Higher initial ADAS-Cog	Moderate
Gallucci et al. (2016)	84 AD patients + 6 AD+CVD patients	Donepezil, galantamine, rivastigmine	Clinical (NINCDS-ADRDA and NINDS-AIREN, not stated if possible included)	Less than 2 points deterioration of MMSE per year	Up to 4 years	MMSE > 20, CDR 0.5–1, higher ADL	High
Modrego et al. (2009)	54 AD patients (50 included, 43 stayed on G)	Galantamine (but switching to donepezil allowed)	Clinical (probable AD, NINCDS-ADRDA)	Changes in ADAS-Cog, NPI and DAD, stroke, death	6 months	MMSE, Blessed Dementia Rating scale, and ADAS-Cog are not predictors	Low
Wattmo et al. (2018)	881 AD patients	Donepezil, galantamine, rivastigmine	Clinical (possible or probable AD, NINCDS-ADRDA, and DSM-IV)	Institutionalization: permanent entry to a licensed skilled-nursing facility with 24-h care (not clearly defined as a response); improvement in MMSE, CIBIC (1–3), IADL or PSMS at 6 months (but used as predictors)	3 years	Better MMSE, CIBIC, IADL, and PSMS are a predictor of delayed institutionalization	High
Wattmo and Wallin (2017)	1,017 AD patients	Donepezil, galantamine, rivastigmine	Clinical (possible or probable AD, NINCDS-ADRDA, and DSM-IV)	No deterioration in MMSE	3 years	Less impaired IADL (only in late-onset AD patients); better cognitive status	High
Wattmo et al. (2011)	843 AD patients	Donepezil, galantamine, rivastigmine	Clinical (possible or probable AD, NINCDS-ADRDA, and DSM-IV)	Not clearly stated, changes in MMSE and ADAS-Cog	3 years	More severe impairment (initial better response, but faster decline subsequently)	High
Wattmo et al. (2012)	784 AD patients	Donepezil, galantamine, rivastigmine	Clinical (possible or probable AD, NINCDS-ADRDA, and DSM-IV)	No deterioration in IADL or PSMS at 6 months	3 years	Better preserved cognition (MMSE and ADAS-Cog) at baseline and more impaired ADL	High
Fukui and Taguchi (2005)	55 AD patients (50 completed the studies)	Donepezil 5 mg	Clinical (probable AD, ICD-10 and NINCDS-ADRDA)	Improvement of 2+ points on the Clock-drawing test (Marucci et al., 2020 for scoring) for true responders, ±2 points on the CDT for unchanged	12 months	High Revised Hasegawa Dementia scale at baseline and high blood pressure	Moderate
Graff-Radford et al. (2012)	54 DLB patients	Donepezil, galantamine, rivastigmine	Clinical (probable DLB, McKeith et al., 2004)	Reliable change in Dementia Rating Scale: reliable improvement with 9+ points increase in <15 months and 10+ >15 months, reliable decline 6+ decrease in <15 months and 7+ decrease >15 months, stability in between	1 year	Worse cognitive performance at baseline on the Dementia Rating Scale (especially on attention and conceptualization subscores)	Low
Miranda et al. (2015)	129 AD and AD+CVD patients (97 completed the study)	Donepezil, galantamine, rivastigmine	Clinical (probable AD, NIAA, Ballinger et al., 2017; AD+CVD, NINDS-AIREN)	Improvement of 2+ points on MMSE	12 months	Lower CDR	Moderate

As for MMSE at baseline, a retrospective study by Boccardi found that lower MMSE was a predictor of cognitive response at 3 months (Boccardi et al., 2017), and so did the studies by Mossello (Mossello et al., 2004) and Pakrasi (Pakrasi et al., 2003). The studies by Horikoshi, Wallin and Weng found lower MMSE in responders at 6 months (Wallin et al., 2009; Weng et al., 2013; Horikoshi et al., 2020), while the study by Clarelli found lower MMSE in responders at 1 year (Clarelli et al., 2016), and a study by Calabria confirmed that finding at 21 months (Calabria et al., 2009). Another study by Frankfort found that MMSE lower than 20 was a predictor of 6-month cognitive response to rivastigmine on the Cambridge Cognitive Examination compared to historical controls. The response was also evident on the memory and attention/calculation domains of the same scale (Frankfort et al., 2007). A study by Chen found that lower MMSE was a predictor of cognitive response to rivastigmine at 6 months (Chen et al., 2017). A retrospective study by Perera on a large set of patients found that lower MMSE at baseline was associated with subsequent MMSE improvement at 6 months and even at 3 years (Perera et al., 2014). A study by Yamagata found that among non-responders to donepezil 5 mg, patients with MMSE under 19 (and serum IGF-1 below 99) were more likely to respond to an increase of donepezil to 10 mg for 3 months (Yamagata et al., 2010).

Conversely, a study by Lu found that cognitive non-responders exhibited a lower MMSE at baseline (Lu et al., 2016), and the same did Droogsma (Droogsma et al., 2015), and Tei (Tei et al., 2008). A study by Rota found that patients with higher MMSE at baseline (MMSE > 18) had a better cognitive response at 21 months, although the study had a significant drop-out rate which could have influenced the results (Rota et al., 2007). A study by Sobow found that cognitive responders at 6 months had higher ADAS-Cog at baseline (Sobow et al., 2007).

A study by Modrego found no influence of baseline MMSE, Blessed Dementia Rating Scale, or ADAS-Cog on response to galantamine (Modrego et al., 2009).

A study by Wattmo found that a better cognitive and functional status at baseline was a predictor of delayed institutionalization (Wattmo et al., 2018), and another study by the same group found better cognitive status at baseline to be a predictor of cognitive response to AChEI at 3 years, whereas higher IADL scores were predictors of cognitive response only in late-onset AD patients (Wattmo and Wallin, 2017). Another study by Wattmo found that more severely impaired patients had a better response initially, followed by a faster decline over the course of 3 years (Wattmo et al., 2011). Better preserved cognition, together with more impaired ADL (for which an explanation was not provided), was found to be predictive of functional response at 6 months in another study on the same cohort (Wattmo et al., 2012).

A study by Fukui found that a higher score on the Hasegawa Dementia scale (which implies better cognition) at baseline was a predictor of response at 3 months, whereas a lower baseline performance on the Clock Drawing test, rated with a 15-point system, was found to be a predictor of response to donepezil at 3 months (measured with the same test). A higher Hasegawa Dementia scale to Clock Drawing test ratio, i.e., preserved verbal cognition with more selective executive, visuo-spatial, and constructional impairment, was found to be a predictor of response, even though these domains do not seem to be necessarily related to cholinergic impairment (Fukui and Taguchi, 2005).

A retrospective study by Graff-Radford found that worse performance on the Mattis Dementia Rating Scale was predictive of cognitive response to AChEI at 1 year in patients with DLB, especially on attention and conceptualization subscores. This would reflect a more pronounced cholinergic impairment (Graff-Radford et al., 2012).

A study by Miranda found that lower CDR at baseline was predictive of cognitive response to AChEI at 1 year (Miranda et al., 2015).

#### Cardiovascular risk factors

A study by Connelly investigated the role of current smoking, which could be potentially related to AChEI therapy through the action on nicotinic receptors. However, the authors did not find any statistically significant difference in response to AChEI according to smoking status. Surprisingly, smokers showed a significant improvement in the Digit Symbol Subtraction Test, compared to non-smokers, even after adjusting for age. The improvement in this test, which would reflect cholinergic-dependent processing speed, did not correlate with a good broader response in smokers (even though such correlation was found in non-smokers). However, the paper does not state which drugs have been used (Connelly and Prentice, 2005). A study by Gallucci, which used artificial intelligence to highlight possible associations between variables in a registry of AD patients, suggested that non-smoking was a predictor of cognitive response (Gallucci et al., 2016). Given the inconclusive results addressing the matter, and the advisable worldwide cessation of smoke, it is likely that new evidence on the topic will not be available. The same paper by Gallucci found that not drinking alcohol was associated with cognitive response, a finding that is quite interesting considering that the Italian region in which the study was conducted is famous for its tradition of alcohol consumption (Gallucci et al., 2016).

Although it was not the primary analysis of the study, a work by Connelly showed that BMI was greater in non-responders to AChEI at 6 months (Connelly et al., 2019).

A paper by Fukui found higher blood pressure to be a predictor of response to donepezil at 3 months (measured with the Clock Drawing test) (Fukui and Taguchi, 2005). A study by Ho found that hypertension was associated with cognitive (but not global) response to rivastigmine at 1 year (Ho et al., 2016). However, it is hard to find an explanation for this association. Conversely, a study by Connelly found that hypertension was a predictor of poor response at 6 months, but only in combination with white matter lesions (Connelly et al., 2005a). A study by Modrego found that the presence of hypertension did not influence clinical response to galantamine (Modrego et al., 2009).

The same study by Modrego found that a higher intima-media carotid thickness was predictive of poor cognitive and neuropsychiatric response to galantamine at 6 months. According to the authors, this is related to increased stiffness of the arterial wall, which might impair perfusion and cerebrovascular autoregulation, leading to reduced drug penetration in the brain. However, the association between intimate-media thickness and response was rather weak (but higher in males) (Modrego et al., 2009).

The study by Fukui found that hypercholesterolemia was a predictor of poor response (Fukui and Taguchi, 2005), and a study by Borroni found that lower serum cholesterol (under 220 mg/dl) was associated with better cognitive performances at 1 year (Borroni et al., 2003). At least one study provided evidence that hypercholesterolemia is associated with decreased cortical ACh, reduction of basal forebrain cholinergic neurons, neuroinflammation, and higher levels of cortical amyloid-beta, tau, and phospho-tau in adult rats (Ullrich et al., 2010). If confirmed in humans, the response to AChEI observed in patients with low cholesterol levels might therefore be found in a more preserved cholinergic system with less advanced AD pathology.

Taken together, these findings suggest that cardiovascular risk factors are associated with poor response, which we hypothesize might be partially explained by an increased vascular cognitive impairment. The studies mentioned in this section are summarized in Supplementary Table S4.

#### Diagnosis

The retrospective study by Perera found VaD to be a predictor of poor response at 3 years, while the authors did not find any effect of a diagnosis of DLB on response (Perera et al., 2014). A study by Wattmo did not find any differences in early-onset AD vs. late-onset AD in terms of cognitive response at 3 years (Wattmo and Wallin, 2017).

#### Other drugs and comorbidities

A retrospective study by Perera found that the use of antipsychotics, gastrointestinal drugs, and anti-platelet and anticoagulants were predictors of poor cognitive response to AChEI at 3 years (Perera et al., 2014). A study by Wattmo confirmed that no antipsychotic use was associated with cognitive response to AChEI at 3 years in late-onset AD patients. This finding could be possibly due to the fact that antipsychotic use may be warranted by the presence of BPSD, which are expected to be present later in the natural history of the disease, when AChEI are less likely to be effective (Wattmo and Wallin, 2017). Moreover, the fact that many antipsychotics have known anticholinergic effects may also partially explain the findings. Unfortunately, the anticholinergic burden of possible co-treatments is often not examined in most of the available literature on AD.

Two other studies by Wattmo found NSAIDs (and anti-platelet) use to be a predictor of cognitive and functional response to AChEI at 6 months, respectively, supposedly due to their neuroprotective effects (Wattmo et al., 2011, 2012). One of these studies found that a lower number of medications was predictive of functional response at 6 months, which might be related to the impact of more comorbidities on dependency in basic and instrumental activities of daily living (Wattmo et al., 2012).

A study by Raschetti found that the absence of concomitant diseases was associated with cognitive response to AChEI at 9 months, and with a lower probability of experiencing adverse drug reactions (together with the use of other CNS drugs), which might explain at least in part the former association (Raschetti et al., 2005).

It is not clear whether these findings would rather identify a subgroup of patients with a slower progression of cognitive and functional decline even independently of AChEI therapy.

#### Serum IGF-1 levels

A study by Tei found that IGF-1 levels over 110 ng/mL were predictive of cognitive response to donepezil 5 mg at 4 months. In addition, donepezil increased serum IGF-1 levels in responders, but not in non-responders (Tei et al., 2008). The authors report that IGF-1 seems to protect neurons (especially in the hippocampus) from cell death due to amyloid-beta and hypoxic or ischemic injury, and it also appears to regulate Aβ clearance from the brain (Tei et al., 2008). However, the same group performed another study including only non-responders from the study by Tei, which were subsequently treated with donepezil 10 mg. In this subpopulation, the authors found that patients with IGF-1 lower than 99 ng/ml and MMSE under 19 were more likely to respond after 3 months of donepezil 10 mg. Taken together, these findings mean that higher levels of IGF-1 are useful to determine which patients will respond to low dose donepezil, while in non-responders with low levels of IGF-1 a further clinical benefit could be achieved by increasing the dose, an effect which will probably not be evident in non-responders with higher levels of IGF-1. The authors speculate that in the latter subgroup IGF-1 receptors in the brain may be downregulated by long-term exposure to increased IGF-1, which may contribute to neurodegeneration (Yamagata et al., 2010).

#### Thyroid function

A study by Kapaki found that higher T4 and fT4 levels were predictive of cognitive response to donepezil at 4 months in AD patients (Kapaki et al., 2006). Chang et al. confirmed this finding at 6 months (Chang et al., 2018). Both studies showed that donepezil treatment reduced T4 levels. Moreover, in the study by Kapaki, AD patients had more abnormal anti-TPO levels (which were affected by gender, being higher in females, and which were reduced by treatment) compared to controls, while in the case-control study by Chang AD patients had lower levels of T3 and fT3 (which was however unaffected by donepezil) (Kapaki et al., 2006; Chang et al., 2018). No robust explanation for these findings exists, but the authors of these studies speculate that donepezil might act beyond AChE inhibition, partially correcting a relative “brain hypothyroidism” through an enhanced conversion of T4 into T3 within the brain. The increased peripheral T4 levels might indeed reflect lower T3 levels in the brain. Moreover, they report that thyroid hormones have been known to modulate ChAT activity, and thus higher T4 might identify a subgroup of patients with a more pronounced cholinergic deficit (Kapaki et al., 2006; Chang et al., 2018).

#### Vitamin B12

A retrospective study by Cho explored the association between vitamin B12 levels at baseline and cognitive response to rivastigmine and donepezil in a Taiwanese sample of AD patients, finding a negative correlation with cognitive decline, after adjustment for possible confounding factors (however, it was not clear whether the authors adjusted for sex) (Cho et al., 2018). Even though all patients were above the threshold for vitamin B12 because of inclusion criteria, significantly more women were in the optimal vitamin B12 group (above the median, 436 ng/L), which is in line with previous literature showing higher values of vitamin B12 in elderly and non-elderly women (Siek et al., 1990; Margalit et al., 2018). Anyway, the lack of a control group not treated with AChEI does not allow to evaluate whether suboptimal vitamin B12 levels are indeed a predictor of poor response to AChEI, or whether they had an independent role in cognitive decline.

#### Markers of amyloidopathy

A study by Modrego found that baseline lower levels of plasmatic Aβ-40 were correlated with a better response to galantamine at 6 months in both ADAS-Cog and NPI scales. Interestingly, while the treatment determined an increase of plasmatic Aβ-42 levels and a decrease of plasmatic Aβ-40 levels, an increase in such levels correlated with a higher improvement in ADAS-Cog. The authors hypothesized that this could be related either to a displacement of Aβ-40 from plasmatic proteins, or to an effect on APP processing through stimulation of muscarinic M1 receptors (which would result in a reduction in CSF Aβ-40 levels) (Modrego et al., 2008). A study by Sobow found that a more pronounced increase of plasmatic Aβ-42 levels after 1 month of treatment with rivastigmine was predictive of subsequent cognitive response at 6 months in AD patients (Sobow et al., 2007).

#### CSF biomarkers

A study by Wallin found that CSF levels of Aβ-42, total tau and phospho-tau were not predictive of cognitive response at 6 months in AD patients (Wallin et al., 2009).

All the studies on other clinical predictors (sections Diagnosis, Other drugs and comorbidities, Serum IGF-1 levels, Thyroid function, Vitamin B12, Markers of amyloidopathy, and CSF biomarkers) are summarized in Supplementary Table S5.

#### APOE

The role of APOE genotype, and especially of the ε4 allele, has been investigated by many authors, with contrasting results. Some authors hypothesized a relationship between APOE status and response to AChEI based on the neuropathological observation that AD subjects who were APOE-ε4 carriers exhibited less cholinergic markers in the temporal cortex and hippocampus (Rigaud et al., 2002). A few papers have tried to provide an analytical synthesis of the available evidence, which is beyond the scope of this review. A meta-analysis by Cheng (which included also tacrine and metrifonate) found that APOE-ε4 carrier status did not influence cognitive response to AChEI in AD patients (Cheng et al., 2018). However, another meta-analysis by Xiao concluded that APOE-ε4 may have a detrimental effect on response to donepezil through an interaction with CYP2D6 G-allele of the rs1080985 SNP (Xiao et al., 2016). The studies included in this section are listed in Supplementary Table S6.

That said, most studies did not find an effect of APOE status on cognitive response. The references of these negative studies can be found in the Supplementary material. Among the studies which identified APOE as a predictor, a small study by MacGowan on AD patients treated with galantamine showed that women who were APOE-ε4 non-carriers responded better to galantamine at 3 months compared to carriers (however, there were only three women in the former group), while in men it was the opposite. The difference between men and women reached statical significance in the APOE-ε4 carriers group, suggesting that men carriers may respond better to galantamine (MacGowan et al., 1998). A study by Wattmo found that APOE-ε4 non-carriers had a better cognitive response to AChEI at 3 years, as measured by ADAS-Cog (but not by MMSE, which is less sensitive to small changes) (Wattmo et al., 2011). A study by Csernansky found that APOE-ε4 allele number was positively correlated to change in CDR-SB at 2 years (i.e., the more alleles a subject carries, the worse its global status will be) (Csernansky et al., 2005). A retrospective study by Braga found that APOE-ε4 non-carriers were more likely to respond at 6 months, especially if their baseline MMSE was under 20, but the association was lost at 2 years (Braga et al., 2015).

Conversely, a study by Choi found that APOE-ε4 carriers showed significantly less worsening on ADAS-cog scores at 12 months than those in the APOE-ε4 non-carrier group (although the difference may be clinically irrelevant: 1.1-point increase vs. a 3.1-point increase). However, APOE-ε4 carriers had significantly higher levels of education, and no other significant difference was found in other cognitive or functional outcome measures (Choi et al., 2008). A study by Chen suggested a small positive effect of APOE-ε4 on cognitive response to rivastigmine at 6 months in Taiwanese AD patients (Chen et al., 2017), and so did a study by Devanand in MCI patients treated with donepezil for 1 year (Devanand et al., 2017). A study by Patterson found that APOE-ε4 was a predictor of cognitive response at 2 years only in patients with baseline MMSE above 21 (Patterson et al., 2011).

Finally, two studies by Lu found that APOE-ε3 non-carriers had a better cognitive response to donepezil than carriers (Lu et al., 2016, 2018).

#### Markers of reduced susceptibility of neurons to oxidative stress

A study by Paroni found rs7981045 GG genotype of FOXO1 to be predictive of poor response to AChEI in an Italian sample of AD patients. FOXO1 is implicated in the cellular response to oxidative stress, and it has been linked to AD through brain insulin resistance. It has been suggested that AChEI may protect cholinergic neurons by enhancing antioxidant activity, and it is possible that the GG genotype of FOXO1 directly contrasts this putative mechanism through increased production of reactive oxygen species (Paroni et al., 2014). However, further studies are needed to confirm this hypothesis.

#### ATP-binding cassette A and B (ABCA1 and ABCB1) polymorphisms

A study by Lu found that Han Chinese ABCA1 rs2230806 GG carriers had a better cognitive response compared to other rs2230806 genotypes, especially in APOE-ε3 non-carriers. A possible explanation for this finding would be the fact that donepezil increases the levels of soluble forms of APP, and ABCA1 furtherly neutralizes Aβ aggregation through APOE lipidation, by ABCA1-mediated cholesterol and phospholipid transfer, facilitating its elimination from the brain (Lu et al., 2018). The ABCA1 protein participates in this process by regulating APOE levels and function in the CNS (Jacobo-albavera et al., 2021). Thus, the yet to be confirmed effect of rs2230806 GG genotype might be an enhancement of ABCA1 activity resulting in a more efficient clearance of soluble Aβ from the brain. However, the role of rs2230806 is still not clear, with a meta-analysis reporting conflicting results in different populations (notably, it has been found to be protective against AD in Han Chinese) (Jacobo-albavera et al., 2021).

Moreover, a few SNPs of ABCB1, which encodes glycoprotein P, have been found to influence donepezil efflux from the brain to the blood, theoretically limiting the drug efficacy. However, a study by Magliulo failed to find significant associations between ABCB1 polymorphisms and either donepezil concentration/dose ratio or cognitive response in Italian AD patients, even though AD patients homozygous for the haplotype 1236T/2677T/3435T showed a tendency toward a better clinical response. The same patients exhibited a trend toward lower donepezil concentration/dose ratios compared to patients with other genotypes (Magliulo et al., 2011). An explanation of these findings could be that the T/T/T haplotype leads to reduced glycoprotein P activity, with higher donepezil levels in the brain due to reduced efflux, resulting in both lower blood concentrations and a better cognitive response.

#### Other genes

A genome-wide association study by Martinelli-Boneschi suggested that SNPs rs6720975 A allele could be predictive of cognitive response to AChEI at 18 months in Italian AD patients, while rs17798800 A allele could be a predictor of poor response. The former SNP maps in the intronic region of PRKCE, a protein-kinase C highly expressed in the hippocampus, with a putative role in the regulation of CHAT activity and Aβ production. The latter is an intergenic SNP associated with neurobeachin expression levels in peripheral bone-marrow cells (with a trend toward reduced expression of neurobeachin also in the brain of patients carrying rs17798800 A allele). Loss of neurobeachin has been shown to completely block synaptic transmission (Martinelli-Boneschi et al., 2013). Further studies are needed to confirm the findings of this study, and provide possible explanations for the association.

A study by Scacchi found carriers of both P and X alleles on ESR1 rs2234693 and rs9340799 SNPs to respond better than non-carriers to donepezil therapy at 15 months (notably, an inverse trend was observed for rivastigmine). The presence of the P allele of ESR1 seems to enhance the transcription of estrogen receptor α, which positively influences CHAT activity, resulting in higher levels of ACh (Scacchi et al., 2014).

The studies on FOX1, ABCA, and ABCB and other genes (sections Markers of reduced susceptibility of neurons to oxidative stress, ATP-Binding Cassette A and B (ABCA1 and ABCB1) polymorphisms, and Other genes) can be found in Supplementary Table S7.

#### Medial temporal lobe atrophy

Most studies found that medial temporal lobe preservation is associated with good cognitive response. While visual assessment (Visser et al., 2005; Cheng et al., 2015) and partially manual measurement (Connelly et al., 2005b; Csernansky et al., 2005) produced less sensitive and specific results, voxel-based methods, which might be less biased, gave more consistent results (Graff-Radford et al., 2012; Teipel et al., 2016; Um et al., 2017). The fact that different methods produce converging results strongly supports this association. Indeed, it has been showed that AD patients with hippocampal sparing have a more severe cholinergic deficit, which would explain why AChEI seem to work better in this population (Giacobini et al., 2022).

A study by Cheng found that higher medial temporal atrophy scores (visually assessed either on CT scans or MRI) were predictive of more rapid cognitive decline in Taiwanese AD patients on AChEI therapy, with a 2-year anticipation of a significant decrease in MMSE (3 or more points) (Cheng et al., 2015). A study by Visser found that medial temporal atrophy, scored with the same method, was not predictive of cognitive response to rivastigmine at 26 weeks, even though on a *post-hoc* analysis patients on low dose rivastigmine with medial temporal lobe atrophy had a significantly worse cognitive decline on both MMSE and ADAS-Cog (Visser et al., 2005).

A study by Connelly found that less medial temporal lobe atrophy at baseline, measured as the average of the distance between the medial wall of the lateral ventricle to the ambient cistern, then converted to a multiple of the median age-related value, was predictive of good response to AChEI. However, for a cut-off of 1 (i.e., less medial temporal lobe atrophy than the median age-related value), while the sensitivity was 89%, the specificity was quite low (only 30%), which means that temporal lobe atrophy might not be a good reason to exclude patients from therapy with AChEI (Connelly et al., 2005b).

A study by Csernansky found that smaller hippocampal volumes associated with deformation of the lateral and inferomedial surface (inward deformation of the hippocampal surface in proximity to the CA1 subfield and the subiculum) to be a negative predictor of response to donepezil at 2 years. Hippocampal volumes were measured by mapping a template MRI on target images and then calculating the volumes enclosed by transformed template hippocampal surfaces (Csernansky et al., 2005). The authors report that this pattern of atrophic changes in the hippocampus surface was found to predict the development of dementia in elderly subjects without dementia at the time of scanning, and to distinguish people with AD from people without dementia. Since the diagnosis of AD in the study was based upon clinical criteria, it is not clear whether this result arises from a possible underlying bias. However, adjusting for the number of APOE-ε4 alleles eliminated all the significant neuroanatomical correlations with cognitive response.

A study by Um found that larger left hippocampal total volume and left CA1 volume, measured with a combination of a multi-atlas-based hippocampal subfields segmentation algorithm and a learning-based bias correction technique, were able to predict cognitive response to donepezil at 6 months, with moderate sensitivity and specificity (unfortunately, the authors did not provide optimal cut-off measurements). The authors suggest that this result might be related to the cholinergic deficit in AD, since ACh might be important in maintaining long-term potentiation in CA1 region (Um et al., 2017).

A study by Graff-Radford showed that larger hippocampal volumes in patients with DLB were predictive of cognitive response to AChEI at 1 year. An explanation of this finding could be that such a feature might identify patients with “pure” DLB (i.e., without AD copathology), which implies a more impaired cholinergic deficit compared to AD. Consistently, in an exploratory analysis the authors found larger gray matter volumes in parieto-temporal regions, which are usually involved by AD pathology, to be associated with cognitive response. Hippocampal volumes were calculated after automated segmentation of T1-weighted MRI scans in SPM5, using customized anatomical labeling atlas labels (Graff-Radford et al., 2012).

An analysis of a RCT by Teipel found that larger hippocampal volume, measured after segmentation and normalization of structural MRI scans with SPM8 and VBM8, predicted cognitive response to donepezil in prodromal AD patients (even though the effect was rather small, consisting of 0.70–0.80 points improvement on ADAS-Cog-MCI per year when the hippocampal volume was one standard deviation above the mean) (Teipel et al., 2016). However, given the fact that the diagnosis of MCI did not rely on biomarkers, it is possible that patients with more preserved hippocampus volume were mostly patients with non-AD MCI, which are not expected to deteriorate during the study period. Thus, the findings would reflect the fact that hippocampal volume predicts future conversion to dementia, rather than being a true predictor of treatment response.

#### Other neuroimaging predictors

A CT study by Salib found lower bicaudate span and bicaudate to brain width ratio to be predictive of cognitive response to donepezil at 2 years. The authors believe that their measurements are related to global atrophy, but neurobiological correlates of this finding seem to be lacking (Salib Tony Sheridan Mark Allington, 2001).

The studies on neuroimaging predictors in the last two sections are summarized in Table 9.

**Table 9:** Neuroimaging.

**Reference**	**Population**	**Treatment**	**Diagnosis**	**Response criteria**	**Follow-up**	**Predictor**	**Risk of bias**
Cheng et al. (2015)	353 AD patients	Donepezil, galantamine, rivastigmine	Clinical (probable AD, NINCDS-ADRDA)	Longer TMSE decline-free survival (Survival analysis, using TMSE decline of 3+ points as endpoint)	Survival analysis, median 46.6 months	Lower total MTA score and older age	Low
Connelly et al. (2005a,b)	140 AD patients (160 enrolled)	Donepezil, galantamine, rivastigmine	Clinical (probable AD, NINCDS-ADRDA)	At least two of gain of 2+ points on MMSE, improvement of the combined score IALD and social behavior subscales of NOSGER (or maintenance of maximum score), positive global change as defined as a tripartite agreement amongst doctor, subject, and carer (global impression) at 6 months	3 years (actually 6 months)	Less medial temporal lobe atrophy at baseline	Moderate
Um et al. (2017)	64 AD patients	Donepezil	Clinical (probable AD, NINCDS-ADRDA)	2+ points improvement in MMSE	24 weeks	Higher left hippocampal total volume and left CA1 volume (no cut-offs provided)	Moderate
Csernansky et al. (2005)	39 AD patients (37 completed the study)	Donepezil	Clinical (NINCDS-ADRDA)—doesn't state whether it's probable or possible	Rate of change in ADAS-Cog (primary outcome); CDR, MMSE, NPI (secondary outcome)—Fiala et al. (1998) and Krstic et al. (2012)	2 years	Smaller hippocampal volumes associated with deformation of the lateral and inferomedial surface (inward deformation of the hippocampal surface in proximity to the CA1 subfield and the subiculum) are negative predictor	High
Graff-Radford et al. (2012)	54 DLB patients	Donepezil, galantamine, rivastigmine	Clinical (probable DLB, McKeith et al., 2004)	Reliable change in Dementia Rating Scale: reliable improvement with 9+ points increase in <15 months and 10+ >15 months, reliable decline 6+ decrease in <15 months, and 7+ decrease >15 months, stability in between	1 year	Larger hippocampal volume	Low
Teipel et al. (2016)	215 suspected prodromal AD patients	Donepezil	Clinical (progressive hippocampal amnestic syndrome: free recall ≤ 17 or total recall <40 on FCSRT)	Change in ADAS-Cog-MCI, CDR, TMT-A/B, CVLT	12 months + 6 months OLE	Higher hippocampal volume	Some concerns—Low
Visser et al. (2005)	121 AD patients	Rivastigmine	Clinical (probable AD, NINCDS-ADRDA, and DSM-IV)	2+ points improvement on MMSE or 4+ points improvement on ADAS-Cog	26 weeks	Absence of medial temporal atrophy (Giacobini et al., 2022) in patients receiving low dose of rivastigmine; medial temporal lobe atrophy in general is not a predictor	Low
Salib Tony Sheridan Mark Allington (2001)	59 AD patients	Donepezil 5 mg	Clinical (NINCDS-ADRDA, not stated if possible included)	Unclear: “improvement or cognitive stability,” but non-response is defined as deterioration with MMSE <10 during the study period—probably means that non-responders are those who had to discontinue treatment	2 years	Lower bicaudate span and bicaudate ratio	High

#### Functional neuroimaging

A retrospective study by Mega found that SPECT hypoperfusion of orbitofrontal cortex and dorsolateral prefrontal cortex and hyperperfusion of left anterior medial frontal cortex were predictive of neuropsychiatric response to donepezil at 2 months in AD patients. This finding is consistent with the notion that cholinergic stimulation increases frontal perfusion in AD. Patients with frontal dysfunction would thus benefit from AChEI therapy from a neuropsychiatric point of view (but not from a cognitive perspective) (Mega et al., 2000). A study by Hongo found that lower cerebral blood flow in the right orbito-frontal cortex were predictive of cognitive response to donepezil at 6 months as measured by ADAS-Cog (but not by MMSE), and this effect was significant for the subtests of word recall and orientation. The authors suggested that this association was due to a disruption of the AChE-rich fibers connecting the NB to the orbitofrontal cortex (Hongo et al., 2008).

A study by Tanaka found that cerebral blood flow was preserved in the premotor, parietal, and temporal cortices of neuropsychiatric responders to donepezil compared to non-responders at 3 months. However, the neuropsychiatric improvement in the first group was mainly seen in dysphoria, anxiety, and apathy (Tanaka et al., 2004).

A study by Hanyu found that a reduction in cerebral blood flow in lateral and medial frontal lobe (including the anterior cingulate gyrus) was predictive of poor cognitive response to donepezil at 3 months, while a reduction in cerebral blood flow in the right parietotemporal lobe and in the occipital and medial parietotemporal lobes was associated with good cognitive response. The authors argue that this would probably reflect a detrimental effect of frontal dysfunction on the frontal attentional processing, which is dependent on the cholinergic projections from the NB (Hanyu et al., 2003). The already cited study by Kanetaka showed that less prominent hypoperfusion (also in combination with more severe atrophy of substantia innominate) was associated with cognitive response to donepezil (Kanetaka et al., 2008). The discrepancy between the results by Hongo on one hand and Hanyu and Kanetaka on the other hand may be partially due to different analytical methods (SPM vs. ROI analysis, respectively), dementia severity, treatment duration, and assessment (ADAS-Cog vs. MMSE). However, the reason of these contrasting findings is still not clear (Hanyu et al., 2003; Hongo et al., 2008), but it might be at least partially dependent on other confounding factors not accounted for in these studies.

In a study by Tepmongkol cognitive responders to donepezil at 6 months (measured with the CERAD neuropsychological assessment battery, J-module) showed a relative increase in perfusion in the left superior parietal lobule (Brodman area, BA, 39 and 7), left postcentral gyrus (BA 1) and right superior frontal gyrus (BA6, which is known to be involved in auditory attention to words and effortful retrieval of memory) at 4 h after a single dose of donepezil (which is the drug concentration peak time). When the analysis was restricted to the best seven responders, the only significant area of increased perfusion was found in the right middle occipital gyrus (BA 39). No significant change was detected in non-responders, even though an increased perfusion was noticed in the left BA 39. The occipito-frontal fasciculus connects BA 39 and BA 7, and these areas are known to show hypometabolic changes in pre-dementia states (Tepmongkol et al., 2019). However, as for the majority of functional neuroimaging studies, a convincing and robust explanation of these findings is lacking.

A study by Miettinen found that an increased fMRI activation in the right vs. the left fusiform gyrus on a visual face recognition task after 1 month of low-dose rivastigmine was predictive of cognitive response to rivastigmine at 6 and 12 months with sensitivity of 77.8% and 77.8%, and a specificity of 88.9% and 77.8%, respectively. Interestingly, the authors found that an increased fMRI activation by rivastigmine in areas normally activated by the visual face recognition task (right fusiform gyrus, right prefrontal, bilateral parietal, and ventral occipital areas) was predictive of response, while fMRI activation spreading to areas not associated with the task performance (such as the left fusiform gyrus) was a predictor of poor response. They speculate that a good response to AChEI might be obtained only in the presence of well-functioning brain networks; however, a more in-depth biological explanation is lacking (Miettinen et al., 2015).

The studies included in this section are summarized in Table 10.

**Table 10:** Functional neuroimaging.

**Reference**	**Population**	**Treatment**	**Diagnosis**	**Response criteria**	**Follow-up**	**Predictor**	**Risk of bias**
Mega et al. (2000)	33 AD patients	Donepezil	Clinical (probable or possible AD, NINCDS-ADRDA)	4+ point reduction in total NPI-10 scale (frequency x severity); 4+ increase are non-responders, in-between unchanged behaviorally	8 weeks	Hypoperfusion of orbitofrontal cortex and dorsolateral prefrontal cortex, hyperperfusion of left anterior medial frontal cortex	High
Hongo et al. (2008)	41 AD patients	Donepezil 5 mg	Clinical (probable AD, NINCDS-ADRDA)	Change in ADAS-Cog and MMSE	24 weeks	Lower rCBF values in right orbito-frontal cortex	Low
Tanaka et al. (2004)	70 AD patients	Donepezil 5 mg	Clinical (probable AD, NINCDS-ADRDA)	4+ points improvement in NPI (unchanged: 3—variation in NPI)	12 weeks	Preserved rCBF in the premotor, parietal, and temporal cortices	Moderate
Hanyu et al. (2003)	61 AD patients and 22 controls	Donepezil 5 mg	Clinical (probable AD, NINCDS-ADRDA)	4+ points increase in MMSE	14–16 weeks	Reduction in rCBF in lateral and medial frontal lobe (including the anterior cingulate gyurs) is associated with poor response; reduction in rCBF in right parietotemporal lobe, occipital lobes, medial parietotemporal lobes is associated with good response	Moderate
Kanetaka et al. (2008)	91 AD patients	Donepezil 5 mg	Clinical (probable AD, NINCDS-ADRDA)	4+ points increase in MMSE	Up to 18 weeks	More severe atrophy of substantia innominata and less prominent hypoperfusion in the frontal lobe; MRI × SPECT index (see findings) <1.54	Moderate
Tepmongkol et al. (2019)	25 AD patients (23 included)	Donepezil	Clinical (probable AD, NINCDS-ADRDA)	No deterioration in CERAD J-module	6 months	Hypoperfusion in right subcallosal and orbital gyrus at 4 h after donepezil administration	Moderate
Miettinen et al. (2015)	18 AD patients	Rivastigmine (but could continue with different AChEI)	Clinical (probable AD, NINCDS-ADRDA)	Higher MMSE at 6 and 12 months compared to baseline	12 months	Greater fMRI signal intensity in the right vs. the left fusiform gyrus on a visual face recognition task after 1 month of rivastigmine 1.5 mg ×2; increased fMRI activation by rivastigmine in areas normally activated by the visual face recognition task; fMRI activation spreading to areas not associated with the task performance is a predictor of poor response	Low

#### Neurophysiological predictors

A small study by Adler found that a decrease in EEG theta power compared to pre-treatment 1 week after initiation of rivastigmine was predictive of subsequent short-term memory response at 6 months, together with higher baseline short-term memory performances. A series of works in the 90's attributed increased slow wave activity in AD to the cholinergic deficit (due to reduction of the activating cholinergic projections from the NB). Moreover, short-term memory is known to be dependent on cholinergic function. Thus, the authors suggested that a preservation of cholinergic function demonstrated by higher short-term memory is necessary for response, together with the demonstration of accessibility of the cholinergic system for AChEI, which would be reflected by EEG theta power increase (Adler et al., 2009).

Another placebo-controlled cross-over study by Baakman found that acute decrease of absolute frontal alpha, beta, and theta EEG parameters and relative frontal theta power after a single dose of galantamine 16 mg was predictive of subsequent response at 6 months. In particular, absolute alpha power reduction completely discriminated responders and non-responders (Baakman et al., 2022).

The two studies are included in Supplementary Table S8.

#### Behavioral profile

Two studies by Mega found that worse apathy, depression, disinhibition and irritability (as measured with subscales of NPI) were predictors of neuropsychiatric response to donepezil at 2 months in AD patients (Mega et al., 1999, 2000). To note, a study by Tanaka found that dysphoria, anxiety, and apathy were significantly improved by donepezil at 3 months in neuropsychiatric responders, while the same therapy significantly worsened apathy features in non-responders (Tanaka et al., 2004). A study by Lemstra found a correlation between a cluster of NPI items, including hallucinations, apathy, anxiety, and psychomotor disturbances, and response to rivastigmine at 6 months (Lemstra et al., 2007).

#### Other neuropsychological predictors

An analysis of pooled data from two RCTs on donepezil in patients with DLB showed that impaired scores (1–3) in serial 7's subtractions and figure copying, and delayed recall scores of 1 or more (i.e., patients with typical DLB cognitive impairment pattern, excluding patients with advanced stages of disease) were predictors of cognitive response at 3 months. The authors suggest that this neuropsychological profile would identify patients with greater deficits in attention and visual perception, possibly without coexisting AD pathology (Mori et al., 2016).

A study by Lemstra found that higher fluctuations in the Visual Reaction Time (implied by greater standard deviation) and worse Continuous Performance Test (a measure of sustained attention) at baseline were predictive of response to rivastigmine at 6 months. This was consistent with the idea that cholinergic deficit is related to impairment in attention and concentration. However, responders did not differ from non-responders on the Clinician Assessment of Fluctuations (Lemstra et al., 2007).

A study by Connelly found that higher performances on the Digit Symbol Subtraction test were predictive of response to AChEI at 6 months in AD patients. The authors report that this test is at least in part dependent on cholinergic transmission, so this finding seems quite unexpected in the conceptualization we tried to provide to explain AChEI response, as we would expect a more beneficial effect of AChEI in patients with impaired cholinergic transmission-dependent features (Connelly et al., 2005b).

Taken together, these studies seem to suggest that greater deficits in sustained attention might be predictive of response. The studies on neuropsychological and behavioral predictors are summarized in Supplementary Table S9.

#### Use of artificial intelligence

Although it could not identify any “classical” predictor (i.e., a variable which would show an association with a prespecified outcome), a study by Mecocci found that artificial neural networks, in particular the tomographic analysis with scanning microscopy (TASM), were able to predict response to donepezil with an accuracy of more than 92%, outperforming the best model found by a blinded statistician, which yielded only 81% accuracy (Mecocci et al., 2002). This unconventional finding could raise the possibility that we might use predictive methods along with predictive variables to determine which patients would benefit from AChEI therapy. However, it must be noted that the former does not allow to formulate or confirm hypotheses.

This study is summarized in Supplementary Table S10. Table 11 summarizes the most relevant predictors, categorized by their consistency across studies.

**Table 11:** Summary of the most relevant predictors based on their consistency.

**Evidence**	**Predictors**	**Response**	**Direction of the association**	**Timing of response (months)**	**Section**
Consistent	Hallucinations	Cognitive and global	+	3–5 months	Indicators of cholinergic deficit
	Atrophy of substantia innominata	Cognitive	+	3–12 months	Indicators of cholinergic deficit
	Rivastigmine concentration	Cognitive and global	+	3–7 months	AChEI plasma concentration
	Higher doses of AChEI	Cognitive, functional, global, behavioral	+	24–36 months	Drug dose
	Faster progression	Cognitive, global	+	6 months	Rate of progression
	Low cholesterolemia	Cognitive	+	12 months	Cardiovascular risk factors
	Use of antipsychotics	Cognitive	–	36–48 months	Other drugs and comorbidities
	Higher T4 pre-treatment	Cognitive	+	4–6 months	Thyroid function
	Medial temporal lobe atrophy	Cognitive	–	6–46 months	Medial temporal lobe atrophy
	Apathy, disinhibition, irritability	Behavioral	+	2–3 months	Behavioral profile
	Attention impairment	Cognitive, behavioral	+	3–6 months	Other neuropsychological predictors
Limited	Lower AChE salivary activity	Composite	–	6 months	Indicators of cholinergic deficit
	EEG P3 latency and CRT variability	Cognitive and behavioral	+	6 months	Indicators of cholinergic deficit
	Higher scores on modified vigilance test from MoCA	Cognitive	+	3 months	Indicators of cholinergic deficit
	Low parietal NAA/Cr ratio at baseline	Cognitive	+	3 months	Indicators of cholinergic deficit
	Baseline overall, frontal, and parietal alpha EEG power	Cognitive	+	6 months	Indicators of preserved cholinergic system
	Albumin <4 g/dl (for donepezil)	Cognitive	+	15 months	Serum albumin
	Greater BMI	Composite	–	6 months	Cardiovascular risk factors
	Lower IMT	Cognitive, behavioral, others	+	6 months	Cardiovascular risk factors
	Anticoagulants, gastrointestinal drugs	Cognitive	–	48 months	Other drugs and comorbidities
	Vitamin B12	Cognitive	–	24 months	Vitamin B12
Conflicting	White matter hyperintensities	Cognitive, behavioral, global	±	6–46 months	Indicators of preserved cholinergic system
	BuChE-K variant	Coginitve and global	±	24–36 months	BuChE
	CYP2D6	Cognitive, functional, global	±	3–40 months	Factors involved in drug metabolization
	CYP3A4	Cognitive and global	– or null	12–40 months	FACTORS involved in drug metabolization
	Donepezil concentrations	Cognitive and global	+ or null	3–12 months	AChEI plasma concentration
	Education	Cognitive, global	±	3–48 months	Indicators of cognitive reserve
	Age	Cognitive, functional, global	±	6–48 months	Demographic factors: Age, gender, race
	Gender	Cognitive, global, others	±	6–48 months	Demographic factors: Age, gender, race
	Short-term response	Cognitive, functional	±	9–120 months	Short-term response
	Lower MMSE at baseline	Cognitive, global, functional	±	3–48 months	Measures of cognitive or functional impairment at baseline
	Smoking	Cognitive, composite	±	6–48 months	Cardiovascular risk factors
	Hypertension	Cognitive, global	±	6–12 months	Cardiovascular risk factors
	Antiplatelet therapy	Cognitive	±	36–48 months	Other drugs and comorbidities
	Serum IGF-I levels	Cognitive	±	3–6 months	Serum IGF-1 levels
	Markers of amyloidopathy	Cognitive, behavioral, global	±	6–12 months	Markers of amyloidopathy, CSF biomarkers
	ApoE status	Cognitive, global	± or null	6–36 months	APOE

Relevancy is established also based on predictors availability.

## Conclusions

In the current systematic review we provided a categorization and interpretation of predictors of response to AChEI therapy in neurodegenerative dementias. Although we found great heterogeneity in the definition of response and in the inclusion criteria, some conclusions can still be drawn from the available evidence. Response to AChEI seems to be associated with a more advanced cholinergic deficit (with predictors such as the presence of hallucinations, fluctuating cognition, more severe atrophy of the substantia innominata), and preserved cholinergic neurons (showed for instance by faster alpha on REM sleep EEG, or blood flow increase in the anterior frontal and parietal lobe after donepezil therapy). However, predictors such as white matter hyperintensities in the cholinergic pathways have shown inconsistent results. Response to AChEI according to BuChE genotype seems to be at least partially dependent on the disease stage, with better response in the late stages in the presence of BuChE-K variant, while the role of polymorphisms in other genes related to the cholinergic system, such CHAT, CHRNA7, ACHE, and PON-1, is at least controversial. Factors related to drug availability seem to influence response to AChEI: in particular, low serum albumin for donepezil therapy, CYP2D6 variants associated with reduced enzymatic activity (such as CYP2D6^*^10) and higher drug doses are the most consistent predictors, while it is still controversial whether AChEI concentration influences clinical outcomes. Among other predictors, the most consistent and reproduced ones include faster progression of disease, lower serum cholesterol, less medial temporal lobe atrophy, worse apathy, absence of concomitant diseases, and absence of antipsychotic therapy. Short-term response could be probably regarded as a predictor of subsequent cognitive response, while higher education seems to be a predictor of good response in the short term (months), and a predictor of poor response in the long term (years). Age, gender, baseline cognitive and functional levels, and APOE status relationship with treatment outcome is controversial and warrants further investigations. Other aspects of the cholinergic system, such as CAIP and duplicate α7-nAChR, might be useful to investigate in terms of prediction of AChEI response.

## Data availability statement

The original contributions presented in the study are included in the article/Supplementary material, further inquiries can be directed to the corresponding author/s.

## Author contributions

The process of searching, screening, and appraising the relevant studies have been performed by FP and LT. The manuscript has been drafted by FP and LT, and critically revised and commented upon by all authors. All authors contributed to the article and approved the submitted version.

## Conflict of interest

The authors declare that the research was conducted in the absence of any commercial or financial relationships that could be construed as a potential conflict of interest.

## Publisher's note

All claims expressed in this article are solely those of the authors and do not necessarily represent those of their affiliated organizations, or those of the publisher, the editors and the reviewers. Any product that may be evaluated in this article, or claim that may be made by its manufacturer, is not guaranteed or endorsed by the publisher.

## References

[B1] Adler G. Bektas M. Ko-Inoshishi Y. Tracik F. (2009). Prediction of treatment response to rivastigmine in Parkinson's disease dementia. Alzheimers Dement. 5:252. 10.1016/j.jalz.2009.04.266

[B2] Albani D. Martinelli Boneschi F. Biella G. Giacalone G. Lupoli S. Clerici F. . (2012). Replication study to confirm the role of CYP2D6 polymorphism rs1080985 on donepezil efficacy in Alzheimer's disease patients. J. Alzheimers Dis. 30, 745–749. 10.3233/JAD-2012-112123 22465999

[B3] Angiulli F. Conti E. Zoia C. P. Da Re F. Appollonio I. Ferrarese C. . (2021). Blood-based biomarkers of neuroinflammation in Alzheimer's disease: a central role for periphery? Diagnostics (Basel) 11:1525. 10.3390/diagnostics11091525 34573867 PMC8464786

[B4] Armstrong M. J. Mullins C. D. Gronseth G. S. Gagliardi A. R. (2018). Impact of patient involvement on clinical practice guideline development : a parallel group study. Implement. Sci. 13:55. 10.1186/s13012-018-0745-6 29661195 PMC5902835

[B5] Baakman A. C. Gavan C. Doeselaar L. Kam M. Broekhuizen K. Bajenaru O. . (2022). Acute response to cholinergic challenge predicts long-term response to galantamine treatment in patients with Alzheimer's disease. Br. J. Clin. Pharmacol. 88, 2814–2829. 10.1111/bcp.15206 34964149 PMC9306507

[B6] Babiloni C. Cassetta E. Forno D. Percio D. Ferreri F. Ferri R. . (2006). Donepezil effects on sources of cortical rhythms in mild Alzheimer' s disease : responders vs. non-responders. Neuroimage 31, 1650–1665. 10.1016/j.neuroimage.2006.02.015 16600641

[B7] Ballinger E. Ananth M. Talmage D. A. Role L. W. (2017). Basal forebrain cholinergic circuits and signaling in cognition and cognitive decline. Neuron 91, 1199–1218. 10.1016/j.neuron.2016.09.006 27657448 PMC5036520

[B8] Barbash S. Garfinkel B. P. Maoz R. Simchovitz A. Nadorp B. Guffanti A. . (2017). Alzheimer's brains show inter-related changes in RNA and lipid metabolism. Neurobiol. Dis. 106, 1–13. 10.1016/j.nbd.2017.06.008 28630030 PMC5560656

[B9] Bartus R. T. Dean R. L. Beer B. Lippa A. S. (1982). The cholinergic hypothesis of geriatric memory dysfunction. Science 217, 408–414. 10.1126/science.7046051 7046051

[B10] Birks J. Harvey R. (2018). Donepezil for dementia due to Alzheimer's disease (Review). Cochrane Database Syst. Rev. 2108:CD001190. 10.1002/14651858.CD001190.pub3 12917900

[B11] Boccardi V. Baroni M. Smirne N. Clodomiro A. Ercolani S. Longo A. . (2017). Short-term response is not predictive of long-term response to acetylcholinesterase inhibitors in old age subjects with Alzheimer's disease: a “real world” study. J. Alzheimers Dis. 56, 239–248. 10.3233/JAD-160904 27911323

[B12] Borroni B. Pettenati C. Bordonali T. Akkawi N. Di Luca M. Padovani A. . (2003). Serum cholesterol levels modulate long-term efficacy of cholinesterase inhibitors in Alzheimer disease. Neurosci. Lett. 343, 213–215. 10.1016/S0304-3940(03)00336-7 12770699

[B13] Borroni V. Barrantes F. J. (2021). Homomeric and heteromeric α7 nicotinic acetylcholine receptors in health and some central nervous system diseases. Membranes (Basel) 11:664. 10.3390/membranes11090664 34564481 PMC8465519

[B14] Braga I. L. S. Silva P. N. Furuya T. K. Santos L. C. Pires B. C. Mazzotti D. R. . (2015). Effect of APOE and CHRNA7 genotypes on the cognitive response to cholinesterase inhibitor treatment at different stages of Alzheimer's disease. Am. J. Alzheimers Dis. Other Demen. 30, 139–144. 10.1177/1533317514539540 24951635 PMC10852661

[B15] Brown D. Chisholm J. A. Owens J. Pimlott S. Patterson J. Wyper D. . (2003). Acetylcholine muscarinic receptors and response to anti-cholinesterase therapy in patients with Alzheimer's disease. Eur. J. Nucl. Med. Mol. Imaging 30, 296–300. 10.1007/s00259-002-1028-6 12552349

[B16] Burns A. Yeates A. Akintade L. del Valle M. Zhang R. Y. Schwam E. M. . (2008). Defining treatment response to donepezil in Alzheimer's disease. Drugs Aging 25, 707–714. 10.2165/00002512-200825080-00007 18665662

[B17] Calabria M. Geroldi C. Lussignoli G. Sabbatini F. Zanetti O. (2009). Efficacy of acetyl-cholinesterase-inhibitor (ACHEI) treatment in Alzheimer's disease: a 21-month follow-up “real world” study. Arch. Gerontol. Geriatr. 49, e6–e11. 10.1016/j.archger.2008.07.006 18768226

[B18] Chang Y. S. Wu Y. H. Wang C. J. Tang S. H. Chen H. L. (2018). Higher levels of thyroxine may predict a favorable response to donepezil treatment in patients with Alzheimer disease: a prospective, case-control study. BMC Neurosci. 19:36. 10.1186/s12868-018-0436-x 29929471 PMC6013955

[B19] Chen T. H. Chou M. C. Lai C. L. Wu S. J. Hsu C. L. Yang Y. H. . (2017). Factors affecting therapeutic response to Rivastigmine in Alzheimer's disease patients in Taiwan. Kaohsiung J. Med. Sci. 33, 277–283. 10.1016/j.kjms.2017.04.006 28601231 PMC11916238

[B20] Cheng Y. C. Huang Y. C. Liu H. C. (2018). Effect of apolipoprotein E white-matter change cognitive response to acetylcholinesterase inhibitors in patients with Alzheimer's disease: a systematic review and meta-analysis. Dement. Geriatr. Cogn. Disord. 45, 335–352. 10.1159/000490175 30041236

[B21] Cheng Y. W. Chen T. F. Cheng T. W. Lai Y. M. Hua M. S. Chen Y. F. . (2015). Hippocampal atrophy but not white-matter changes predicts the long-term cognitive response to cholinesterase inhibitors in Alzheimer's disease. Alzheimers Res. Ther. 7:72. 10.1186/s13195-015-0155-9 26592961 PMC4655489

[B22] Chianella C. Gragnaniello D. Maisano Delser P. Visentini M. F. Sette E. Tola M. R. . (2011). BCHE and CYP2D6 genetic variation in Alzheimer's disease patients treated with cholinesterase inhibitors. Eur. J. Clin. Pharmacol. 67, 1147–1157. 10.1007/s00228-011-1064-x 21630031

[B23] Cho H. Huang L. Lee Y. Chan L. Hong C. (2018). Suboptimal baseline serum vitamin B12 is associated with cognitive decline in people with Alzheimer's disease undergoing cholinesterase inhibitor treatment. Front. Neurol. 9:325. 10.3389/fneur.2018.00325 29867734 PMC5954104

[B24] Choi S. H. Kim S. Y. Na H. R. Kim B. K. Yang D. W. Kwon J. C. . (2008). Effect of ApoE genotype on response to donepezil in patients with Alzheimer's disease. Dement. Geriatr. Cogn. Disord. 25, 445–450. 10.1159/000124752 18401173

[B25] Chou P. S. Huang L. C. Hour T. C. Yen C. W. Yang Y. H. (2021). Impact of the CYP2D6 single nucleotide polymorphism on the concentration of and therapeutic response to donepezil in mild-to-moderate Alzheimer's disease. J. Formos. Med. Assoc. 121(1 Pt 2), 409–415. 10.1016/j.jfma.2021.05.026 34120801

[B26] Clarelli F. Mascia E. Santangelo R. Mazzeo S. Giacalone G. Galimberti D. . (2016). CHRNA7 gene and response to cholinesterase inhibitors in an italian cohort of Alzheimer's disease patients. J. Alzheimers Dis. 52, 1203–1208. 10.3233/JAD-160074 27104904

[B27] Connelly P. J. Adams F. Tayar Z. I. Khan F. (2019). Peripheral vascular responses to acetylcholine as a predictive tool for response to cholinesterase inhibitors in Alzheimer's disease. BMC Neurol. 19:88. 10.1186/s12883-019-1316-4 31053120 PMC6500049

[B28] Connelly P. J. Prentice N. P. (2005). Current smoking and response to cholinesterase inhibitor therapy in Alzheimer's disease. Dement. Geriatr. Cogn. Disord. 19, 11–14. 10.1159/000080964 15383739

[B29] Connelly P. J. Prentice N. P. Fowler K. G. (2005a). Hypertension, white matter change and response to cholinesterase inhibitors in Alzheimer's disease. Int. J. Geriatr. Psychiatry 20, 623–628. 10.1002/gps.1331 16021654

[B30] Connelly P. J. Prentice N. P. Fowler K. G. (2005b). Predicting the outcome of cholinesterase inhibitor treatment in Alzheimer's disease. J. Neurol. Neurosurg. Psychiatry 76, 320–324. 10.1136/jnnp.2004.043539 15716519 PMC1739548

[B31] Conti E. Tremolizzo L. Santarone M. E. Tironi M. Radice I. Zoia C. P. . (2016). Donepezil modulates the endogenous immune response: implications for Alzheimer's disease. Hum. Psychopharmacol. Clin. Exp. 31, 296–303. 10.1002/hup.2538 27297668

[B32] Crary J. F. Trojanowski J. Q. Schneider J. A. Abisambra J. F. Abner E. L. Alafuzoff I. . (2014). Primary age-related tauopathy (PART): a common pathology associated with human aging. Acta Neuropathol. 128, 755–766. 10.1007/s00401-014-1349-0 25348064 PMC4257842

[B33] Csernansky J. G. Wang L. Miller J. P. Galvin J. E. Morris J. C. (2005). Neuroanatomical predictors of response to donepezil therapy in patients with dementia. Arch. Neurol. 62, 1718–1722. 10.1001/archneur.62.11.1718 16286546

[B34] De Beaumont L. Pelleieux S. Lamarre-Théroux L. Dea D. Poirier J. (2016). Butyrylcholinesterase K and apolipoprotein E-ϵ4 reduce the age of onset of alzheimer's disease, accelerate cognitive decline, and modulate donepezil response in mild cognitively impaired subjects. J. Alzheimers Dis. 54, 913–922. 10.3233/JAD-160373 27567841

[B35] Devanand D. P. Lentz C. Chunga R. E. Ciarleglio A. Scodes J. M. Andrews H. . (2017). Change in odor identification impairment is associated with improvement with cholinesterase inhibitor treatment in mild cognitive impairment. J. Alzheimers Dis. 60, 1525–1531. 10.3233/JAD-170497 29081417

[B36] Di Lazzaro V. Oliviero A. Pilato F. Saturno E. Dileone M. Marra C. . (2005). Neurophysiological predictors of long term response to AChE inhibitors in AD patients. J. Neurol. Neurosurg. Psychiatry 76, 1064–1069. 10.1136/jnnp.2004.051334 16024879 PMC1739760

[B37] Dos Santos Moraes W. A. Poyares D. R. Guilleminault C. Ramos L. R. Ferreira Bertolucci P. H. Tufik S. . (2006). The effect of donepezil on sleep and REM sleep EEG in patients with Alzheimer disease: a double-blind placebo-controlled study. Sleep 29, 199–205. 10.1093/sleep/29.2.199 16494088

[B38] Droogsma E. Van Asselt D. Diekhuis M. Veeger N. Van Der Hooft C. Deyn D. . (2015). Initial cognitive response to cholinesterase inhibitors and subsequent long-term course in patients with mild Alzheimer's disease. Int. Psychogeriatr. 27, 1323–1333. 10.1017/S1041610215000289 25779465

[B39] Dunn B. Stein P. Cavazzoni P. (2021). Approval of aducanumab for Alzheimer disease — the FDA's perspective. JAMA Intern. Med. 181, 1276–1278. 10.1001/jamainternmed.2021.4607 34254984

[B40] Farlow M. R. Sadowsky C. H. Velting D. M. Meng X. Islam M. Z. (2015). Evaluating response to high-dose 13.3 mg/24 h rivastigmine patch in patients with severe Alzheimer's disease. CNS Neurosci. Ther. 21, 513–519. 10.1111/cns.12385 25675992 PMC6495641

[B41] Fiala M. Zhang L. Gan X. Sherry B. Taub D. Graves M. C. . (1998). Amyloid-β induces chemokine secretion and monocyte migration across a human blood-brain barrier model. Mol. Med. 4, 480–489. 10.1007/BF03401753 9713826 PMC2230332

[B42] Frankfort S. V. Appels B. A. de Boer A. Tulner L. R. van Campen J. P. C. M. Koks C. H. W. . (2007). Identification of responders and reactive domains to rivastigmine in Alzheimer's disease. Pharmacoepidemiol. Drug Saf. 16, 545–551. 10.1002/pds.1345 17109476

[B43] Fritz H. christian J. Ray N Dyrba, M. Sorg C. Teipel S. Grothe M. J. (2018). The corticotopic organization of the human basal forebrain as revealed by regionally selective functional connectivity profiles. Hum. Brain Mapp. 40, 868–878. 10.1002/hbm.24417 30311315 PMC6865372

[B44] Fukui T. Taguchi S. (2005). Do vascular lesions and related risk factors influence responsiveness to donepezil chloride in patients with Alzheimer's disease? Dement. Geriatr. Cogn. Disord. 20, 15–24. 10.1159/000085069 15832031

[B45] Gallacher J. de Reydet de Vulpillieres F. Amzal B. Angehrn Z. Bexelius C. Bintener C. . (2019). Challenges for optimizing real-world evidence in Alzheimer's disease : the ROADMAP project. J. Alzheimers Dis. 67, 495–501. 10.3233/JAD-180370 30584137 PMC6398537

[B46] Gallucci M. Spagnolo P. Aricò M. Grossi E. (2016). Predictors of response to cholinesterase inhibitors treatment of Alzheimer's disease: date mining from the TREDEM registry. J. Alzheimers Dis. 50, 969–979. 10.3233/JAD-150747 26836164

[B47] Gaugler J. E. Kane R. L. Johnston J. A. Sarsour K. (2013). Sensitivity and specificity of diagnostic accuracy in Alzheimer' s disease : a synthesis of existing evidence. Am. J. Alzheimers Dis. Other Demen. 28, 337–347. 10.1177/1533317513488910 23687179 PMC10852625

[B48] Gault J. Robinson M. Berger R. Drebing C. Logel J. Hopkins J. . (1998). Genomic organization and partial duplication of the human α7 neuronal nicotinic acetylcholine receptor gene (CHRNA7). Genomics 52, 173–185. 10.1006/geno.1998.5363 9782083

[B49] Giacobini E. Cuello A. C. Fisher A. (2022). Reimagining cholinergic therapy for Alzheimer's disease. Brain 145, 2250–2275. 10.1093/brain/awac096 35289363

[B50] Graff-Radford J. Boeve B. F. Pedraza O. Ferman T. J. Przybelski S. Lesnick T. G. . (2012). Imaging and acetylcholinesterase inhibitor response in dementia with Lewy bodies. Brain 135(Pt 8), 2470–2477. 10.1093/brain/aws173 22810436 PMC3407425

[B51] Guyatt G. H. Oxman A. D. Vist G. Kunz R. Brozek J. Alonso-coello P. . (2011). GRADE guidelines : 4. Rating the quality of evidence d study limitations (risk of bias). J. Clin. Epidemiol. 64, 407–415. 10.1016/j.jclinepi.2010.07.017 21247734

[B52] Hampel H. Mesulam M. M. Cuello A. C. Farlow M. R. Giacobini E. Grossberg G. T. . (2018). The cholinergic system in the pathophysiology and treatment of Alzheimer's disease. Brain 141, 1917–1933. 10.1093/brain/awy132 29850777 PMC6022632

[B53] HampelH. MesulamM. M. CuelloA. C. KhachaturianA. S. VergalloA. FarlowM. R. . (2019). Revisiting the cholinergic hypothesis in Alzheimer's disease: emerging evidence from translational and clinical research. J. Prev. Alzheimers Dis. 6, 2–15. 10.14283/jpad.2018.4330569080

[B54] Hanyu H. Shimizu T. Tanaka Y. Takasaki M. Koizumi K. Abe K. . (2003). Regional cerebral blood flow patterns and response to donepezil treatment in patients with Alzheimer's disease. Dement. Geriatr. Cogn. Disord. 15, 177–182. 10.1159/000068785 12626849

[B55] Hanyu H. Tanaka Y. Sakurai H. Takasaki M. Abe K. (2002). Atrophy of the substantia innominata on magnetic resonance imaging and response to donepezil treatment in Alzheimer's disease. Neurosci. Lett. 319, 33–36. 10.1016/S0304-3940(01)02507-1 11814647

[B56] Harold D. Macgregor S. Patterson C. E. Hollingworth P. Moore P. Owen M. J. . (2006). A single nucleotide polymorphism in CHAT influences response to acetylcholinesterase inhibitors in Alzheimer's disease. Pharmacogenet. Genomics 16, 75–77. 10.1097/01.fpc.0000189799.88596.04 16424819

[B57] Ho B. L. Kao Y. H. Chou M. C. Yang Y. H. (2016). Cerebral white matter changes on therapeutic response to rivastigmine in Alzheimer's disease. J. Alzheimers Dis. 54, 351–357. 10.3233/JAD-160364 27567838

[B58] Hongo J. Nakaaki S. Shinagawa Y. Murata Y. Sato J. Tatsumi H. . (2008). SPECT-identified neuroanatomical predictor of the cognitive effects of donepezil treatment in patients with Alzheimer's disease. Dement. Geriatr. Cogn. Disord. 26, 556–566. 10.1159/000181148 19066429

[B59] Horikoshi S. Kunii Y. Matsumoto J. Gotoh D. Miura I. Yabe H. . (2020). Does Treatment response with antidementia drugs after 6 months in Alzheimer's disease predict long-term treatment outcome? J. Clin. Psychopharmacol. 40, 195–197. 10.1097/JCP.0000000000001176 32134856

[B60] Jacobo-albavera L. Dom M. Medina-leyte D. J. (2021). The role of the ATP-binding cassette A1 (ABCA1) in human disease. Int. J. Mol. Sci. 22:1593. 10.3390/ijms22041593 33562440 PMC7915494

[B61] Jann M. W. Shirley K. L. Small G. W. (2002). Clinical pharmacokinetics and pharmacodynamics of cholinesterase inhibitors. Clin. Pharmacokinet. 41, 719–739. 10.2165/00003088-200241100-00003 12162759

[B62] Jessen F. Traeber F. Freymann K. Maier W. Schild H. H. Block W. . (2006). Treatment monitoring and response prediction with proton MR spectroscopy in AD. Neurology 67, 528–530. 10.1212/01.wnl.0000228218.68451.31 16894124

[B63] Kanetaka H. Hanyu H. Hirao K. Shimizu S. Sato T. Akai T. . (2008). Prediction of response to donepezil in Alzheimer's disease: combined MRI analysis of the substantia innominata and SPECT measurement of cerebral perfusion. Nucl. Med. Commun. 29, 568–573. 10.1097/MNM.0b013e3282f5e5f4 18458605

[B64] Kapaki E. Paraskevas G. P. Mantzou E. Papapostolou A. Alevizaki M. Vassilopoulos D. . (2006). Thyroid function in patients with Alzheimer disease: implications on response to anticholinesterase treatment. Alzheimer Dis. Assoc. Disord. 20, 242–247. 10.1097/01.wad.0000213856.89613.59 17132968

[B65] Kasuya M. Meguro K. Okamura N. Funaki Y. Ishikawa H. Tanaka N. . (2012). Greater responsiveness to donepezil in Alzheimer patients with higher levels of acetylcholinesterase based on attention task scores and a donepezil PET study. Alzheimers Dis. Assoc. Disord. 26, 113–118. 10.1097/WAD.0b013e3182222bc0 21666432

[B66] Klimkowicz-Mrowiec A. Marona M. Spisak K. Jagiella J. Wolkow P. Szczudlik A. . (2011). Paraoxonase 1 gene polymorphisms do not influence the response to treatment in Alzheimer's disease. Dement. Geriatr. Cogn. Disord. 32, 26–31. 10.1159/000330343 21829028

[B67] Krstic D. Madhusudan A. Doehner J. Vogel P. Notter T. Imhof C. . (2012). Systemic immune challenges trigger and drive Alzheimer-like neuropathology in mice. J. Neuroinflammation 9:151. 10.1186/1742-2094-9-151 22747753 PMC3483167

[B68] Lemstra A. W. Kuiper R. B. Schmand B. Van Gool W. A. (2007). Identification of responders to rivastigmine: a prospective cohort study. Dement. Geriatr. Cogn. Disord. 25, 60–66. 10.1159/000111549 18033962

[B69] Li Y. Hai S. Zhou Y. Br D. (2015). Cholinesterase inhibitors for rarer dementias associated with neurological conditions (Review). Cochrane Database Syst. Rev. 3:CD009444. 10.1002/14651858.CD009444.pub3 25734590 PMC10644993

[B70] Lin Y. T. Chou M. C. Wu S. J. Yang Y. H. (2019). Galantamine plasma concentration and cognitive response in Alzheimer's disease. PeerJ. 7:e6887. 10.7717/peerj.6887 31106076 PMC6500725

[B71] Liu M. Zhang Y. Huo Y. R. Liu S. Liu S. Wang J. . (2014). Influence of the rs1080985 single nucleotide polymorphism of the CYP2D6 gene and APOE polymorphism on the response to donepezil treatment in patients with Alzheimer' s disease in China. Dement. Geriatr. Cogn. Dis. Extra 4, 450–456. 10.1159/000367596 25538729 PMC4264516

[B72] Lu J. Fu J. Zhong Y. Chen P. Yang Q. Zhao Y. . (2016). The roles of apolipoprotein E3 and CYP2D6 (rs1065852) gene polymorphisms in the predictability of responses to individualized therapy with donepezil in Han Chinese patients with Alzheimer's disease. Neurosci. Lett. 614, 43–48. 10.1016/j.neulet.2015.12.062 26768225

[B73] Lu J. Fu J. Zhong Y. Yang Q. Huang J. Li J. . (2018). Association between ABCA1 gene polymorphisms and the therapeutic response to donepezil therapy in Han Chinese patients with Alzheimer's disease. Brain Res. Bull. 140, 1–4. 10.1016/j.brainresbull.2018.03.014 29605487

[B74] Lu J. Wan L. L. Yu Q. Zhong Y. Guo C. (2015). CYP2D6 gene and plasma concentration of S-donepezil as biomarkers to predict and evaluate clinical outcome of donepezil in Chinese Alzheimer's disease patients. Neurodegener. Dis. 15:944. 10.1016/j.jphs.2015.10.010 26603528

[B75] Ma S. L. Tang N. L. S. Wat K. H. Y. Tang J. H. Y. Lau K. H. Law C. B. . (2019). Effect of CYP2D6 and CYP3A4 genotypes on the efficacy of cholinesterase inhibitors in Southern Chinese patients with Alzheimer's disease. Am. J. Alzheimers. Dis. Other Demen. 34, 302–307. 10.1177/1533317519848237 31064198 PMC10852420

[B76] MacGowan S. H. Wilcock G. K. Scott M. (1998). Effect of gender and apolipoprotein E genotype on response to anticholinesterase therapy in Alzheimer's disease. Int. J. Geriatr. Psychiatry 13, 625–630. 10.1002/(sici)1099-1166(199809)13:9<625::aid-gps835>3.0.co;2-2 9777427

[B77] Magliulo L. Dahl M. L. Lombardi G. Fallarini S. Villa L. M. Biolcati A. . (2011). Do CYP3A and ABCB1 genotypes influence the plasma concentration and clinical outcome of donepezil treatment? Eur. J. Clin. Pharmacol. 67, 47–54. 10.1007/s00228-010-0883-5 20931330

[B78] Mahase E. (2021). Aducanumab : European agency rejects Alzheimer's drug over efficacy and safety concerns. BMJ 375:n3127. 10.1136/bmj.n3127 34930757

[B79] Margalit I. Cohen E. Goldberg E. Krause I. (2018). Vitamin B12 deficiency and the role of gender : a cross-sectional study of a large cohort. Ann. Nutr. Metab. 72, 265–271. 10.1159/000488326 29597190

[B80] Maroli A. Di Lascio S. Drufuca L. Cardani S. Setten E. Locati M. . (2019). Effect of donepezil on the expression and responsiveness to LPS of CHRNA7 and CHRFAM7A in macrophages: a possible link to the cholinergic anti-inflammatory pathway. J. Neuroimmunol. 332, 155–166. 10.1016/j.jneuroim.2019.04.012 31048268

[B81] Martinelli-Boneschi F. Giacalone G. Magnani G. Biella G. Coppi E. Santangelo R. . (2013). Pharmacogenomics in Alzheimer's disease: a genome-wide association study of response to cholinesterase inhibitors. Neurobiol. Aging 34, 1711.e7–1711.e13. 10.1016/j.neurobiolaging.2012.12.008 23374588

[B82] Marucci G. Buccioni M. Ben D. D. Lambertucci C. Amenta F. (2020). Efficacy of acetylcholinesterase inhibitors in Alzheimer's disease. Neuropharmacology 190:108352. 10.1016/j.neuropharm.2020.108352 33035532

[B83] McKeith I. G. Wesnes K. A. Perry E. Ferrara R. (2004). Hallucinations predict attentional improvements with rivastigmine in dementia with lewy bodies. Dement. Geriatr. Cogn. Disord. 18, 94–100. 10.1159/000077816 15087584

[B84] Meador K. J. Loring D. W. Adams R. J. Patel B. R. Davis H. C. (1987). Central cholinergic systems and the P3 evoked potential. Int. J. Neurosci. 33, 199–205. 10.3109/00207458708987404 3596949

[B85] Mecocci P. Grossi E. Buscema M. Intraligi M. Savarè R. Rinaldi P. . (2002). Use of artificial networks in clinical trials: a pilot study to predict responsiveness to donepezil in Alzheimer's disease. J. Am. Geriatr. Soc. 50, 1857–1860. 10.1046/j.1532-5415.2002.50516.x 12410907

[B86] Mega M. S. Dinov I. D. Lee L. O'Connor S. M. Masterman D. M. Wilen B. . (2000). Orbital and dorsolateral frontal perfusion defect associated with behavioral response to cholinesterase inhibitor therapy in Alzheimer's disease. J. Neuropsychiatry Clin. Neurosci. 12, 209–218. 10.1176/jnp.12.2.209 11001599

[B87] Mega M. S. Masterman D. M. O'Connor S. M. Barclay T. R. Cummings J. L. (1999). The spectrum of behavioral responses to cholinesterase inhibitor therapy in Alzheimer disease. Arch. Neurol. 56, 1388–1393. 10.1001/archneur.56.11.1388 10555660

[B88] Meng D. Li X. Bauer M. Taylor J. P. Auer D. P. (2018). Altered nucleus basalis connectivity predicts treatment response in mild cognitive impairment. Radiology 289, 775–785. 10.1148/radiol.2018180092 30204076 PMC6283326

[B89] Miettinen P. S. Jauhiainen A. M. Tarkka I. M. Pihlajamäki M. Gröhn H. Niskanen E. . (2015). Long-term response to cholinesterase inhibitor treatment is related to functional MRI response in Alzheimer's disease. Dement. Geriatr. Cogn. Disord. 40, 243–255. 10.1159/000435948 26305064

[B90] Miranda L. F. J. R. Gomes K. B. Silveira J. N. Pianetti G. A. Byrro R. M. D. Peles P. R. H. . (2015). Predictive factors of clinical response to cholinesterase inhibitors in mild and moderate Alzheimer's disease and mixed dementia: a one-year naturalistic study. J. Alzheimers Dis. 45, 609–620. 10.3233/JAD-142148 25589728

[B91] Miranda L. F. J. R. Gomes K. B. Tito P. A. L. Silveira J. N. Pianetti G. A. Byrro R. M. D. . (2017). Clinical response to donepezil in mild and moderate dementia: relationship to drug plasma concentration and CYP2D6 and APOE genetic polymorphisms. J. Alzheimers Dis. 55, 539–549. 10.3233/JAD-160164 27716659

[B92] Modrego P. J. Monleon I. Sarasa M. (2008). The clinical significance of plasmatic amyloid Aβ-40 peptide levels in Alzheimer's disease patients treated with galantamine. Am. J. Alzheimers Dis. Other Demen. 23, 286–290. 10.1177/1533317507313675 18591211 PMC10846209

[B93] Modrego P. J. Rios C. Pérez Trullen J. M. García-Gómara M. J. Errea J. M. (2009). Carotid intima-media thickness as a predictor of response to cholinesterase inhibitors in Alzheimer's disease: an open-label trial. CNS Drugs 23, 253–260. 10.2165/00023210-200923030-00006 19320533

[B94] Mohr F. Krejci E. Zimmermann M. Klein J. (2015). Dysfunctional presynaptic M2 receptors in the presence of chronically high acetylcholine levels: data from the PRiMA knockout mouse. PLoS ONE 10:e0141136. 10.1371/journal.pone.0141136 26506622 PMC4624712

[B95] Molinuevo J. L. Frölich L. Grossberg G. T. Galvin J. E. Cummings J. L. Krahnke T. . (2015). Responder analysis of a randomized comparison of the 13.3 mg/24 h and 9.5 mg/24 h rivastigmine patch. Alzheimers Res. Ther. 7:9. 10.1186/s13195-014-0088-8 25755685 PMC4353453

[B96] Mori E. Ikeda M. Nakagawa M. Miyagishi H. Kosaka K. (2016). Pretreatment cognitive profile likely to benefit from donepezil treatment in dementia with lewy bodies: pooled analyses of two randomized controlled trials. Dement. Geriatr. Cogn. Disord. 42, 58–68. 10.1159/000447586 27537084

[B97] Mossello E. Tonon E. Caleri V. Tilli S. Cantini C. Cavallini M. . (2004). Effectiveness and safety of cholinesterase inhibitors in elderly subjects with Alzheimer's disease: a “real world” study. Arch. Gerontol. Geriatr. Suppl. 9, 297–307. 10.1016/j.archger.2004.04.040 15207427

[B98] Motter J. N. Liu X. Qian M. Cohen H. R. Devanand D. P. (2021). Odor identification impairment and cholinesterase inhibitor treatment in Alzheimer's disease. Alzheimers Dement. 13:e12158. 10.1002/dad2.12158 33816753 PMC8010480

[B99] Nelson P. T. Dickson D. W. Trojanowski J. Q. Jack C. R. Boyle P. A. Arfanakis K. . (2019). Limbic-predominant age-related TDP-43 encephalopathy (LATE): consensus working group report. Brain 142, 1503–1527. 10.1093/brain/awz099 31039256 PMC6536849

[B100] NICE (2011). Donepezil, Galantamine, Rivastigmine and Memantine for the Treatment of *Alzheimer's Disease*. National Institute for Health and Care (NICE).

[B101] Ohnishi T. Sakiyama Y. Okuri Y. Kimura Y. Sugiyama N. Saito T. . (2014). The prediction of response to galantamine treatment in patients with mild to moderate Alzheimer's disease. Curr. Alzheimer Res. 11, 110–118. 10.2174/15672050113106660167 24156269 PMC3979115

[B102] Onofrj M. Thomas A. Iacono D. Luciano A. L. Iorio A. D. (2003). The effects of a cholinesterase inhibitor are prominent in patients with fluctuating cognition: a part 3 study of the main mechanism of cholinesterase inhibitors in dementia. Clin. Neuropharmacol. 26, 239–251. 10.1097/00002826-200309000-00008 14520164

[B103] Ortner M. Stange M. Schneider H. Schröder C. Buerger K. Müller C. . (2020). Therapeutic drug monitoring of rivastigmine and donepezil under consideration of CYP2D6 genotype-dependent metabolism of donepezil. Drug Des. Dev. Ther. 14, 3251–3262. 10.2147/DDDT.S247259 32848364 PMC7431170

[B104] Ouchi Y. Meguro K. Akanuma K. Kato Y. Yamaguchi S. (2015). Normal hearing ability but impaired auditory selective attention associated with prediction of response to donepezil in patients with Alzheimer's disease. Behav. Neurol. 2015:540348. 10.1155/2015/540348 26161001 PMC4487900

[B105] Page M. J. Mckenzie J. E. Bossuyt P. M. Boutron I. Hoffmann C. Mulrow C. D. . (2021). The PRISMA 2020 statement: an updated guideline for reporting systematic reviews systematic reviews and meta-analyses. BMJ 372:n71. 10.1136/bmj.n71 33782057 PMC8005924

[B106] Pakrasi S. Mukaetova-Ladinska E. B. McKeith I. G. O'Brien J. T. (2003). Clinical predictors of response to acetyl cholinesterase inhibitors: experience from routine clinical use in Newcastle. Int. J. Geriatr. Psychiatry 18, 879–886. 10.1002/gps.928 14533120

[B107] Palmqvist S. Minthon L. Wattmo C. Londos E. Hansson O. A. (2010). A Quick Test of cognitive speed is sensitive in detecting early treatment response in Alzheimer disease. Alzheimers. Res. Ther. 2:29. 10.1186/alzrt53 20950460 PMC2983438

[B108] Paroni G. Seripa D. Fontana A. D'Onofrio G. Gravina C. Urbano M. . (2014). FOXO1 locus and acetylcholinesterase inhibitors in elderly patients with Alzheimer's disease. Clin. Interv. Aging. 9, 1783–1791. 10.2147/CIA.S64758 25364236 PMC4211854

[B109] Patterson C. E. Todd S. A. Passmore A. P. (2011). Effect of apolipoprotein e and butyrylcholinesterase genotypes on cognitive response to cholinesterase inhibitor treatment at different stages of Alzheimer's disease. Pharmacogenomics J. 11, 444–450. 10.1038/tpj.2010.61 20644562

[B110] Pelton G. H. Soleimani L. Roose S. P. Tabert M. H. Devanand D. P. (2016). Olfactory deficits predict cognitive improvement on donepezil in patients with depression and cognitive impairment. Alzheimer Dis. Assoc. Disord. 30, 67–69. 10.1097/WAD.0000000000000107 26398910 PMC4764438

[B111] Perera G. Khondoker M. Broadbent M. Breen G. Stewart R. (2014). Factors associated with response to acetylcholinesterase inhibition in dementia: a cohort study from a secondary mental health care case register in london. PLoS ONE 9:e109484. 10.1371/journal.pone.0109484 25411838 PMC4239015

[B112] Pilotto A. Franceschi M. D'Onofrio G. Bizzarro A. Mangialasche F. Cascavilla L. . (2009). Effect of a cyp2d6 polymorphism on the efficacy of donepezil in patients with Alzheimer disease. Neurology 73, 761–767. 10.1212/WNL.0b013e3181b6bbe3 19738170 PMC2739607

[B113] Pola R. Flex A. Ciaburri M. Rovella E. Valiani A. Reali G. . (2005). Responsiveness to cholinesterase inhibitors in Alzheimer's disease: a possible role for the 192 Q/R polymorphism of the PON-1 gene. Neurosci. Lett. 382, 338–341. 10.1016/j.neulet.2005.03.027 15925115

[B114] Raschetti R. Maggini M. Sorrentino G. C. Martini N. Caffari B. Vanacore N. A. . (2005). cohort study of effectiveness of acetylcholinesterase inhibitors in Alzheimer's disease. Eur. J. Clin. Pharmacol. 61, 361–368. 10.1007/s00228-005-0946-1 15912389

[B115] Rey N. L. Wesson D. W. Brundin P. (2018). The olfactory bulb as the entry site for prion-like propagation in neurodegenerative diseases. Neurobiol. Dis. 109, 226–248. 10.1016/j.nbd.2016.12.013 28011307 PMC5972535

[B116] Rigaud A. S. Traykov L. Latour F. Couderc R. Moulin F. Forette F. . (2002). Presence or absence of at least one epsilon 4 allele and gender are not predictive for the response to donepezil treatment in Alzheimer's disease. Pharmacogenetics 12, 415–420. 10.1097/00008571-200207000-00009 12142731

[B117] Rocha-dias P. F. Simao-silva D. P. Suellen S. Piovezan M. R. Souza R. K. M. Darreh-shori T. . (2020). Influence of a genetic variant of CHAT gene over the profile of plasma soluble ChAT in Alzheimer disease. Genet. Mol. Biol. 43:e20190404. 10.1590/1678-4685-gmb-2019-0404 33306773 PMC7783728

[B118] Rota E. Ferrero P. Ursone R. Migliaretti G. (2007). Short term response is predictive of long term response to acetylcholinesterase inhibitors in Alzheimer's disease: a starting point to explore Bayesian approximation in clinical practice. Bioinformation 2, 43–49. 10.6026/97320630002039 18188418 PMC2174418

[B119] Rozzini L. Chilovi V. Bertoletti E. Ghianda D. Conti M. Trabucchi M. . (2008). Serum albumin level interferes with the effect of Donepezil in Alzheimer's disease. Aging Clin. Exp. Res. 20, 509–512. 10.1007/BF03324877 19179833

[B120] Salib Tony Sheridan Mark Allington E. (2001). Ventricular measurements in computed tomography of responders and non-responders to donepezil in the treatment of Alzheimer's disease. Int. J. Psychiatry Clin. Pract. 5, 189–194. 10.1080/136515001317021653 24926752

[B121] Sapien Labs (2018). Understanding Multiscale Entropy. Available online at: https://sapienlabs.org/lab-talk/understanding-multiscale-entropy/

[B122] Sayer R. Law E. Connelly P. J. Breen K. C. (2004). Association of a salivary acetylcholinesterase with Alzheimer's disease and response to cholinesterase inhibitors. Clin. Biochem. 37, 98–104. 10.1016/j.clinbiochem.2003.10.007 14725939

[B123] Scacchi R. Gambina G. Broggio E. Corbo R. M. (2014). Sex and ESR1 genotype may influence the response to treatment with donepezil and rivastigmine in patients with Alzheimer's disease. Int. J. Geriatr. Psychiatry. 29, 610–615. 10.1002/gps.4043 24150894

[B124] Scacchi R. Gambina G. Moretto G. Corbo R. M. (2009). Variability of AChE, BChE, and ChAT genes in the late-onset form of Alzheimer's disease and relationships with response to treatment with donepezil and rivastigmine. Am. J. Med. Genet. B Neuropsychiatr. Genet. 150, 502–507. 10.1002/ajmg.b.30846 18780301

[B125] Selden N. R. Gitelman D. R. Salamon-murayama N. Parrish T. B. Mesulam M. (1998). Trajectories of cholinergic pathways within the cerebral hemispheres of the human brain. Brain 121(Pt 12), 2249–2257. 10.1093/brain/121.12.2249 9874478

[B126] Seripa D. Bizzarro A. Pilotto A. D'Onofrio G. Vecchione G. Gallo A. P. . (2011). Role of cytochrome P4502D6 functional polymorphisms in the efficacy of donepezil in patients with Alzheimer's disease. Pharmacogenet. Genomics 21, 225–230. 10.1097/FPC.0b013e32833f984c 20859244

[B127] Siek G. C. Katz L. S. Fishman E. B. Korosi T. S. Marquis J. K. (1990). Molecular forms of acetylcholinesterase in subcortical areas of normal and Alzheimer disease brain. Biol. Psychiatry 27, 573–580. 10.1016/0006-3223(90)90524-6 2322617

[B128] Sobow T. Flirski M. Liberski P. Kloszewska I. (2007). Plasma Abeta levels as predictors of response to rivastigmine treatment in Alzheimer's disease. Acta Neurobiol. Exp. (Wars). 67, 131–139. 17691220 10.55782/ane-2007-1640

[B129] Sokolow S. Li X. Chen L. Taylor K. D. Rotter J. I. Rissman R. A. . (2017). Deleterious Effect of butyrylcholinesterase K-variant in donepezil treatment of mild cognitive impairment. J. Alzheimers Dis. 56, 229–237. 10.3233/JAD-160562 27911294 PMC5534361

[B130] Sterne J. A. C. Savović J. Page M. J. Elbers R. G. Blencowe N. S. Boutron I. . (2019). RoB 2: a revised tool for assessing risk of bias in randomised trials. BMJ 2019:I4898. 10.1136/bmj.l4898 31462531

[B131] Sumirtanurdin R. Thalib A. Y. Cantona K. Abdulah R. (2019). Effect of genetic polymorphisms on Alzheimer's disease treatment outcomes: an update. Clin. Interv. Aging. 14, 631–642. 10.2147/CIA.S200109 30992661 PMC6445219

[B132] Tanaka M. Namiki C. Thuy D. H. D. Yoshida H. Kawasaki K. Hashikawa K. . (2004). Prediction of psychiatric response to donepezil in patients with mild to moderate Alzheimer's disease. J. Neurol. Sci. 225, 135–141. 10.1016/j.jns.2004.07.009 15465097

[B133] Tanaka Y. Hanyu H. Sakurai H. Takasaki M. Abe K. (2003). Atrophy of the substantia innominata on magnetic resonance imaging predicts response to donepezil treatment in Alzheimer's disease patients. Dement. Geriatr. Cogn. Disord. 16, 119–125. 10.1159/000070998 12826736

[B134] Tei E. Yamamoto H. Watanabe T. Miyazaki A. Nakadate T. Kato N. . (2008). Use of serum insulin-like growth factor-I levels to predict psychiatric non-response to donepezil in patients with Alzheimer's disease. Growth Horm. IGF Res. 18, 47–54. 10.1016/j.ghir.2007.07.006 17714966

[B135] Teipel S. J. Cavedo E. Grothe M. J. Lista S. Galluzzi S. Colliot O. . (2016). Predictors of cognitive decline and treatment response in a clinical trial on suspected prodromal Alzheimer's disease. Neuropharmacology 108, 128–135. 10.1016/j.neuropharm.2016.02.005 26876309

[B136] Tepmongkol S. Hemrungrojn S. Dupont P. Tunvirachaisakul C. Aniwattanapong D. Likitjareon Y. . (2019). Early prediction of donepezil cognitive response in Alzheimer's disease by brain perfusion single photon emission tomography. Brain Imaging Behav. 13, 1665–1673. 10.1007/s11682-019-00182-9 31432319

[B137] Touchon J. Bergman H. Bullock R. Rapatz G. Nagel J. Lane R. . (2006). Response to rivastigmine or donepezil in Alzheimer's patients with symptoms suggestive of concomitant Lewy body pathology. Curr. Med. Res. Opin. 22, 49–59. 10.1185/030079906X80279 16393430

[B138] Tracey K. J. (2009). Reflex control of immunity. Nat. Rev. Immunol. 9, 418–428. 10.1038/nri2566 19461672 PMC4535331

[B139] Tsai P. H. Chang S. C. Liu F. C. Tsao J. Wang Y. H. Lo M. T. A. . (2015). Novel application of multiscale entropy in electroencephalography to predict the efficacy of acetylcholinesterase inhibitor in Alzheimer's disease. Comput. Math. Methods Med. 2015:953868. 10.1155/2015/953868 26120358 PMC4450304

[B140] Ullrich C. Pirchl M. Humpel C. (2010). Hypercholesterolemia in rats impairs the cholinergic system and leads to memory deficits. Mol. Cell. Neurosci. 45, 408–417. 10.1016/j.mcn.2010.08.001 20696249 PMC2977849

[B141] Um Y. H. Kim T. W. Jeong J. H. Seo H. J. Han J. H. Hong S. C. . (2017). Prediction of treatment response to donepezil using automated hippocampal subfields volumes segmentation in patients with mild Alzheimer's disease. Psychiatry Investig. 14, 698–702. 10.4306/pi.2017.14.5.698 29042898 PMC5639141

[B142] Vaknine S. Soreq H. (2020). Central and peripheral anti-inflammatory effects of acetylcholinesterase inhibitors. Neuropharmacology 168:108020. 10.1016/j.neuropharm.2020.108020 32143069

[B143] Villiger Y. Szanto I. Jaconi S. Blanchet C. Buisson B. Krause K. H. . (2002). Expression of an α7 duplicate nicotinic acetylcholine receptor-related protein in human leukocytes. J. Neuroimmunol. 126, 86–98. 10.1016/S0165-5728(02)00057-7 12020960

[B144] Visser P. J. Scheltens P. Pelgrim E. Verhey F. R. J. (2005). Medial temporal lobe atrophy and APOE genotype do not predict cognitive improvement upon treatment with rivastigmine in Alzheimer's disease patients. Dement. Geriatr. Cogn. Disord. 19, 126–133. 10.1159/000082883 15627759

[B145] Wallin A. K. Hansson O. Blennow K. Londos E. Minthon L. (2009). Can CSF biomarkers or pre-treatment progression rate predict response to cholinesterase inhibitor treatment in Alzheimer's disease? Int. J. Geriatr. Psychiatry 24, 638–647. 10.1002/gps.2195 19123199

[B146] Wattmo C. Londos E. Minthon L. (2018). Short-term response to cholinesterase inhibitors in Alzheimer's disease delays time to nursing home placement. Curr. Alzheimer Res. 15, 905–916. 10.2174/1567205015666180507105326 29732972 PMC6174634

[B147] Wattmo C. Wallin Å. K. (2017). Early- versus late-onset Alzheimer's disease in clinical practice: cognitive and global outcomes over 3 years. Alzheimers Res. Ther. 9, 70. 10.1186/s13195-017-0294-2 28859660 PMC5580278

[B148] Wattmo C. Wallin A. K. Londos E. Minthon L. (2011). Predictors of long-term cognitive outcome in Alzheimer's disease. Alzheimers Res. Ther. 3:23. 10.1186/alzrt85 21774798 PMC3226278

[B149] Wattmo C. Wallin A. K. Minthon L. (2012). Functional response to cholinesterase inhibitor therapy in a naturalistic Alzheimer's disease cohort. BMC Neurol. 12:134. 10.1186/1471-2377-12-134 23126532 PMC3534216

[B150] Weng P. H. Chen J. H. Chen T. F. Sun Y. Wen L. L. Yip P. K. . (2013). CHRNA7 polymorphisms and response to cholinesterase inhibitors in Alzheimer's disease. PLoS ONE 8:e84059. 10.1371/journal.pone.0084059 24391883 PMC3877150

[B151] World Health Organization (2021). Dementia. Available online at: https://www.who.int/news-room/fact-sheets/detail/dementia (accessed February 16, 2022).

[B152] Wu M. N. Kao Y. H. Chou P. S. Lin T. C. Kao L. L. Yang Y. H. . (2018). Location of white matter changes and response to donepezil in patients with Alzheimer's disease: a retrospective and observational study. Geriatr. Gerontol. Int. 18, 123–129. 10.1111/ggi.13153 28853195

[B153] Xiao T. Jiao B. Zhang W. Tang B. Shen L. (2016). Effect of the CYP2D6 and APOE polymorphisms on the efficacy of donepezil in patients with Alzheimer's disease: a systematic review and meta-analysis. CNS Drugs 30, 899–907. 10.1007/s40263-016-0356-1 27282366

[B154] Yamagata B. Watanabe T. Tomioka H. Kobayashi H. Nakano Y. Mimura M. . (2010). Preliminary use of insulin-like growth factor-I as a biomarker for sorting high-dose donepezil responders among Japanese patients with Alzheimer's disease. Regul. Pept. 163, 137–142. 10.1016/j.regpep.2010.04.010 20451565

[B155] Yang Y. H. Chen C. H. Chou M. C. Li C. H. Liu C. K. Chen S. H. . (2013). Concentration of donepezil to the cognitive response in alzheimer disease. J. Clin. Psychopharmacol. 33, 351–355. 10.1097/JCP.0b013e31828b5087 23609381

[B156] Yaowaluk T. Senanarong V. Limwongse C. Boonprasert R. Kijsanayotin P. (2019). Influence of CYP2D6, CYP3A5, ABCB1, APOE polymorphisms and nongenetic factors on donepezil treatment in patients with Alzheimer's disease and vascular dementia. Pharmgenomics Pers. Med. 12, 209–224. 10.2147/PGPM.S211259 31564952 PMC6732559

[B157] Yoon H. Myung W. Lim S. W. Kang H. S. Kim S. Won H. H. . (2015). Association of the choline acetyltransferase gene with responsiveness to acetylcholinesterase inhibitors in Alzheimer's disease. Pharmacopsychiatry 48, 111–117. 10.1055/s-0035-1545300 25730470

[B158] Yoshida T. Ha-Kawa S. Yoshimura M. Nobuhara K. Kinoshita T. Sawada S. . (2007). Effectiveness of treatment with donepezil hydrochloride and changes in regional cerebral blood flow in patients with Alzheimer's disease. Ann. Nucl. Med. 21, 257–265. 10.1007/s12149-007-0022-2 17634843

[B159] Zhong Y. Zheng X. Miao Y. Wan L. Yan H. Wang B. . (2013). Effect of CYP2D6^*^10 and APOE polymorphisms on the efficacy of donepezil in patients with Alzheimer's disease. Am. J. Med. Sci. 345, 222–226. 10.1097/MAJ.0b013e318255a8f9 22986607

[B160] Zhu C. W. Livote E. E. Scarmeas N. Albert M. Brandt J. Blacker D. . (2013). Long-term associations between cholinesterase inhibitors and memantine use and health outcomes among patients with Alzheimer' s disease. Alzheimers Dement. 9, 733–740. 10.1016/j.jalz.2012.09.015 23332671 PMC3633652

