# Supplementary material for: Predictors of response to acetylcholinesterase inhibitors in dementia: A systematic review
Source: Front Neurosci. 2022 Sep 20;16:998224. doi: 10.3389/fnins.2022.998224 (PMC9530658; doi:10.3389/fnins.2022.998224)
Supplement: Supplementary file 1 [file Data_Sheet_1.docx]

Supplementary Material

# PRISMA 2020 Main Checklist

| **Topic** | **No.** | **Item** | **Location where item is reported** |
| --- | --- | --- | --- |
| **TITLE** |  |  |  |
| **Title** | 1 | Identify the report as a systematic review. | Title |
| **ABSTRACT** |  |  |  |
| **Abstract** | 2 | See the PRISMA 2020 for Abstracts checklist |  |
| **INTRODUCTION** |  |  |  |
| **Rationale** | 3 | Describe the rationale for the review in the context of existing knowledge. | Section 1.5, lines 355-356 |
| **Objectives** | 4 | Provide an explicit statement of the objective(s) or question(s) the review addresses. | Section 1.5, lines 356-359 |
| **METHODS** |  |  |  |
| **Eligibility criteria** | 5 | Specify the inclusion and exclusion criteria for the review and how studies were grouped for the syntheses. | Methods, lines 368-380 |
| **Information sources** | 6 | Specify all databases, registers, websites, organisations, reference lists and other sources searched or consulted to identify studies. Specify the date when each source was last searched or consulted. | Methods, lines 383-386 |
| **Search strategy** | 7 | Present the full search strategies for all databases, registers and websites, including any filters and limits used. | Methods, lines 387-389 |
| **Selection process** | 8 | Specify the methods used to decide whether a study met the inclusion criteria of the review, including how many reviewers screened each record and each report retrieved, whether they worked independently, and if applicable, details of automation tools used in the process. | Methods, lines 390-394 |
| **Data collection process** | 9 | Specify the methods used to collect data from reports, including how many reviewers collected data from each report, whether they worked independently, any processes for obtaining or confirming data from study investigators, and if applicable, details of automation tools used in the process. | Methods, lines 394-395 |
| **Data items** | 10a | List and define all outcomes for which data were sought. Specify whether all results that were compatible with each outcome domain in each study were sought (e.g. for all measures, time points, analyses), and if not, the methods used to decide which results to collect. | Methods, lines 395-398 |
|  | 10b | List and define all other variables for which data were sought (e.g. participant and intervention characteristics, funding sources). Describe any assumptions made about any missing or unclear information. | Methods, lines 395-398 |
| **Study risk of bias assessment** | 11 | Specify the methods used to assess risk of bias in the included studies, including details of the tool(s) used, how many reviewers assessed each study and whether they worked independently, and if applicable, details of automation tools used in the process. | Methods, lines 399-410 |
| **Effect measures** | 12 | Specify for each outcome the effect measure(s) (e.g. risk ratio, mean difference) used in the synthesis or presentation of results. | Not reported |
| **Synthesis methods** | 13a | Describe the processes used to decide which studies were eligible for each synthesis (e.g. tabulating the study intervention characteristics and comparing against the planned groups for each synthesis (item 5)). | Not reported |
|  | 13b | Describe any methods required to prepare the data for presentation or synthesis, such as handling of missing summary statistics, or data conversions. | Not reported |
|  | 13c | Describe any methods used to tabulate or visually display results of individual studies and syntheses. | Results, lines 411-412 |
|  | 13d | Describe any methods used to synthesize results and provide a rationale for the choice(s). If meta-analysis was performed, describe the model(s), method(s) to identify the presence and extent of statistical heterogeneity, and software package(s) used. | Methods, lines 411-412 |
|  | 13e | Describe any methods used to explore possible causes of heterogeneity among study results (e.g. subgroup analysis, meta-regression). | Not reported |
|  | 13f | Describe any sensitivity analyses conducted to assess robustness of the synthesized results. | Not reported |
| **Reporting bias assessment** | 14 | Describe any methods used to assess risk of bias due to missing results in a synthesis (arising from reporting biases). | Not reported |
| **Certainty assessment** | 15 | Describe any methods used to assess certainty (or confidence) in the body of evidence for an outcome. | Not reported |
| **RESULTS** |  |  |  |
| **Study selection** | 16a | Describe the results of the search and selection process, from the number of records identified in the search to the number of studies included in the review, ideally using a flow diagram. | Results, lines 416-423 |
|  | 16b | Cite studies that might appear to meet the inclusion criteria, but which were excluded, and explain why they were excluded. | Not reported |
| **Study characteristics** | 17 | Cite each included study and present its characteristics. | Tables 2-10 and S1-S10 |
| **Risk of bias in studies** | 18 | Present assessments of risk of bias for each included study. | Tables 2-10 and S1-S10 |
| **Results of individual studies** | 19 | For all outcomes, present, for each study: (a) summary statistics for each group (where appropriate) and (b) an effect estimate and its precision (e.g. confidence/credible interval), ideally using structured tables or plots. | Tables 2-11 and S1-S10 |
| **Results of syntheses** | 20a | For each synthesis, briefly summarise the characteristics and risk of bias among contributing studies. | Not reported |
|  | 20b | Present results of all statistical syntheses conducted. If meta-analysis was done, present for each the summary estimate and its precision (e.g. confidence/credible interval) and measures of statistical heterogeneity. If comparing groups, describe the direction of the effect. | Not reported |
|  | 20c | Present results of all investigations of possible causes of heterogeneity among study results. | Not reported |
|  | 20d | Present results of all sensitivity analyses conducted to assess the robustness of the synthesized results. | Not reported |
| **Reporting biases** | 21 | Present assessments of risk of bias due to missing results (arising from reporting biases) for each synthesis assessed. | Not reported |
| **Certainty of evidence** | 22 | Present assessments of certainty (or confidence) in the body of evidence for each outcome assessed. | Not reported |
| **DISCUSSION** |  |  |  |
| **Discussion** | 23a | Provide a general interpretation of the results in the context of other evidence. | Conclusions, lines 1370-1389 |
|  | 23b | Discuss any limitations of the evidence included in the review. | Conclusions, lines 1368-1370 |
|  | 23c | Discuss any limitations of the review processes used. | Not reported |
|  | 23d | Discuss implications of the results for practice, policy, and future research. | Conclusions, lines 1389-1390 |
| **OTHER INFORMATION** |  |  |  |
| **Registration and protocol** | 24a | Provide registration information for the review, including register name and registration number, or state that the review was not registered. | Not reported |
|  | 24b | Indicate where the review protocol can be accessed, or state that a protocol was not prepared. | Not reported |
|  | 24c | Describe and explain any amendments to information provided at registration or in the protocol. | Not reported |
| **Support** | 25 | Describe sources of financial or non-financial support for the review, and the role of the funders or sponsors in the review. | Funding, lines 1919-1920 |
| **Competing interests** | 26 | Declare any competing interests of review authors. | Conflict of Interest, lines 1912-1913 |
| **Availability of data, code and other materials** | 27 | Report which of the following are publicly available and where they can be found: template data collection forms; data extracted from included studies; data used for all analyses; analytic code; any other materials used in the review. | Data Availability Statement, lines 1922-1923 |

#####

# PRIMSA Abstract Checklist

| **Topic** | **No.** | **Item** | **Reported?** |
| --- | --- | --- | --- |
| **TITLE** |  |  |  |
| **Title** | 1 | Identify the report as a systematic review. | Yes |
| **BACKGROUND** |  |  |  |
| **Objectives** | 2 | Provide an explicit statement of the main objective(s) or question(s) the review addresses. | Yes |
| **METHODS** |  |  |  |
| **Eligibility criteria** | 3 | Specify the inclusion and exclusion criteria for the review. | Yes |
| **Information sources** | 4 | Specify the information sources (e.g. databases, registers) used to identify studies and the date when each was last searched. | Yes |
| **Risk of bias** | 5 | Specify the methods used to assess risk of bias in the included studies. | No |
| **Synthesis of results** | 6 | Specify the methods used to present and synthesize results. | Yes |
| **RESULTS** |  |  |  |
| **Included studies** | 7 | Give the total number of included studies and participants and summarise relevant characteristics of studies. | Yes |
| **Synthesis of results** | 8 | Present results for main outcomes, preferably indicating the number of included studies and participants for each. If meta-analysis was done, report the summary estimate and confidence/credible interval. If comparing groups, indicate the direction of the effect (i.e. which group is favoured). | No |
| **DISCUSSION** |  |  |  |
| **Limitations of evidence** | 9 | Provide a brief summary of the limitations of the evidence included in the review (e.g. study risk of bias, inconsistency and imprecision). | Yes |
| **Interpretation** | 10 | Provide a general interpretation of the results and important implications. | Yes |
| **OTHER** |  |  |  |
| **Funding** | 11 | Specify the primary source of funding for the review. | No |
| **Registration** | 12 | Provide the register name and registration number. | No |

From: Page MJ, McKenzie JE, Bossuyt PM, Boutron I, Hoffmann TC, Mulrow CD, et al. The PRISMA 2020 statement: an updated guideline for reporting systematic reviews. MetaArXiv. 2020, September 14. DOI: 10.31222/osf.io/v7gm2. For more information, visit: [www.prisma-statement.org](http://www.prisma-statement.org)

# Studies not described in the main text

**4.1.3 BuChE**

A post-hoc analysis of a RCT by Bullock found that patients carrying BuChE wildtype alleles had a better response to rivastigmine compared to donepezil, but only in the subgroup younger than 75; however, the result may be biased by the fact that more patients in the rivastigmine group dropped-out during the titration phase, which might have resulted in a lower magnitude on change in the ITT-LOCF analysis[1].

A post-hoc analysis of a RCT by O’Brien showed that patients with DLB carrying mutations which lower BuChE activity (namely, patients with heterozygous Asp70Gly and homozygous Ala539Thr mutations) had no attentional response to rivastigmine at 20 weeks, while BuChE wildtype and heterozygous patients showed at least partial responses; however, the same mutations resulted in a less compromised attention compared to BuChE wildtype carriers at baseline, in the earlier stages of disease, possibly implying a ceiling effect[2]**.**

**4.1.4 CHAT**

A study by Scacchi found that the rs2177369 SNP of CHAT was not a predictor of response to rivastigmine or donepezil[3].

**4.2.3 AChEI plasma concentrations**

A study by Chen concluded that rivastigmine concentration was associated with cognitive response at 6 months, but the confidence interval of the provided OR included the point of no effect[4].

Another study by Lu found that ratio between the concentration of the S enantiomer of donepezil and the received dose was significantly higher in responders at 3 months in Chinese Han patients with AD; the effect was more evident in patients with CYP2D6 rs1065852 SNP *10/10. However, the authors did not provide established diagnostic criteria for patients’ inclusion[5].

**4.2.5 Drug type**

A retrospective analysis of a RCT by Touchon found that patients on rivastigmine exhibited better cognitive and functional response at 2 years compared to patients on donepezil; however, these findings were only observed in the intention-to-treat population, with last observation carried forward imputation for missing data, and were not confirmed in the observed cases population[6].

**4.3.2 Demographic factors: age, gender, race**

A post-hoc analysis of a RCT by Bullock found that patients younger than 75 responded better to rivastigmine than donepezil; however, in the same study, more patients on rivastigmine, compared to donepezil, discontinued the drug due to adverse events during the titration phase, which may have resulted in an overestimation of the drug effects in the ITT-LOCF analysis. Moreover, this effect was only seen in BuChE wildtype subjects, and no direct comparison between younger and older patients seems to have been performed[1]

**4.3.3 Rate of progression**

A study by Farlow, retrospectively examining the open-label extension of a RCT on rivastigmine vs placebo, suggested that rapid progressors (i.e. patients who experienced a 4 or more points deterioration on the ADAS-Cog or at least 10% worsening on Progressive Deterioration Scale during the first 26 weeks – the RCT phase) had a better response to rivastigmine in the subsequent 26 weeks both on ADAS-Cog and PDS. However, a rapid progression could have been hypothetically the result of placebo treatment, while slow progression could have been due to rivastigmine: in this scenario, subsequent rivastigmine therapy would have showed greater effects in the “naïve” group, coincident with rapid progressors, whereas the already “treated” group would have seen the well-described decline in response after 6 months of therapy. Indeed, the study suggested that after 6 months slow progressors became faster progressors, and vice versa. Since the authors did not provide data regarding the association between progression and previous randomization group, it is not possible to exclude such an obvious bias[7].

A study by Calabria concluded that short-term cognitive improvement at 3 months was a predictor of subsequent response at 21 months; however, the significant drop-out rate of almost 50% could have influenced the results[8]. A study by Rota confirmed the same association only in patients with milder dementia (MMSE > 18), with an even greater drop-out rate; the same work found that improvement in ADL and IADL at 3 months was a predictor of functional improvement at 15 months[9].

**4.3.4 Short-term response**

A retrospective study by Droogsma found that cognitive response at 6 months was not a predictor of subsequent response in a more than 3-years follow-up; interestingly, while non-responders had a lower MMSE at baseline, they exhibited a statistically significantly slower rate of cognitive decline. However, the difference in progression between non-responders and responders (0.9 vs 1.2 points/year) may be completely irrelevant from a clinical point of view[10].

A post-hoc analysis of a RCT by Farlow showed that “improvement, no change or minimal worsening” at 8 weeks on the ADAS-CGIC, corresponding to a score of 5, had a sensitivity of 59% and a specificity of 81% in predicting response at 24 weeks, defined as “improvement or no change” on ADAS-CGIC, which would imply that short-term response correlates with long-term response[11]. However, the study only included patients with severe dementia, in which the discriminative power of ADAS-CGIC might be limited.

**4.3.5** **Measures of cognitive or functional impairment at baseline**

A study by Gallucci found that MMSE > 20, CDR between 0.5-1 and higher ADL (and a lower degree of cortical and subcortical atrophy) were predictive of cognitive response to AChEI, implying that more preserved subjects responded better; however, the same study identified also living with assistance as a predictor[12]. The fact that the paper used artificial intelligence to find several possible associations, instead of a priori hypotheses to be tested, could at least partially explain these contradictory results.

**4.3.12 Markers of amyloidopathy**

A retrospective study by Graff-Radford on patients with DLB suggested that negative PiB-PET might a predictor of cognitive response to AChEI at 1 year; however, only 7 patients underwent such imaging, and only a descriptive analysis was provided[13]. These findings suggest that patients with less Alzheimer (co-)pathology would benefit more from AChEI therapy, which is consistent with the fact that subjects with DLB tend to have greater cholinergic deficit and lower choline acetyltransferase activity compared to AD patients.

**4.3.14 APOE**

Most studies did not find an effect of APOE status on cognitive response, and they will not be discussed further[1,13–33].

The study by Blesa found a differential effect of APOE-ε4 in psychiatric response to rivastigmine in AD patients at 6 months, with greater efficacy on anxiety-related behavioral symptoms in non-carriers, and on irritability in carriers, as measured by NPI subitems[30]; however, total NPI was not affected by APOE status, and these results should be interpreted with caution.

**4.3.23 Other neuropsychological predictors**

In a rather complicated prospective randomized study, Kuzmickienė found that scores in a few subtests of the Cambridge Neuropsychological Test Automated Battery (CANTAB) were predictive of cognitive response to donepezil at 4 months (measured with MMSE), namely improvement in Paired associate learning (PAL) after a single dose of donepezil. The authors report that PAL test seems to be more sensitive to cholinergic stimulation than other tests in the CANTAB battery. Other tests scores were considered predictive of cognitive response at 4 months (measured by CANTAB battery), namely improvement in Pattern recognition memory (PRM) a test of visual recognition memory), Spatial working memory after a single dose of donepezil, and PAL, PRM and Choice reaction Time scores at baseline[34]. However, the fact that these findings originated from several different models makes their significance difficult to judge.

A study by Saumier found that better performances at baseline in the Clock Drawing test, the Boston Naming test and a tracking speed test were predictive of cognitive and global response at 6 months in Canadian AD patients. Curiously, scores in neuropsychological tests evaluating attention were not predictive of response; however, responders were significatively less impaired than non-responders in Psycholinguistical Assessment of Language Picture Naming test for pictures depicting living things. Thus, the author suggest that more preserved visual-spatial abilities and lexical-semantical knowledge might identify a subset of patients more likely to respond to donepezil[35]. However, a biological explanation of these findings was not provided.

**References**

1. Bullock R, Bergman H, Touchon J, Gambina G, He Y, Nagel J, et al. Effect of age on response to rivastigmine or donepezil in patients with Alzheimer’s disease. Curr Med Res Opin. 2006;22(3):483–94. doi: 10.1185/030079906X89685

2. O’Brien KK, Saxby BK, Ballard CG, Grace J, Harrington F, Ford GA, et al. Regulation of attention and response to therapy in dementia by butyrylcholinesterase. Pharmacogenetics. 2003;13(4):231–9. doi: 10.1097/00008571-200304000-00008

3. Scacchi R, Gambina G, Moretto G, Corbo RM. Variability of AChE, BChE, and ChAT genes in the late-onset form of Alzheimer’s disease and relationships with response to treatment with donepezil and rivastigmine. Am J Med Genet Part B Neuropsychiatr Genet. 2009;150(4):502–7. doi: 10.1002/ajmg.b.30846

4. Chen TH, Chou MC, Lai CL, Wu SJ, Hsu CL, Yang YH. Factors affecting therapeutic response to Rivastigmine in Alzheimer’s disease patients in Taiwan. Kaohsiung J Med Sci [Internet]. 2017;33(6):277–83. doi: 10.1016/j.kjms.2017.04.006

5. Lu J, Fu J, Zhong Y, Chen P, Yang Q, Zhao Y, et al. The roles of apolipoprotein E3 and CYP2D6 (rs1065852) gene polymorphisms in the predictability of responses to individualized therapy with donepezil in Han Chinese patients with Alzheimer’s disease. Neurosci Lett. 2016 Feb;614:43–8. doi: 10.1016/j.neulet.2015.12.062

6. Touchon J, Bergman H, Bullock R, Rapatz G, Nagel J, Lane R. Response to rivastigmine or donepezil in Alzheimer’s patients with symptoms suggestive of concomitant Lewy body pathology. Curr Med Res Opin. 2006;22(1):49–59. doi: 10.1185/030079906X80279

7. Farlow MR, Hake A, Messina J, Hartman R, Veach J, Anand R. Response of patients with Alzheimer's disease to rivastigmine treatment is predicted by the rate of disease progression. Arch Neurol. 2001 Mar;58(3):417–22. doi: 10.1001/archneur.58.3.417

8. Calabria M, Geroldi C, Lussignoli G, Sabbatini F, Zanetti O. Efficacy of acetyl-cholinesterase-inhibitor (ACHEI) treatment in Alzheimer’s disease: a 21-month follow-up “real world” study. Arch Gerontol Geriatr. 2009;49(1):e6-11. doi: 10.1016/j.archger.2008.07.006

9. Rota E, Ferrero P, Ursone R, Migliaretti G. Short term response is predictive of long term response to acetylcholinesterase inhibitors in Alzheimer’s disease: a starting point to explore Bayesian approximation in clinical practice. Bioinformation. 2007 Aug;2(2):43–9. doi: 10.6026/97320630002039

10. Droogsma E, Van Asselt D, Diekhuis M, Veeger N, Van Der Hooft C, De Deyn PP. Initial cognitive response to cholinesterase inhibitors and subsequent long-term course in patients with mild Alzheimer’s disease. Int Psychogeriatrics. 2015;27(8):1323–33. doi: 10.1017/S1041610215000289

11. Farlow MR, Sadowsky CH, Velting DM, Meng X, Islam MZ. Evaluating Response to High-Dose 13.3 mg/24 h Rivastigmine Patch in Patients with Severe Alzheimer’s Disease. CNS Neurosci Ther. 2015 Jun;21(6):513–9. doi: 10.1111/cns.12385

12. Gallucci M, Spagnolo P, Aricò M, Grossi E. Predictors of Response to Cholinesterase Inhibitors Treatment of Alzheimer’s Disease: Date Mining from the TREDEM Registry. J Alzheimers Dis. 2016;50(4):969–79. doi: 10.3233/JAD-150747

13. Graff-Radford J, Boeve BF, Pedraza O, Ferman TJ, Przybelski S, Lesnick TG, et al. Imaging and acetylcholinesterase inhibitor response in dementia with Lewy bodies. Brain. 2012 Aug;135(Pt 8):2470–7. doi: 10.1093/brain/aws173

14. Waring JF, Tang Q, Robieson WZ, King DP, Das U, Dubow J, et al. APOE-ε4 Carrier Status and Donepezil Response in Patients with Alzheimer’s Disease. J Alzheimer’s Dis. 2015;47(1):137–48. doi: 10.3233/JAD-142589

15. Aerssens J, Raeymaekers P, Lilienfeld S, Geerts H, Konings F, Parys W. APOE genotype: No influence on galantamine treatment efficacy nor on rate of decline in Alzheimer’s disease. Dement Geriatr Cogn Disord. 2001;12(2):69–77. doi: 10.1159/000051238

16. Ho BL, Kao YH, Chou MC, Yang YH. Cerebral white matter changes on therapeutic response to rivastigmine in Alzheimer’s disease. J Alzheimer’s Dis. 2016;54(1):351–7. doi: 10.3233/JAD-160364

17. Sobow T, Flirski M, Liberski P, Kloszewska I. Plasma Abeta levels as predictors of response to rivastigmine treatment in Alzheimer’s disease. Acta Neurobiol Exp (Wars). 2007;67(2):131–9.

18. Miranda LFJR, Gomes KB, Silveira JN, Pianetti GA, Byrro RMD, Peles PRH, et al. Predictive Factors of Clinical Response to Cholinesterase Inhibitors in Mild and Moderate Alzheimer’s Disease and Mixed Dementia: A One-Year Naturalistic Study. J Alzheimer’s Dis. 2015;45(2):609–20. doi: 10.3233/JAD-142148

19. Pilotto A, Franceschi M, D’Onofrio G, Bizzarro A, Mangialasche F, Cascavilla L, et al. Effect of a cyp2d6 polymorphism on the efficacy of donepezil in patients with alzheimer disease. Neurology. 2009;73(10):761–7. doi: 10.1212/WNL.0b013e3181b6bbe3

20. Clarelli F, Mascia E, Santangelo R, Mazzeo S, Giacalone G, Galimberti D, et al. CHRNA7 Gene and Response to Cholinesterase Inhibitors in an Italian Cohort of Alzheimer’s Disease Patients. J Alzheimer’s Dis. 2016;52(4):1203–8. doi: 10.3233/JAD-160074

21. Liu M, Zhang Y, Huo YR, Liu S, Liu S, Wang J, et al. Influence of the rs1080985 Single Nucleotide Polymorphism of the CYP2D6 Gene and APOE Polymorphism on the Response to Donepezil Treatment in Patients with Alzheimer ’ s Disease in China. 2014;300060:450–6. doi: 10.1159/000367596

22. Miranda LFJR, Gomes KB, Tito PAL, Silveira JN, Pianetti GA, Byrro RMD, et al. Clinical Response to Donepezil in Mild and Moderate Dementia: Relationship to Drug Plasma Concentration and CYP2D6 and APOE Genetic Polymorphisms. J Alzheimer’s Dis. 2017;55(2):539–49. doi: 10.3233/JAD-160164

23. Klimkowicz-Mrowiec A, Marona M, Spisak K, Jagiella J, Wolkow P, Szczudlik A, et al. Paraoxonase 1 gene polymorphisms do not influence the response to treatment in Alzheimer’s disease. Dement Geriatr Cogn Disord. 2011;32(1):26–31. doi: 10.1159/000330343

24. Lin YT, Chou MC, Wu SJ, Yang YH. Galantamine plasma concentration and cognitive response in Alzheimer’s disease. PeerJ. 2019;2019(5). doi: 10.7717/peerj.6887

25. Zhong Y, Zheng X, Miao Y, Wan L, Yan H, Wang B. Effect of CYP2D6*10 and APOE polymorphisms on the efficacy of donepezil in patients with Alzheimer’s disease. Am J Med Sci [Internet]. 2013;345(3):222–6. doi: 10.1097/MAJ.0b013e318255a8f9

26. Visser PJ, Scheltens P, Pelgrim E, Verhey FRJ. Medial temporal lobe atrophy and APOE genotype do not predict cognitive improvement upon treatment with rivastigmine in Alzheimer’s disease patients. Dement Geriatr Cogn Disord. 2005;19(2–3):126–33. doi: 10.1159/000082883

27. Rigaud AS, Traykov L, Latour F, Couderc R, Moulin F, Forette F. Presence or absence of at least one epsilon 4 allele and gender are not predictive for the response to donepezil treatment in Alzheimer’s disease. Pharmacogenetics. 2002 Jul;12(5):415–20. doi: 10.1097/00008571-200207000-00009

28. MacGowan SH, Wilcock GK, Scott M. Effect of gender and apolipoprotein E genotype on response to anticholinesterase therapy in Alzheimer’s disease. Int J Geriatr Psychiatry. 1998 Sep;13(9):625–30. doi: 10.1002/(sici)1099-1166(199809)13:9<625::aid-gps835>3.0.co;2-2

29. Wallin AK, Hansson O, Blennow K, Londos E, Minthon L. Can CSF biomarkers or pre-treatment progression rate predict response to cholinesterase inhibitor treatment in Alzheimer’s disease? Int J Geriatr Psychiatry. 2009 Jun;24(6):638–47. doi: 10.1002/gps.2195

30. Blesa R, Aguilar M, Casanova JP, Boada M, Martínez S, Alom J, et al. Relationship between the efficacy of rivastigmine and apolipoprotein E (ε4) in patients with mild to moderately severe Alzheimer disease. Alzheimer Dis Assoc Disord. 2006;20(4):248–54. doi: 10.1097/01.wad.0000213880.93665.c7

31. De Beaumont L, Pelleieux S, Lamarre-Théroux L, Dea D, Poirier J. Butyrylcholinesterase K and Apolipoprotein E-ϵ4 Reduce the Age of Onset of Alzheimer’s Disease, Accelerate Cognitive Decline, and Modulate Donepezil Response in Mild Cognitively Impaired Subjects. J Alzheimer’s Dis. 2016;54(3):913–22. doi: 10.3233/JAD-160373

32. Farlow M, Lane R, Kudaravalli S, He Y. Differential qualitative responses to rivastigmine in APOE E4 carriers and noncarriers. Pharmacogenomics J. 2004;4(5):332–5. doi: 10.1038/sj.tpj.6500267

33. Ma SL, Tang NLS, Wat KHY, Tang JHY, Lau KH, Law CB, et al. Effect of CYP2D6 and CYP3A4 Genotypes on the Efficacy of Cholinesterase Inhibitors in Southern Chinese Patients With Alzheimer’s Disease. Am J Alzheimers Dis Other Demen. 2019;34(5):302–7. doi: 10.1177/1533317519848237

34. Kuzmickienė J, Kaubrys G. Cognitive results of CANTAB tests and their change due to the first dose of donepezil may predict treatment efficacy in alzheimer disease. Med Sci Monit. 2015;21:3887–99. doi: 10.12659/MSM.896327

35. Saumier D, Murtha S, Bergman H, Phillips N, Whitehead V, Chertkow H. Cognitive predictors of donepezil therapy response in alzheimer disease. Dement Geriatr Cogn Disord. 2007;24(1):28–35. doi: 10.1159/000102569

# Supplementary Tables

Table S1. Genetic of the cholinergic system

| Reference | Population | Treatment | Diagnosis | Response criteria | Follow-up | Predictor | Risk of bias |
| --- | --- | --- | --- | --- | --- | --- | --- |
| Scacchi 2008 | 171 AD patients | Rivastigmine, donepezil | Clinical (probable AD, NINCDS-ADRDA and DSM-IV) | Mean values on MMSE among four recording times (no clear definition of response) | 15 months | A/A genotype of AChE rs2571598 SNP (only for rivastigmine); BChErs1803274 (the so-called K-variant),BChE rs1355534, and ChAT rs2177369 are not a predictor | High |
| Bullock 2006 | 994 AD patients (moderately-severe), 578 completed the study | Rivastigmine, donepezil | DSM-IV criteria and NINCDS-ADRDA | Severe impairment battery, no clear definition. | 2 years | Age younger than 75 and BuChE wt (for treatment with R) | Some concerns – moderate |
| Sokolow 2016 | 574 MCI patients | Donepezil | Clinical (amnestic MCI of a degenerative nature, logical memory delayed-recall score 1.5-2 SD below an education-adjusted norm, CDR 0.5, MMSE 24-30, Petersen) | Change of MMSE and CDR-SB | 3 years | BChE-K variant is a predictor of poor response in MCI patients who are ApoE e4 carriers | Some concerns – moderate |
| De Beaumont 2016 | 128 MCI patients | Donepezil | Clinical (aMCI subjects according to ref. 46) | ADAS-Cog change | 3 years | Women with BChE-K* | Moderate |
| Patterson 2011 | 165 AD patients (81 completed the whole study) | Rivastigmine, donepezil, galantamine | Clinical (probable AD, NINCDS-ADRDA and DSM-IV) | Improvement in MMSE at 3-9 months (early cognitive response); no decline in MMSE at 15-24 months (late cognitive response) | Up to 24 months | APOE e4 in MMSE>21, BCHE-K in MMSE <= 15 | Moderate |
| O'Brien 2002 | 51 DLB patients | Rivastigmine | Clinical (DLB, McKeith) | Change in SRT and CRT | 20 weeks | Inconclusive results | Some concerns – moderate |
| Harold 2006 | 121 AD patients | Rivastigmine, donepezil, galantamine | Not stated (presumably probable AD - NINCDS-ADRDA, ref. 19) | Rate of decline in MMSE (points/year) | Up to 24 months (average 15 months) | rs733722 TT allele SNP of CHAT | High |
| Yoon 2015 | 158 AD patients | Rivastigmine, donepezil, galantamine | Clinical (probable AD, NINCDS-ADRDA) | No deterioration in K-MMSE | 26 weeks | rs2177370 and rs3793790 SNPs of CHAT associated with response; haplotype CT of rs11191187-rs2177370 of CHAT is a predictor, haplotype CC is a predictor of poor response | Low |
| Braga 2014 | 177 AD patients (at 6 months), 147 AD patients (at 2 years) | Rivastigmine, donepezil, galantamine | Clinical (probable AD, NINCDS-ADRDA) | MMSE improvement or no deterioration | 2 years | T allele of rs6494223 polymorphism of CHRNA7 and ApoE e4 non carriers | Low |
| Weng 2013 | 204 AD patients | Rivastigmine, donepezil, galantamine | Clinical (probable AD, NINCDS-ADRDA) | 2+ points improvement in MMSE | 6 months | G allele of rs8024987 SNP of CHRNA7 in women (especially taking galantamine); haplotype GG of rs8024987 and rs885071 SNPs of CHRNA7 in women (especially taking galantamine) | Low |
| Clarelli 2016 | 169 AD patients (a subset with MMSE > 19 was selected to be consistent with Braga2014) | Rivastigmine, donepezil, galantamine | Clinical (probable AD, NINCDS-ADRDA) ref.11 | Non-worsening of MMSE (as in Braga2015) and improvement of 2+ points in MMSE (as in Weng) | 1 year | CHRNA7 rs6494223 and rs8024987 are not predictors | Low |
| Pola 2005 | 73 AD patients | Rivastigmine, donepezil | Clinical (probable AD, NINCDS-ADRDA) | 2+ points improvement on MMSE, no deterioration of ADL and IADL | 9.1 +- 2.6 months | R allele of residue 192 polymorphism of PON-1; QQ genotype is a predictor of poor response | Moderate |
| Klimkowicz-Mrowiec 2011 | 101 AD patients | Rivastigmine, donepezil | Clinical (probable AD, NINCDS-ADRDA) | No deterioration in MMSE and CDT, global improvement on IADL | 9 months | PON1 SNPs rs662, rs854560 and rs705381 are not a predictor | Moderate |

Table S2. CYP2D6

| Reference | Population | Treatment | Diagnosis | Response criteria | Follow-up | Predictor | Risk of bias |
| --- | --- | --- | --- | --- | --- | --- | --- |
| Lu 2015 | 77 AD patients | Donepezil | Not stated | No deterioration in MMSE | At least 3 months | Higher concentration of S-Donepezil (in patients with CYP2D6*10/10 on rs1065852 SNP) | High |
| Lu 2016 | 85 AD patients | Donepezil | Not stated | No deterioration in MMSE | At least 3 months | APOE e3 non-carrier and CYP2D6*10/10 on rs1065852 SNP | High |
| Ma 2019 | 174 AD patients (with patients treated with R as a control group) | Donepezil, galantamine, rivastigmine | Clinical (probable and possible AD, NINCDS-ADRDA) | No deterioration in ADAS-Cog and MMSE | 1 year | CYP2D6*10 carriers | High |
| Zhong 2012 | 110 AD patients (96 completed the study) | Donepezil | Clinical (probable AD, NINCDS-ADRDA) | 2+ points improvement in MMSE (but also "no deterioration") | 6 months | CYP2D6*10; APOE is not a predictor | Moderate |
| Magliulo 2011 | 54 AD patients | Donepezil | Clinical (probable AD, NINCDS-ADRDA) | Not clearly stated (change in CIBIC-plus or in MMSE) | Up to 40 months (median 9 months) | Heterozygous CYP2D6 extensive metabolizers (CYP2D6*1 carriers) predicts better response than homozygous CYP2D6 extensive metabolizers (CYP2D6*1/*1) | High |
| Seripa 2011 | 57 AD patients | Donepezil | Clinical (probable AD, NINCDS-ADRDA) | No deterioration in ADAS-Cog and MMSE, improvement on ADL or IADL | 6 months | Mutations of CYP2D6 associated with absent or decreased enzyme activity | Moderate |
| Miranda 2015 | 129 AD and AD+CVD patients (97 completed the study) | Donepezil, galantamine, rivastigmine | Clinical (probable AD, NIAA; AD+CVD, NINDS-AIREN) | Improvement of 2+ points on MMSE | 12 months | CYP2D6 is not a predictor | Moderate |
| Miranda 2017 | 42 AD and AD+CVD patients | Donepezil | Clinical (AD, NIAA; AD+CVD, NINDS-AIREN) | Less than 1 point loss on MMSE | 12 months | CYP2D6 is not a predictor | Low |
| Chianella 2011 | 171 AD patients | Donepezil, galantamine, rivastigmine | Clinical (probable AD, NINCDS-ADRDA) | Less than 1.5 corrected MMSE points loss | 12 months | CYP2D6 is not a predictor | Low |
| Pilotto 2009 | 127 AD patients (115 included at follow-up) | Donepezil | Clinical (probable AD, NINCDS-ADRDA) | No deterioration in ADAS-Cog and MMSE, improvement on ADL or IADL | 6 months | rs1080985 G allele SNP of CYP2D6 is a predictor of poor response | Low |
| Albani 2012 | 415 AD patients (68% w) | Donepezil | Clinical (Probable AD, NINCDS-ADRDA) | Improvement or no deterioration in MMSE | 6 months | C-allele on rs1080985 CYP2D6 SNP; G-allele predicts poor response, (with a marginally significant effect of the association with APOE-e4). | Low |
| Chou 2021 | 40 AD patients | Donepezil | Clinical (probable AD, NINCDS-ADRDA) | Non-worsening of CDR | 2 years | CYP2D6 SNP rs1080985 G/G (C/C and C/G are negative predictors) | Low |
| Liu 2014 | 208 AD patients | Donepezil 5 mg | Clinical (probable AD, NINCDS-ADRDA) | No deterioration in MMSE | 6 months | rs1080985 SNP of CYP2D6 is not a predictor | Low |

Table S3. Rate of progression.

| Reference | Population | Treatment | Diagnosis | Response criteria | Follow-up | Predictor | Risk of bias |
| --- | --- | --- | --- | --- | --- | --- | --- |
| Wallin 2009 | 191 AD patients (161 with CSF biomarkers) | Donepezil, galantamine, rivastigmine | Clinical (probable or possible AD, NINCDS-ADRDA, and DSM-IV) | 2+ points improvement in MMSE (other models: 2+ points improvement in MMSE & CIBIC 1-3; 2+ points improvement in ADAS-Cog; 4+ points improvement in ADAS-Cog | 6 months | Faster pre-treatment progression rates | Moderate |
| Farlow 2001 | 187 AD patients | Rivastigmine | Not stated (ref. 20 not accessible) | Change in ADAS-Cog, PDS, GDS, MMSE | 26 weeks | Rapid progression (at least 4 points deterioration on the ADAS-Cog or at least 10% worsening on PDS during 26 weeks) | High |
| Sobow 2007 | 54 AD patients | Rivastigmine | Clinical (AD, NINCDS-ADRDA, not stated if possible included; exclusion of patients who also fulfilled criteria for other dementia syndromes) | 3+ points improvement on ADAS-Cog (stability if less than 2 points variation) | 6 months | Fast progressors (3+ points/year loss on MMSE) | Moderate |

Table S4. Cardiovascular risk factors

| Reference | Population | Treatment | Diagnosis | Response criteria | Follow-up | Predictor | Risk of bias |
| --- | --- | --- | --- | --- | --- | --- | --- |
| Connelly 2005 | 166 AD patients | Not stated, probably donepezil, galantamine, rivastigmine | Clinical (probable AD, NINCDS-ADRDA) | Composite measure of change in MMSE, IADL/SB scores in NOSGER and agreement between patient, carer and doctor on global outcome - not clearly stated, probably analogous to the other studies by Connelly | 6 months | Smoker status is not a predictor | High |
| Gallucci 2015 | 84 AD patients + 6 AD+CVD patients | Donepezil, galantamine, rivastigmine | Clinical (NINCDS-ADRDA and NINDS-AIREN, not stated if possible included) | Less than 2 points deterioration of MMSE per year | Up to 4 years | Not smoking, not drinking alcohol | High |
| Connelly 2019 | 24 AD patients | Donepezil, galantamine, rivastigmine | Clinical (probable AD, ICD-10 criteria, diagnosis by a psychiatrist) | Change in MMSE, NOSGER subscales, DSST and global assessment of change including carer/family views as recommended by NICE (Ref 18) - not clear | 6 months | Greater BMI is a predictor of poor response | High |
| Fukui 2005 | 55 AD patients (50 completed the studies) | Donepezil 5 mg | Clinical (probable AD, ICD-10 and NINCDS-ADRDA) | Improvement of 2+ points on the Clock-drawing test (ref. 15 for scoring) for true responders, +- 2 points on the CDT for unchanged | 12 months | High blood pressure; hypercholesterolemia is a predictor of poor response | Moderate |
| Ho 2016 | 87 AD patients | Rivastigmine | Not clearly stated (presumably probable AD, NINCDS-ADRDA) | No deterioration on MMSE or CDR-SB | 1 year | Hypertension is a predictor of cognitive (but not global) response | Moderate |
| Connelly 2005 | 160 AD patients (147 AD patients with full data set) | Donepezil, galantamine, rivastigmine | Clinical (probable AD, NINCDS-ADRDA) | At least two of gain of 2+ points on MMSE, improvement of the combined score IALD & social behaviour subscales of NOSGER (or maintenance of maximum score), positive global change as defined as a tripartite agreement amongst doctor, subject and carer (global impression) at 6 months | 6 months | Combined hypertension and white matter lesions are negative predictors | Moderate |
| Modrego 2009 | 54 AD patients (50 included, 43 stayed on G) | Galantamine (but switching to donepezil allowed) | Clinical (probable AD, NINCDS-ADRDA) | Changes in ADAS-Cog, NPI and DAD, stroke, death | 6 months | Lower IMT (especially in men); hypertension is not a predictor | Low |
| Borroni 2003 | 120 AD patients (104 included in the analysis) | Donepezil, rivastigmine | Clinical (Probable AD, NINCDS-ADRDA) | Difference in change of MMSE | 1 year | Lower serum colesterol (under 220 mg/dL) | Low |

Table S5. Other clinical predictors.

| Reference | Population | Treatment | Diagnosis | Response criteria | Follow-up | Predictor | Risk of bias |
| --- | --- | --- | --- | --- | --- | --- | --- |
| Perera 2014 | 2460 patients | Donepezil, galantamine, rivastigmine | Not stated | Change in MMSE | 4 years (1 year prior to AChEI initiation to 3 years after) | Vascular dementia, antipsychotics, gastrointestinal drugs, and anti-platelet and anticoagulants are a predictor of poor response; DLB is not a predictor | High |
| Wattmo 2017 | 1017 AD patients | Donepezil, galantamine, rivastigmine | Clinical (possible or probable AD, NINCDS-ADRDA, and DSM-IV) | No deterioration in MMSE | 3 years | No antipsychotic use in late-onset AD patients; early onset and late onset AD are in general not a predictor | High |
| Wattmo 2011 | 843 AD patients | Donepezil, galantamine, rivastigmine | Clinical (possible or probable AD, NINCDS-ADRDA, and DSM-IV) | Not clearly stated, changes in MMSE and ADAS-Cog | 3 years | NSAID/ASA therapy | High |
| Wattmo 2012 | 784 AD patients | Donepezil, galantamine, rivastigmine | Clinical (possible or probable AD, NINCDS-ADRDA, and DSM-IV) | No deterioration in IADL or PSMS at 6 months | 3 years | Fewer antidepressants, less number of medications (for basic ADL), NSAIDs/ASA use (for response at 6 months) | High |
| Raschetti 2005 | 5642 AD patients (2853 completed the study) | Donepezil, galantamine, rivastigmine | Clinical (probable AD, NINCDS-ADRDA) | 2+ points improvement on MMSE | 9 months | Absence of concomitant diseases | Moderate |
| Tei 2008 | 50 AD patients and 56 HC | Donepezil 5 mg | Clinical (probable AD, NINCDS-ADRDA) | 4+ points improvement on MMSE | 16 weeks | IGF-I >= 110 ng/mL and MMSE >=15 | Moderate |
| Yamagata 2010 | 23 AD patients (non responders to donepezil 5 after 15 months of therapy) | Donepezil | Clinical (probable AD, NINCDS-ADRDA) | No deterioration in MMSE | 12 weeks | IGF-I <= 99 ng/mL & MMSE <= 18 in patients non responders to donepezil 5 mg (and increased to 10 mg) | Moderate |
| Kapaki 2006 | 28 AD patients (19 included in the follow-up analysis) and 24 age and sex-matched controls | Donepezil | Clinical (Probable AD, NINCDS-ADRDA) | No worsening in MMSE | 4 months | Higher T4 and fT4 pre-treatment | Moderate |
| Chang 2018 | 21 AD patients and 20 healthy controls | Donepezil 5 mg | Clinical (Probable AD, NINCDS-ADRDA) | Stabilization or improvement of MMSE (no worsening of 2+ points) | 6 months | Higher T4 pre-treatment | Moderate |
| Cho 2018 | 165 AD patients | Donepezil, rivastigmine | Clinical (DSM-IV) | Rate of decline in MMSE or CASI | 2 years | Vitamin B12 lower than 436 ng/L is a negative predictor | High |
| Modrego 2008 | 34 AD patients (22 completed the study) | Galantamine | Clinical (probable AD, NINCDS-ADRDA, and DSM-IV) | Not clearly stated, changes in ADAS-Cog, NPI, DAD | 6 months | Lower baseline plasmatic Aβ-40 | Moderate |
| Sobow 2007 | 54 AD patients | Rivastigmine | Clinical (AD, NINCDS-ADRDA, not stated if possible included; exclusion of patients who also fulfilled criteria for other dementia syndromes) | 3+ points improvement on ADAS-Cog (stability if less than 2 points variation) | 6 months | More pronounced increase in plasma Aβ-42 1 month after treatment with rivastigmine | Moderate |
| Graff-Radford 2012 | 54 DLB patients | Donepezil, galantamine, rivastigmine | Clinical (probable DLB, McKeith 2005) | Reliable change in Dementia Rating Scale: reliable improvement with 9+ points increase in <15 months and 10+ >15 months, reliable decline 6+ decrease in <15 months and 7+ decrease >15 months, stability in between | 1 year | Negative PiB PET in patients with DLB | Low |
| Wallin 2009 | 191 AD patients (161 with CSF biomarkers) | Donepezil, galantamine, rivastigmine | Clinical (probable or possible AD, NINCDS-ADRDA, and DSM-IV) | 2+ points improvement in MMSE (other models: 2+ points improvement in MMSE & CIBIC 1-3; 2+ points improvement in ADAS-Cog; 4+ points improvement in ADAS-Cog | 6 months | CSF biomarkers are not a predictor | Moderate |

Table S6. APOE status.

| Reference | Population | Treatment | Diagnosis | Response criteria | Follow-up | Predictor | Risk of bias |
| --- | --- | --- | --- | --- | --- | --- | --- |
| Ho 2016 | 87 AD patients | Rivastigmine | Not clearly stated (presumably probable AD, NINCDS-ADRDA) | No deterioration on MMSE or CDR-SB | 1 year | APOE-ε4 is not a predictor | Moderate |
| Bullock 2006 | 994 AD patients (moderately-severe), 578 completed the study | Donepezil, rivastigmine | DSM-IV criteria and NINCDS-ADRDA | Severe impairment battery, no clear definition. | 2 years | APOE-ε4 is not a predictor | Some concerns - Moderate |
| De Beaumont 2016 | 128 MCI patients | Donepezil | Clinical (aMCI subjects according to ref. 46) | ADAS-Cog change | 3 years | APOE-ε4 is not a predictor | Moderate |
| Clarelli 2016 | 169 AD patients (a subset with MMSE > 19 was selected to be consistent with Braga2015) | Donepezil, galantamine, rivastigmine | Clinical (probable AD, NINCDS-ADRDA) ref.11 | Non-worsening of MMSE (as in Braga2015) and improvement of 2+ points in MMSE (as in Weng) | 1 year | APOE-ε4 is not a predictor | Low |
| Klimkowicz-Mrowiec 2011 | 101 AD patients | Donepezil, rivastigmine | Clinical (probable AD, NINCDS-ADRDA) | No deterioration in MMSE and CDT, global improvement on IADL | 9 months | APOE-ε4 is not a predictor | Moderate |
| Liu 2014 | 208 AD patients | Donepezil 5 mg | Clinical (probable AD, NINCDS-ADRDA) | No deterioration in MMSE | 6 months | APOE-ε4 is not a predictor | Low |
| Miranda 2017 | 42 AD and AD+CVD patients (55 with donepezil, but 13 D 5) | Donepezil | Clinical (AD, NIAA, ref 20; AD+CVD, NINDS-AIREN) | Less than 1 point loss on MMSE | 12 months | APOE-ε4 is not a predictor | Low |
| Ma 2019 | 174 AD patients (with patients treated with R as a control group) | Donepezil, galantamine, rivastigmine | Clinical (probable and possible AD, NINCDS-ADRDA) | No deterioration in ADAS-Cog and MMSE | 1 year | APOE-ε4 is not a predictor | High |
| Zhong 2012 | 110 AD patients (96 completed the study) | Donepezil | Clinical (probable AD, NINCDS-ADRDA) | 2+ points improvement in MMSE (but it also states "no deterioration", so not so clear) | 6 months | APOE-ε4 is not a predictor | Moderate |
| Miranda 2015 | 129 AD and AD+CVD patients (97 completed the study) | Donepezil, galantamine, rivastigmine | Clinical (probable AD, NIAA, ref 10; AD+CVD, NINDS-AIREN) | Improvement of 2+ points on MMSE | 12 months | APOE-ε4 is not a predictor | Moderate |
| Pilotto 2009 | 127 AD patients (115 included at follow-up) | Donepezil | Clinical (probable AD, NINCDS-ADRDA) | No deterioration in ADAS-Cog and MMSE, improvement on ADL or IADL | 6 months | APOE-ε4 is not a predictor | Low |
| Lin 2019 | 33 AD patients | Galantamine 8 mg | Clinical (NINCDS-ADRDA, not stated if possible included) | No deterioration in MMSE, CASI and CDR-SB (doesn't state if only one is needed) | 6 months | APOE-ε4 is not a predictor | Low |
| Wallin 2009 | 191 AD patients (161 with CSF biomarkers) | Donepezil, galantamine, rivastigmine | Clinical (probable or possible AD, NINCDS-ADRDA, and DSM-IV) | 2+ points improvement in MMSE (other models: 2+ points improvement in MMSE & CIBIC 1-3; 2+ points improvement in ADAS-Cog; 4+ points improvement in ADAS-Cog | 6 months | APOE-ε4 is not a predictor | Moderate |
| Sobow 2007 | 54 AD patients | Rivastigmine | Clinical (AD, NINCDS-ADRDA, not stated if possible included; exclusion of patients who also fulfilled criteria for other dementia syndromes) | 3+ points improvement on ADAS-Cog (stability if less than 2 points variation) | 6 months | APOE-ε4 is not a predictor | Moderate |
| Graff-Radford 2012 | 54 DLB patients | Donepezil, galantamine, rivastigmine | Clinical (probable DLB, McKeith 2005) | Reliable change in Dementia Rating Scale: reliable improvement with 9+ points increase in <15 months and 10+ >15 months, reliable decline 6+ decrease in <15 months and 7+ decrease >15 months, stability in between | 1 year | APOE-ε4 is not a predictor | Low |
| Rigaud 2002 | 117 AD patients (80 completed the study) | Donepezil | Clinical (probable AD, NINCDS-ADRDA and DSM-IV) | Change in ADAS-Cog (main outcome), MMSE, IADL, Caregiver-rated Clinical Global Impression of Change | 36 weeks | APOE-ε4 is not a predictor | Moderate |
| Farlow 2004 | 367 AD patients | Rivastigmine | Not stated (ref. 11 not accessible) and probable AD (DSM-IV and NINCDS-ADRDA) | Change in ADAS-Cog | 26 weeks | APOE-ε4 is not a predictor | Some concerns – Low |
| Waring 2015 | 391 AD patients (ITT; 287 completer) | Donepezil | Clinical (probable AD, NINCDS-ADRDA) | Change in ADAS-Cog | 12 weeks | APOE-ε4 is not a predictor | Low |
| Aerssens 2000 | 310 (3 months) and 543 (6 months) Caucasian AD patients | Galantamine 24-36 mg | Clinical (Probable AD, NINCDS-ADRDA) | Mean change from baseline in total ADAS-cog score | 3-6 months | APOE-ε4 is not a predictor | Some concerns – Low |
| Visser 2005 | 121 AD patients | Rivastigmine | Clinical (probable AD, NINCDS-ADRDA and DSM-IV) | 2+ points improvement on MMSE or 4+ points improvement on ADAS-Cog | 26 weeks | APOE-ε4 is not a predictor | Low |
| McGowan 1998 | 39 AD patients | Galantamine | Clinical (probable AD, NINCDS-ADRDA) | No deterioration in MMSE | 3 months | Male APOE-ε4 carriers | Low |
| Wattmo 2011 | 843 AD patients | Donepezil, galantamine, rivastigmine | Clinical (possible or probable AD, NINCDS-ADRDA, and DSM-IV) | Not clearly stated, changes in MMSE and ADAS-Cog | 3 years | APOE-ε4 non-carriers | High |
| Csernansky 2005 | 39 AD patients (37 completed the study) | Donepezil | Clinical (NINCDS-ADRDA) - doesn't state whether it's probable or possible | Rate of change in ADAS-Cog (primary outcome); CDR, MMSE, NPI (secondary outcome) - ref. 21, 22 | 2 years | APOE-ε4 allele number is a predictor of poor CDR-SB response | High |
| Braga 2014 | 177 AD patients (at 6 months), 147 AD patients (at 2 years) | Donepezil, galantamine, rivastigmine | Clinical (probable AD, NINCDS-ADRDA) | MMSE improvement or no deterioration | 2 years | APOE-ε4 non-carriers | Low |
| Choi 2008 | 51 AD patients | Donepezil | Clinical (probable AD, NINCDS-ADRDA) | Not clearly stated, change in several tests | 48 weeks | APOE-ε4 carriers | High |
| Chen 2017 | 63 AD patients | Rivastigmine | Clinical (NINCDS-ADRDA, not stated if probable or possible) | Improvement in either MMSE or CDR | 6 months | APOE-ε4 carriers | Moderate |
| Devanand 2017 | 37 MCI patients (32 patients completed the study) | Donepezil (Rivastigmine or galantamine if not tolerated) | Clinical (aMCI with subjective memory complaints, score > 1.5 SD below norms on either WMS-III Logical memory subtest immediate or delayed recall, or the Free and Cued Selective Reminding Test immediate or delayed recall, no functional impairment consistent with dementia, MMSE 23+, CDR 0.5) | ADAS-Cog and Selective Reminding Test change; CDR, consensus diagnosis, CIBIC-plus and FAQ as secondary outcomes | 52 weeks | APOE-ε4 carriers | Low |
| Patterson 2011 | 165 AD patients (81 completed the whole study) | Donepezil, galantamine, rivastigmine | Clinical (probable AD, NINCDS-ADRDA and DSM-IV) | Improvement in MMSE at 3-9 months (early cognitive response); no decline in MMSE at 15-24 months (late cognitive response) | Up to 24 months | APOE-ε4 carriers in MMSE>21, BCHE-K in MMSE <= 15 | Moderate |
| Blesa 2006 | 167 AD patients | Rivastigmine 6 mg or less | Clinical (DSM IV, NINCDS-ADRDA) | ADAS-Cog and Blessed Dementia Scale | 6 months | Greater efficacy on anxiety-related behavioral symptoms in APOE-ε4 non-carriers, and on irritability in carriers, as measured by NPI subitems; however, total NPI was not affected by APOE status | High |
| Lu 2016 | 85 AD patients | Donepezil | Not stated | No deterioration in MMSE | At least 3 months (but it's not clear when response is assessed) | APOE-ε3 non-carrier and CYP2D6*10/10 on rs1065852 SNP | High |
| Lu 2018 | 88 AD patients | Donepezil | Not stated | No deterioration in MMSE | At least 3 months | ABCA1 rs2230806 GG genotype + APOE-ε3 non-carrier | High |

Table S7. Other genes.

| Reference | Population | Treatment | Diagnosis | Response criteria | Follow-up | Predictor | Risk of bias |
| --- | --- | --- | --- | --- | --- | --- | --- |
| Paroni 2014 | 109 AD patients | Donepezil, galantamine, rivastigmine | Clinical (probable or possible AD, NINCDS-ADRDA) | No deterioration on ADAS-Cog and MMSE, improvement in ADL or IADL | 6 months | rs7981045 G/G polymorphism of FOXO1 is a predictor of poor response | Moderate |
| Lu 2018 | 88 AD patients | Donepezil | Not stated | No deterioration in MMSE | At least 3 months | ABCA1 rs2230806 GG genotype + APOE E3 non-carrier | High |
| Magliulo 2011 | 54 AD patients | Donepezil | Clinical (probable AD, NINCDS-ADRDA) | Not clearly stated (change in CIBIC-plus or in MMSE from first to second assessment) | Up to 40 months (median 9 months) | ABCB1 polymorphisms are not a predictor | High |
| Martinelli-Boneschi 2013 | 287 + 252 AD patients (176 included in the study, 198 in the replication phase) | Donepezil, galantamine, rivastigmine | Clinical (probable AD, NINCDS-ADRDA) | Less than 1 point/year loss in MMSE (non-responders: worsening of >3 points in MMSE) | 18 months | rs17798800 allele A (PRKCE) is a predictor of non-response, and rs6720975 allele A (associated with neurobeachin) is a predictor of response | Moderate |
| Scacchi 2013 | 184 AD patients | Donepezil, rivastigmine | Clinical (probable AD, NINCDS-ADRDA and DSM-IV) | Change in MMSE compared to untreated patients | 15 months | Female gender, and carriers of P and X allele on ESR1 rs2234693 and rs9340799 SNPs (only for donepezil) | Low |

Table S8. Neurophysiological predictors.

| Reference | Population | Treatment | Diagnosis | Response criteria | Follow-up | Predictor | Risk of bias |
| --- | --- | --- | --- | --- | --- | --- | --- |
| Adler 2004 | 20 AD patients | Rivastigmine | Clinical (Probable AD, NINCDS-ADRDA) | Improvement in short-term memory (SIDAM syndromes) at 6 months | 6 months | Decreased qEEG theta power after 1 week (and higher pretreatment short-term memory) | High |
| Baakman 2021 | 50 AD patients | Galantamine | Not stated | Improvement in MMSE, NPI and DAD scores | 6 months | Acute decrease of absolute frontal alpha, beta and theta EEG parameters and relative frontal theta power | High |

Table S9. Neuropsychological and behavioral predictors.

| Reference | Population | Treatment | Diagnosis | Response criteria | Follow-up | Predictor | Risk of bias |
| --- | --- | --- | --- | --- | --- | --- | --- |
| Mega 1999 | 86 AD patients | Donepezil | Clinical (probable or possible AD, NINCDS-ADRDA) | 4+ point reduction in total NPI-10 scale (frequency x severity); 4+ increase are non-responders, in-between unchanged behaviorally | 8 weeks | Worse depression, apathy, disinhibition, and irritability | High |
| Mega 2000 | 33 AD patients | Donepezil | Clinical (probable or possible AD, NINCDS-ADRDA) | 4+ point reduction in total NPI-10 scale (frequency x severity); 4+ increase are non-responders, in-between unchanged behaviorally | 8 weeks | Worse disinhibition and irritability | High |
| Tanaka 2004 | 70 AD patients | Donepezil 5 mg | Clinical (probable AD, NINCDS-ADRDA) | 4+ points improvement in NPI (unchanged: 3- variation in NPI) | 12 weeks | Dysphoria, anxiety, apathy | Moderate |
| Lemstra 2008 | 53 patients with cognitive decline and neuropsychiatric features (34 completed the study) | Rivastigmine | Clinical (AD - NINCDS-ADRDA; VD - NINDS-AIREN; DLB - McKeith; PDD - UKPD brain bank criteria) + DSM-IV | No deterioration in MMSE, IDDD (interview for deterioration in daily living) and NPI | 6 months | Fluctuation in reaction time tasks (VRT-sd) and poor sustained attention (CPT) | High |
| Kuzmickiené 2015 | 72 AD patients and 30 controls | Donepezil | Clinical (probable AD, NINCDS-ADRDA) | CANTAB test scores | 4 months | Cognitive changes produced by the first single donepezil dose in CANTAB PAL, PRM, and SWM test, and baseline CANTAB PAL, PRM, and CRT test results. | Moderate |
| Mori 2016 | 235 DLB patients | Donepezil | Clinical (probable DLB, McKeith) with NPI >7 and hallucinations NPI subitem > 0 | Change in MMSE (with LOCF imputation) | 12 weeks | Patients with MMSE subitems serial 7's scores of 1-3, delayed recall scores of >= 1 and copying scores of 0 (subgroup with typical DLB cognitive impairment pattern) | Some concerns |
| Saumier 2007 | 30 AD patients | Donepezil | Clinical (probable or possible AD, NINCDS-ADRDA and DSM-III) | Mean ratio of change among MMSE, ADAS-Cog, CIBIC-plus equal to 0 or greater | 6 months | Better performances in Clock Drawing test, a tracking speed test, and Boston Naming test | High |
| Connelly 2005 | 140 AD patients (160 enrolled) | Donepezil, galantamine, rivastigmine | Clinical (probable AD, NINCDS-ADRDA) | At least two of gain of 2+ points on MMSE, improvement of the combined score IALD & social behaviour subscales of NOSGER (or maintenance of maximum score), positive global change as defined as a tripartite agreement amongst doctor, subject and carer (global impression) at 6 months | 3 years (actually 6 months) | Higher performances on DSST test at baseline and 6 months | Moderate |

Table S10. Other predictors.

| Reference | Population | Treatment | Diagnosis | Response criteria | Follow-up | Predictor | Risk of bias |
| --- | --- | --- | --- | --- | --- | --- | --- |
| Mecocci 2002 | 61 AD patients | Donepezil 5 mg | Clinical (NINCDS-ADRDA, not stated if possible included) | Decrease of 4+ points on ADAS-Cog or CIBIC-plus score of 4- after 3 months of therapy | 3 months | TASM static ANN could help predicting response | Moderate |
